# Supplementary material for: Design, Synthesis and Structure-Activity Relationship of Novel Pinacolone Sulfonamide Derivatives against Botrytis cinerea as Potent Antifungal Agents
Source: Molecules. 2022 Aug 25;27(17):5468. doi: 10.3390/molecules27175468 (PMC9458130; doi:10.3390/molecules27175468)
Supplement: Supplementary file 1 [file molecules-27-05468-s001.zip › molecules-1849520-supplementary.pdf]

Design, Synthesis and Structure-Activity Relationship of Novel Pinacolone Sulfonamide Derivatives against *Botrytis cinerea* as Potent Antifungal Agents

Liu Chaojie, Xiang Xiaofang, Wan Ying, Yang Jia, Li Yufei, Zhang Xinchen, Qi Zhiqiu, He Lu, Liu Wei* and Li Xinghai*

Department of Pesticide Science, Plant Protection College, Shenyang Agricultural University, Shenyang 110866, PR China

***Correspondence: liuwei871016@syau.edu.cn; xinghai30@163.com.

Supplementary Materials

CONTENT

1. NMR spectra of P-1~P-46 2

2. MS of P-1~P-46 48

3. HPLC spectra of P-18, P-23, P-29, P-30, P-31..70

4. X-ray single crystal diffraction of P-27 72

1. NMR spectra of P-1~P-46

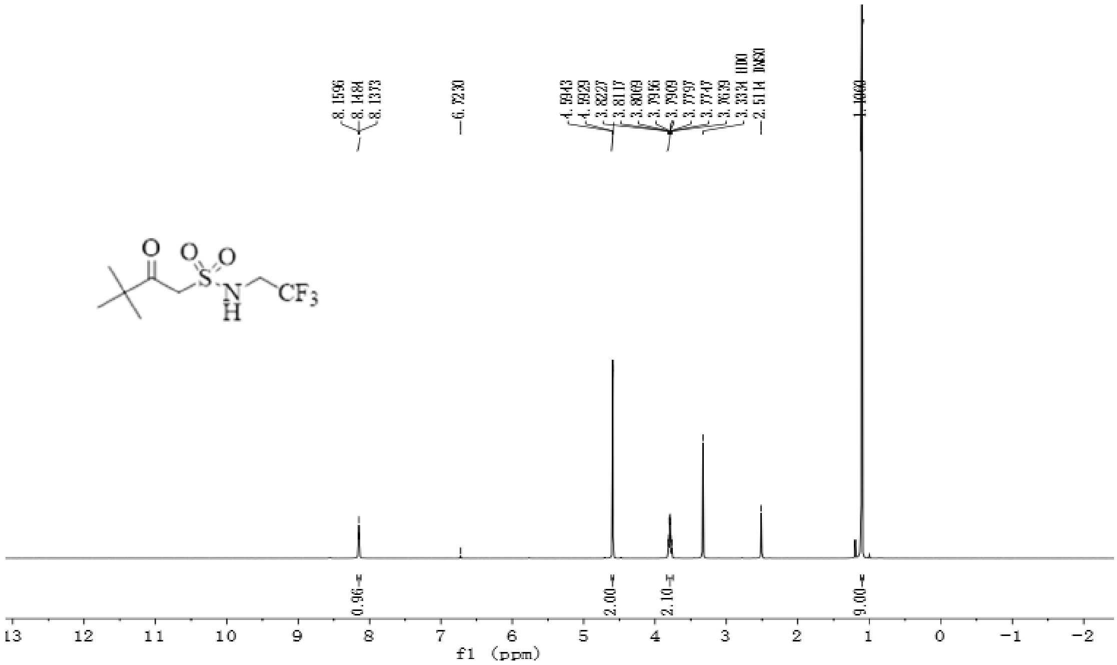

Figure S1. ^{1}H NMR of compound *P-1*

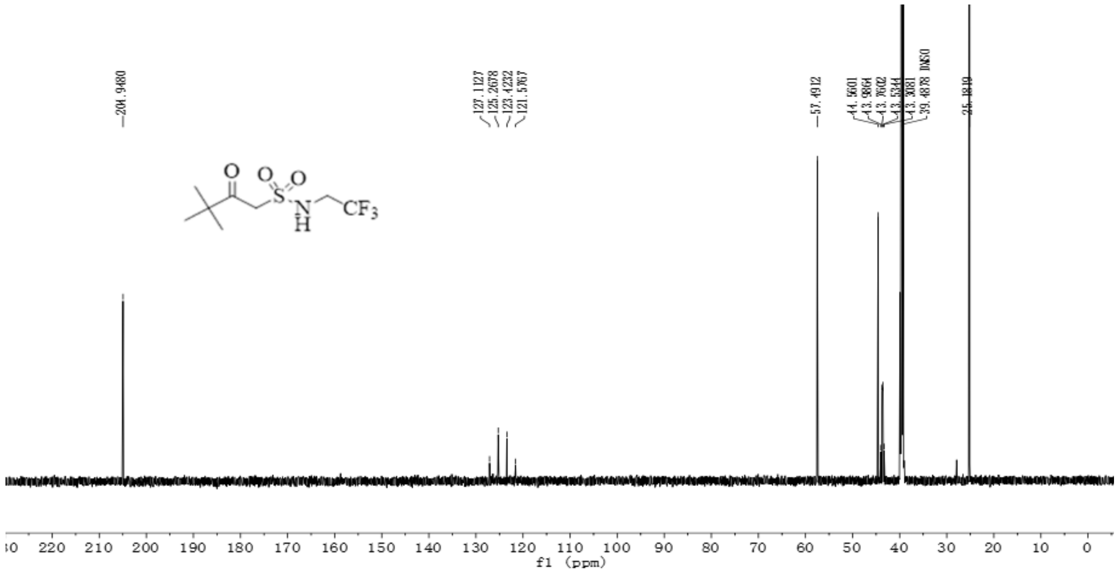

Figure S2. ^{13}C NMR of compound *P-1*

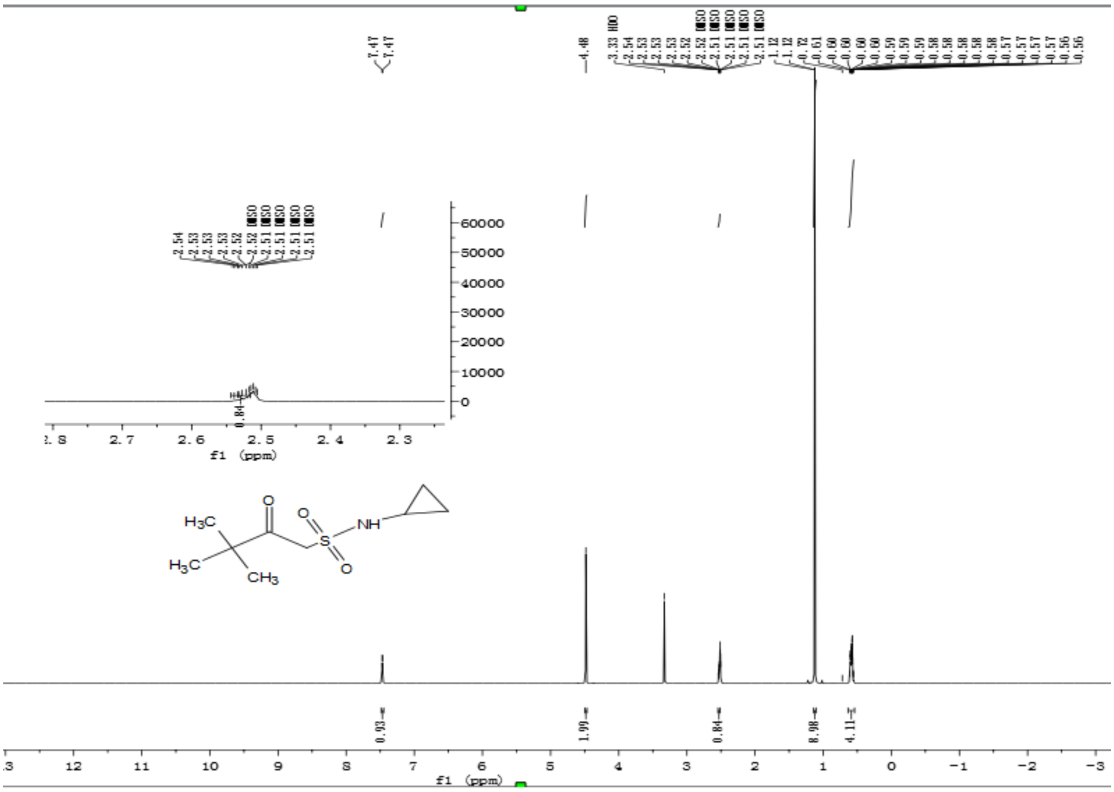

Figure S3. ^{1}H NMR of compound *P-2*

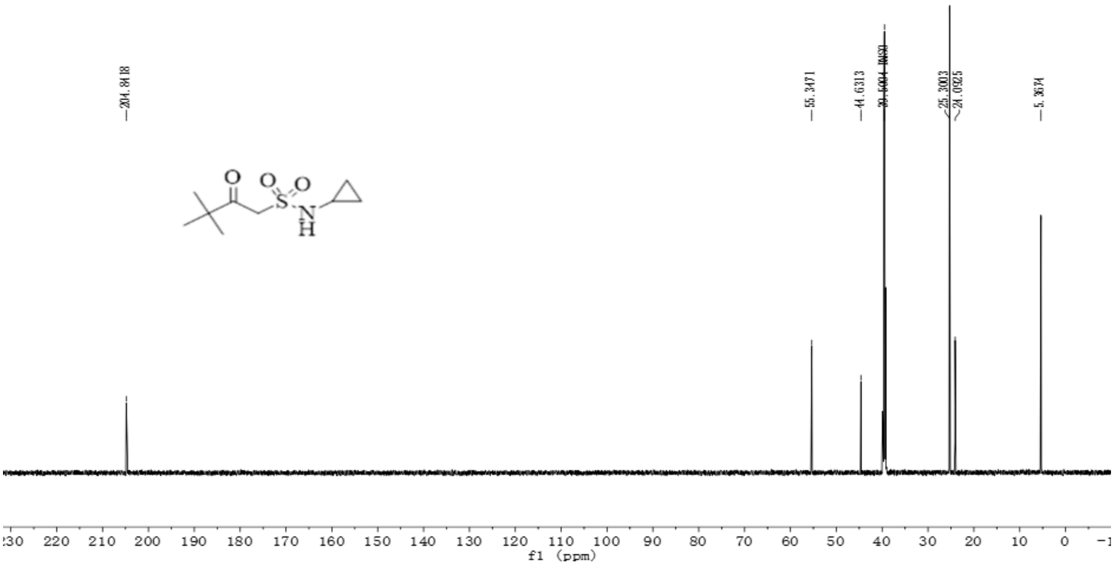

Figure S4. ^{13}C NMR of compound *P-2*

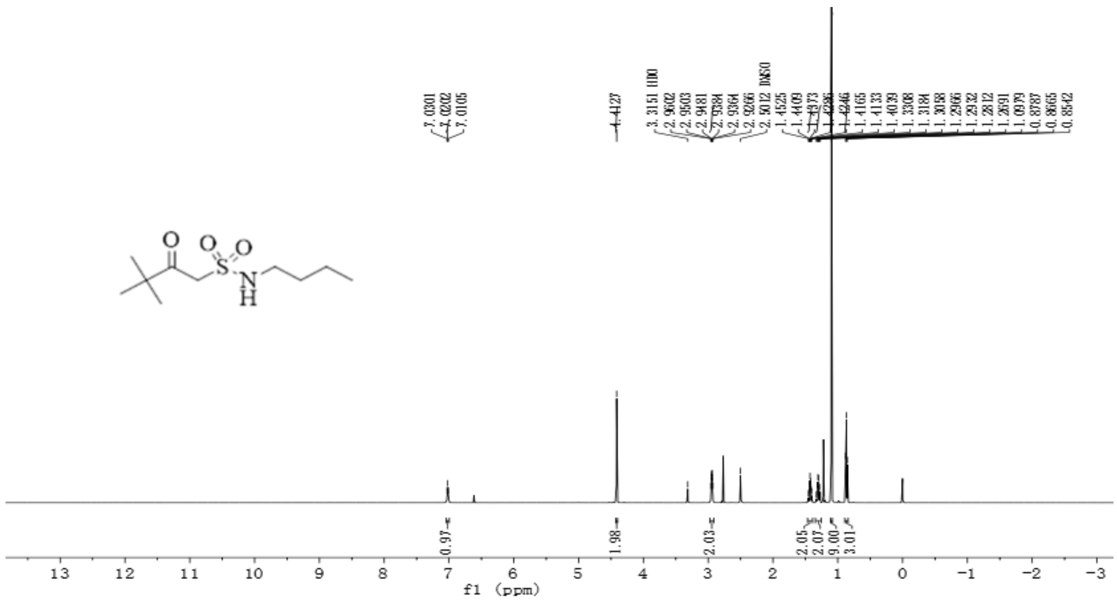

Figure S5. ^{1}H NMR of compound *P-3*

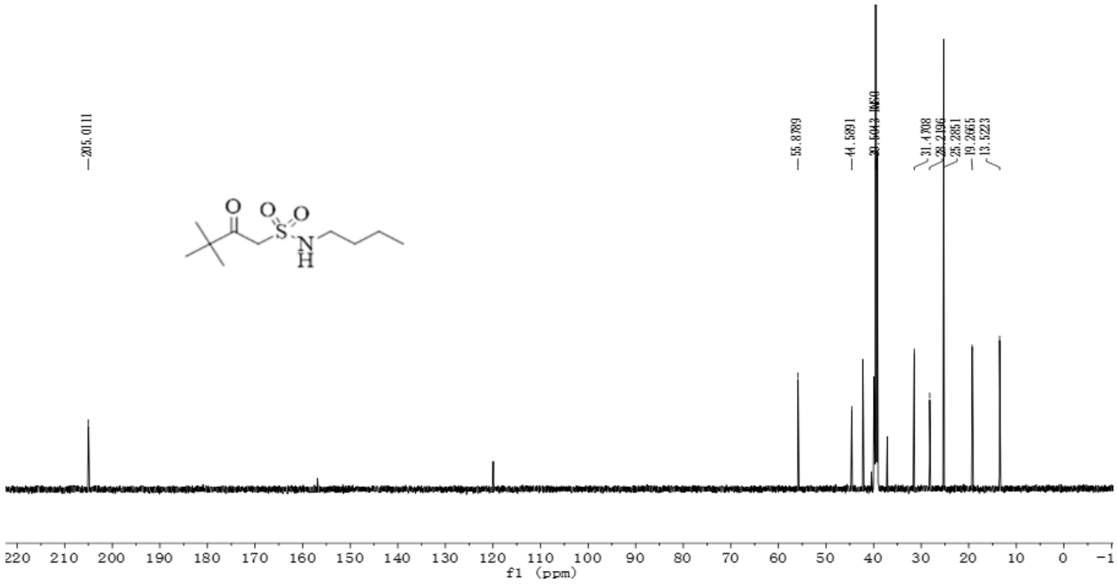

Figure S6. ^{13}C NMR of compound *P-3*

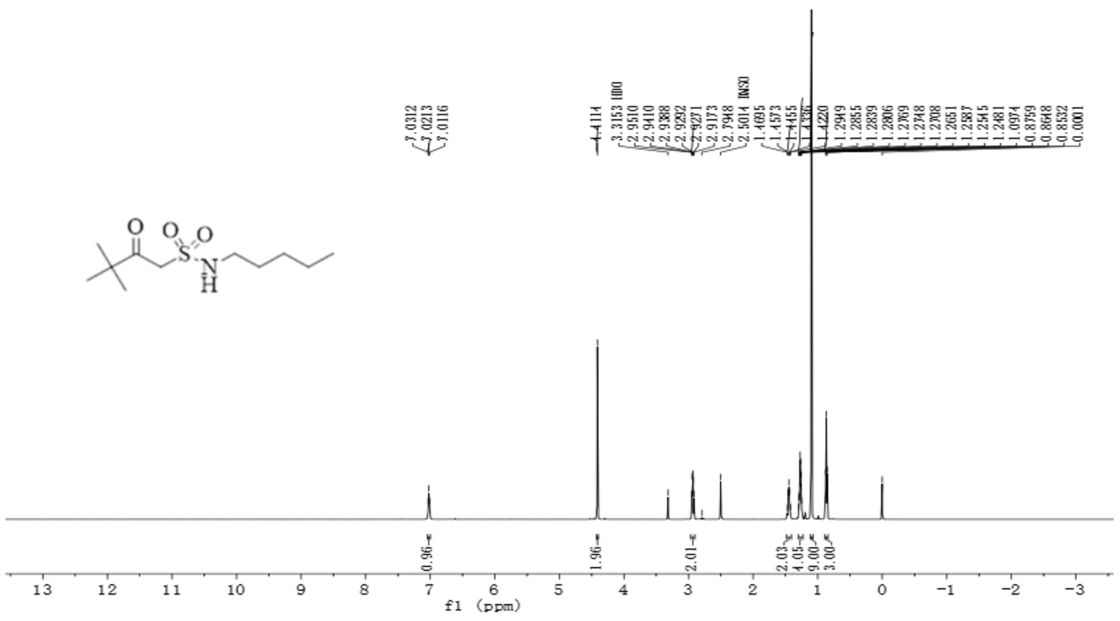

Figure S7. ^{1}H NMR of compound *P-4*

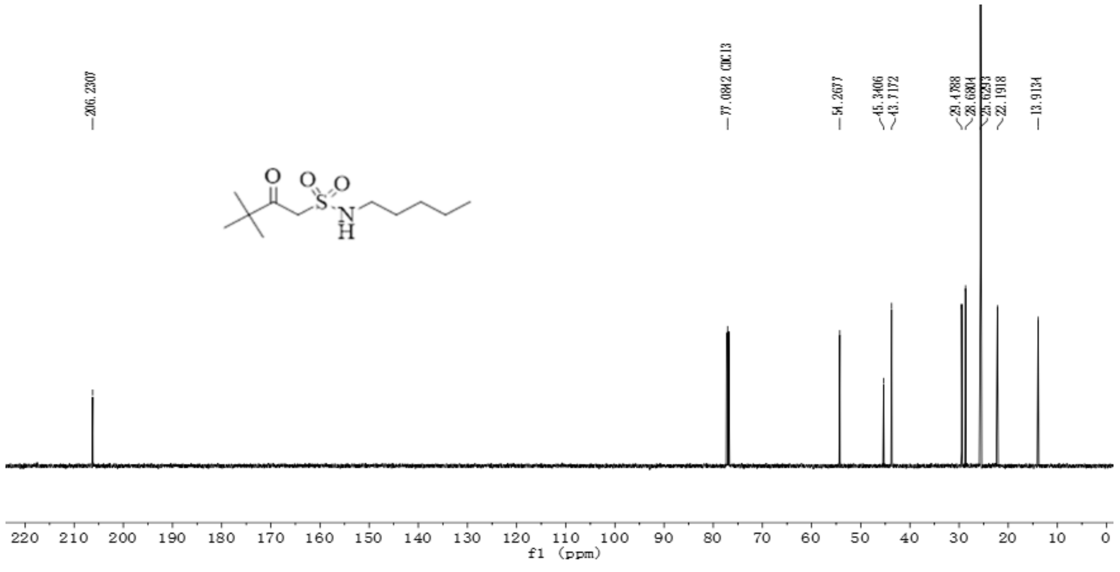

Figure S8. ^{13}C NMR of compound *P-4*

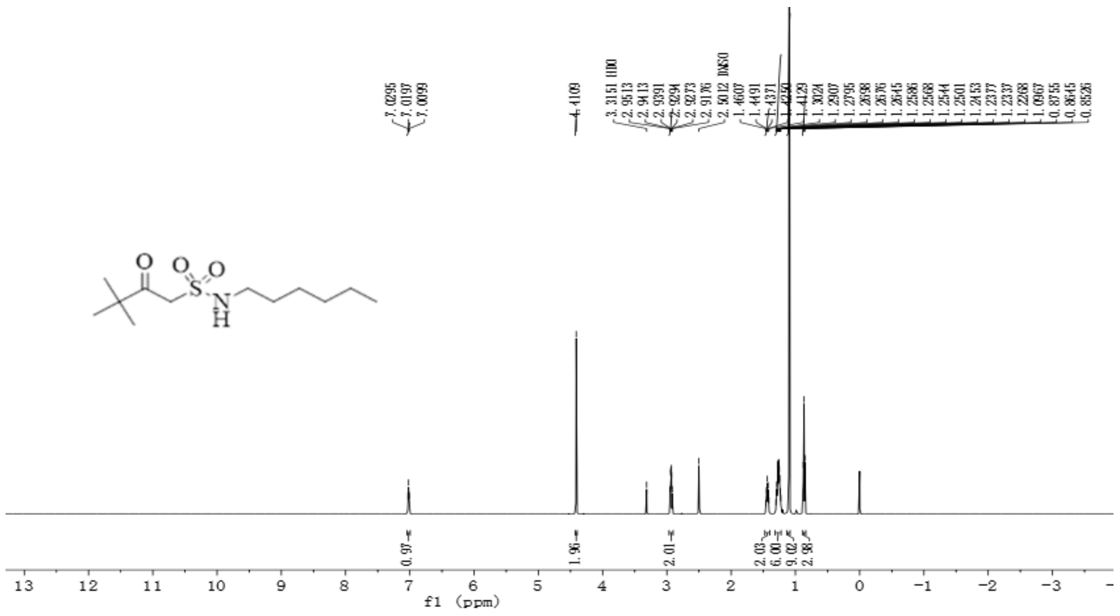

Figure S9. ^{1}H NMR of compound *P-5*

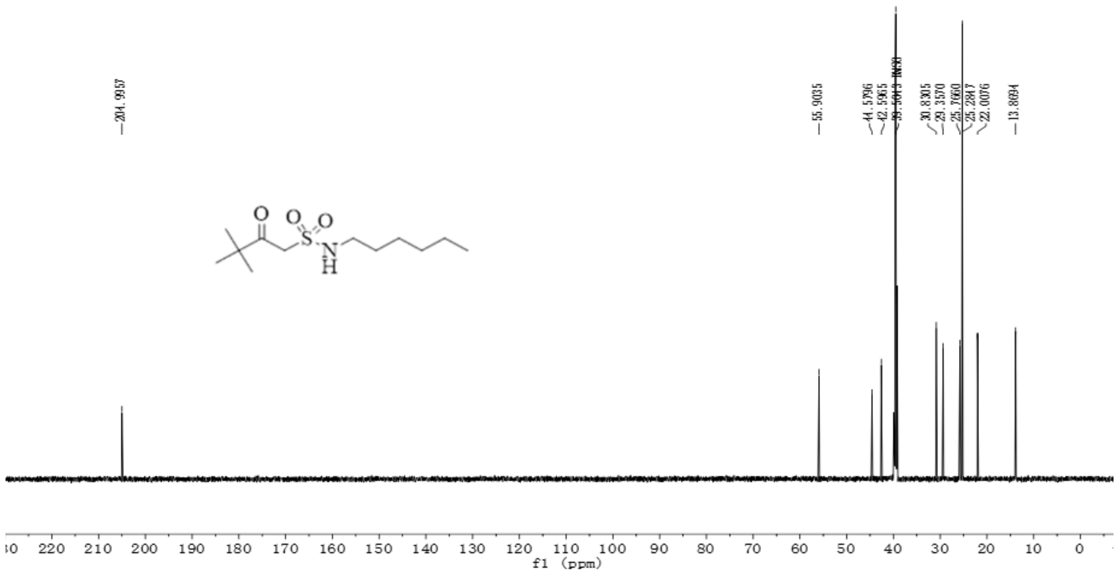

Figure S10. ^{13}C NMR of compound *P-5*

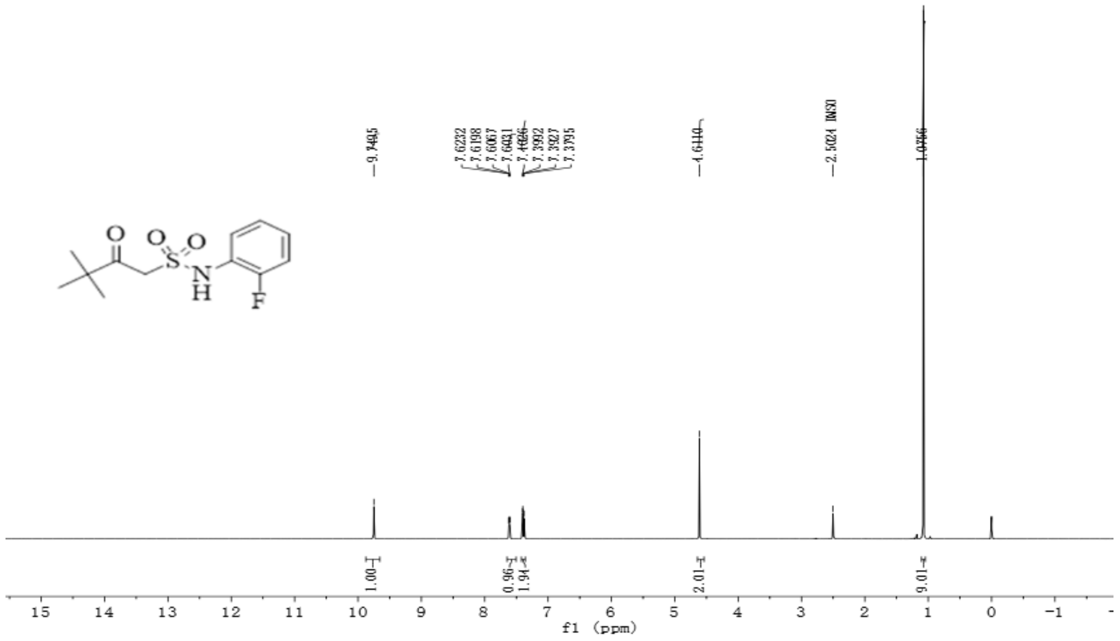

Figure S11. ^{1}H NMR of compound *P-6*

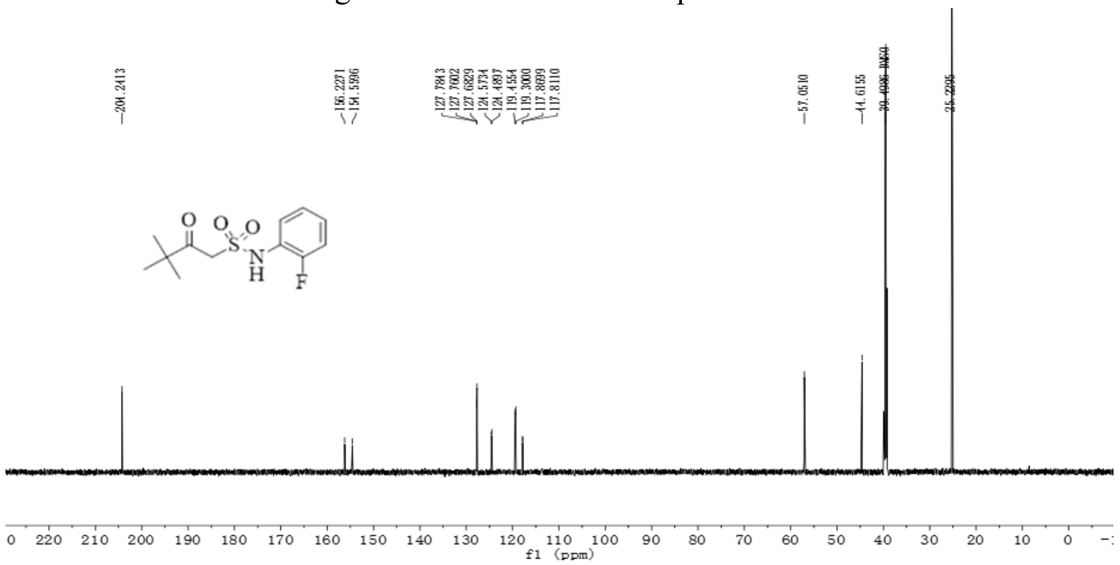

Figure S12. ^{13}C NMR of compound *P-6*

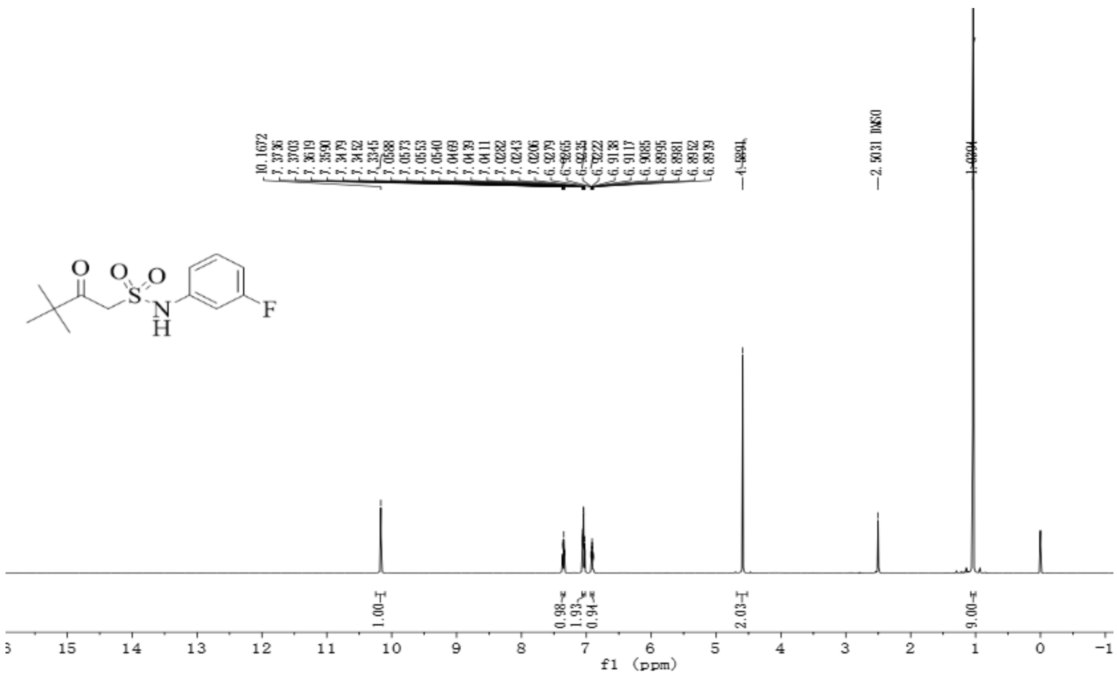

Figure S13. ^{1}H NMR of compound *P-7*

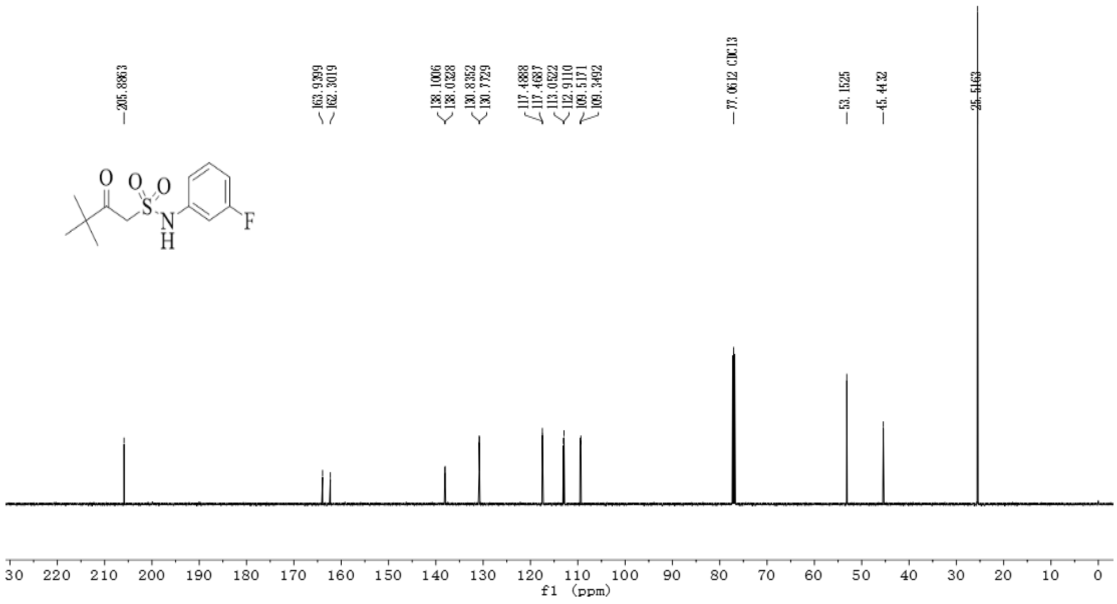

Figure S14. ^{13}C NMR of compound *P-7*

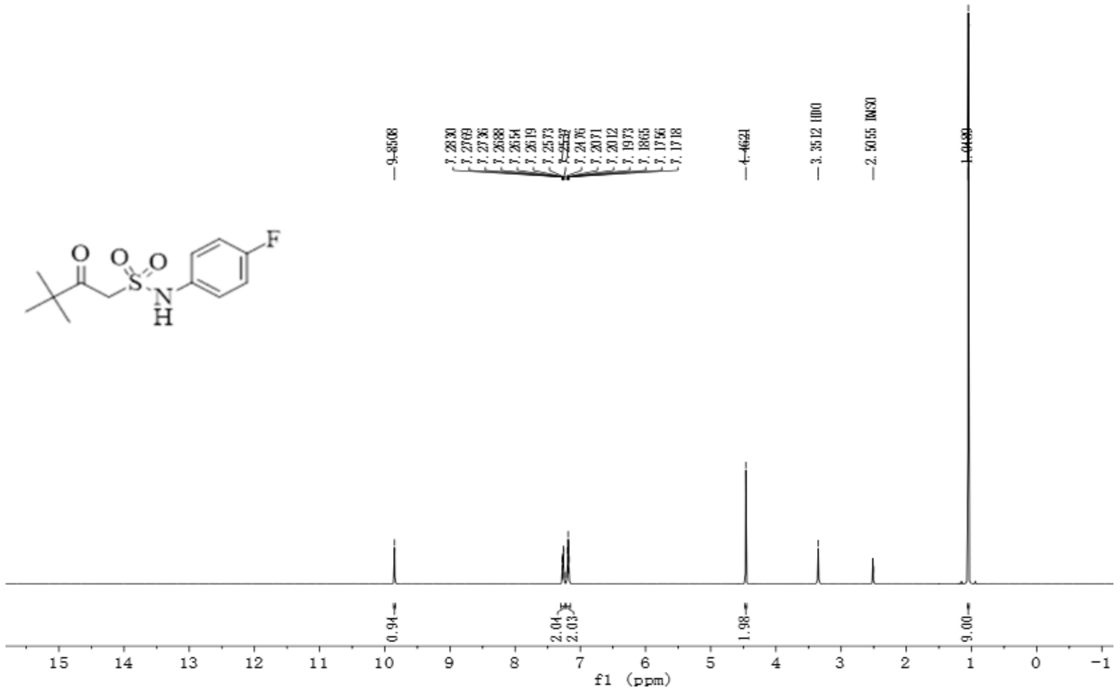

Figure S15. ^{1}H NMR of compound *P-8*

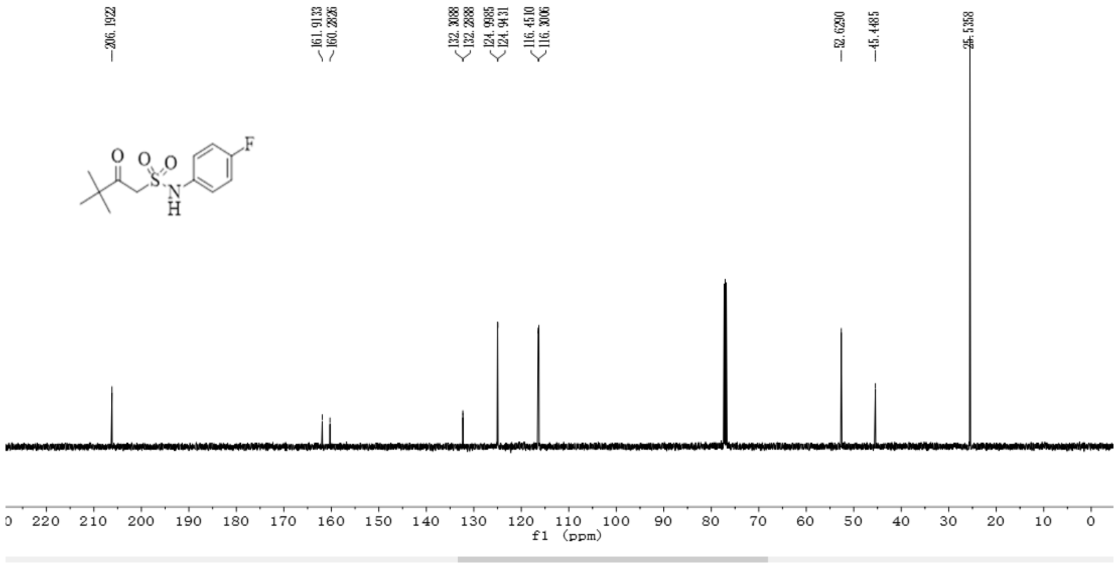

Figure S16. ^{13}C NMR of compound *P-8*

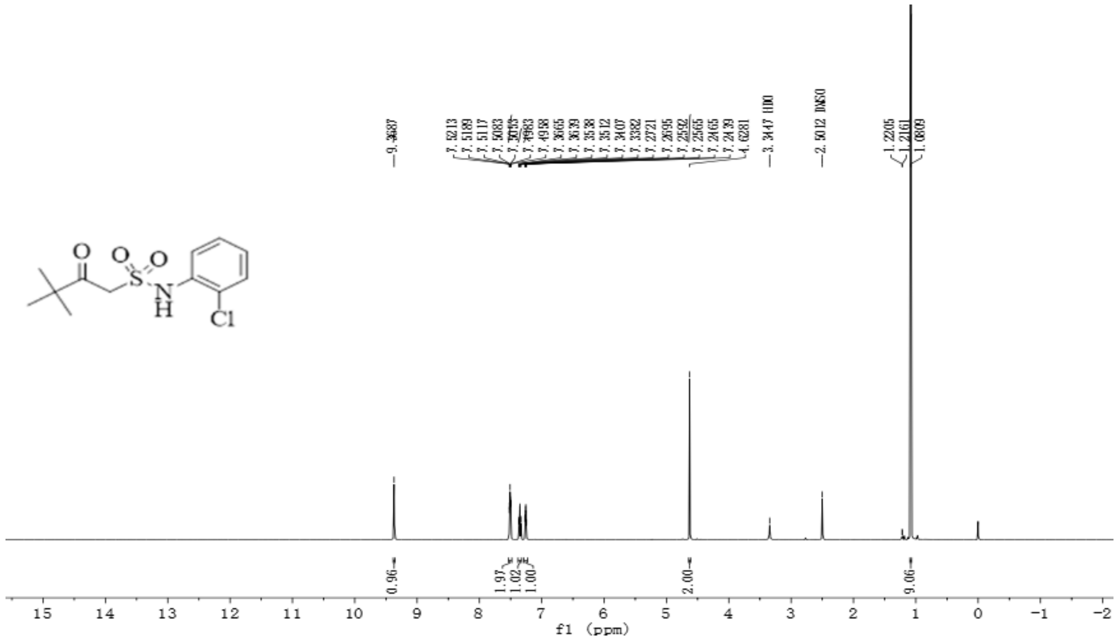

Figure S17. ^{1}H NMR of compound *P-9*

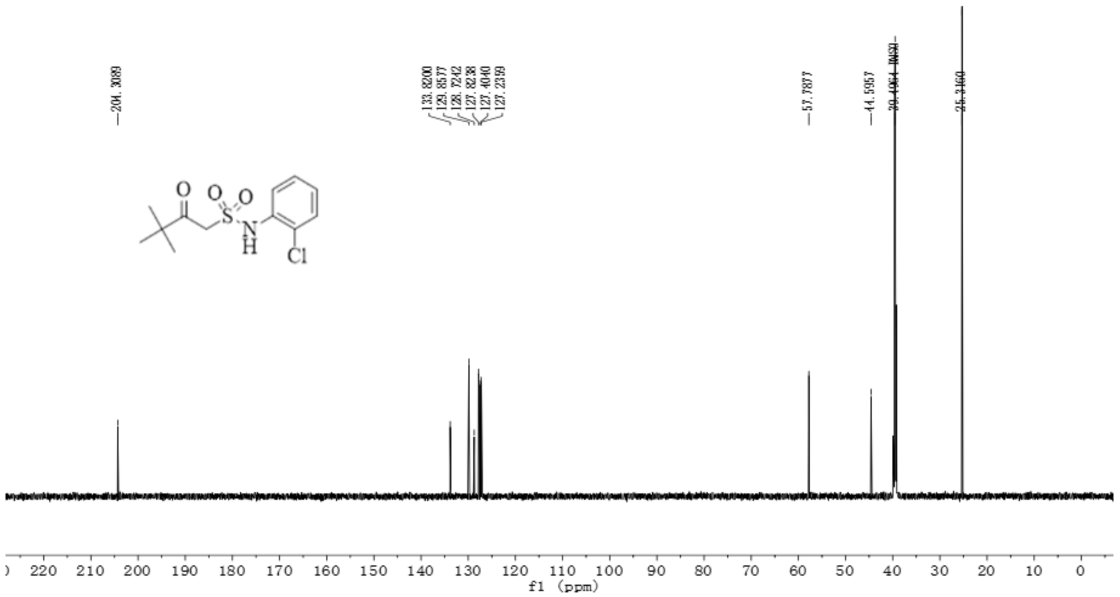

Figure S18. ^{13}C NMR of compound *P-9*

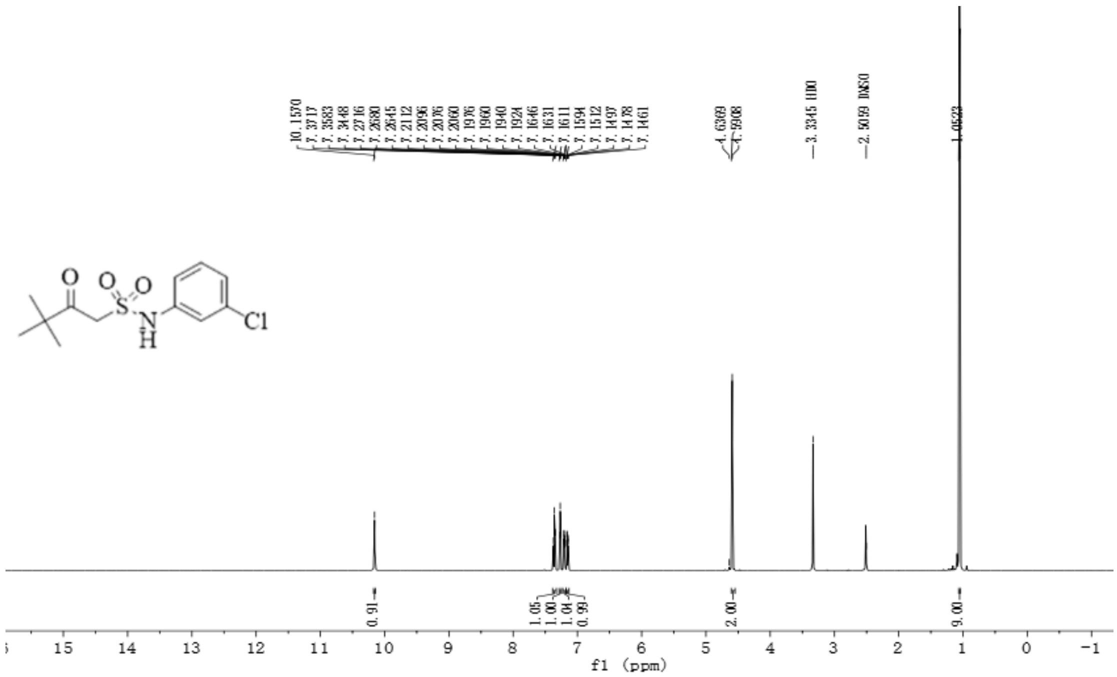

Figure S19. ^{1}H NMR of compound *P-10*

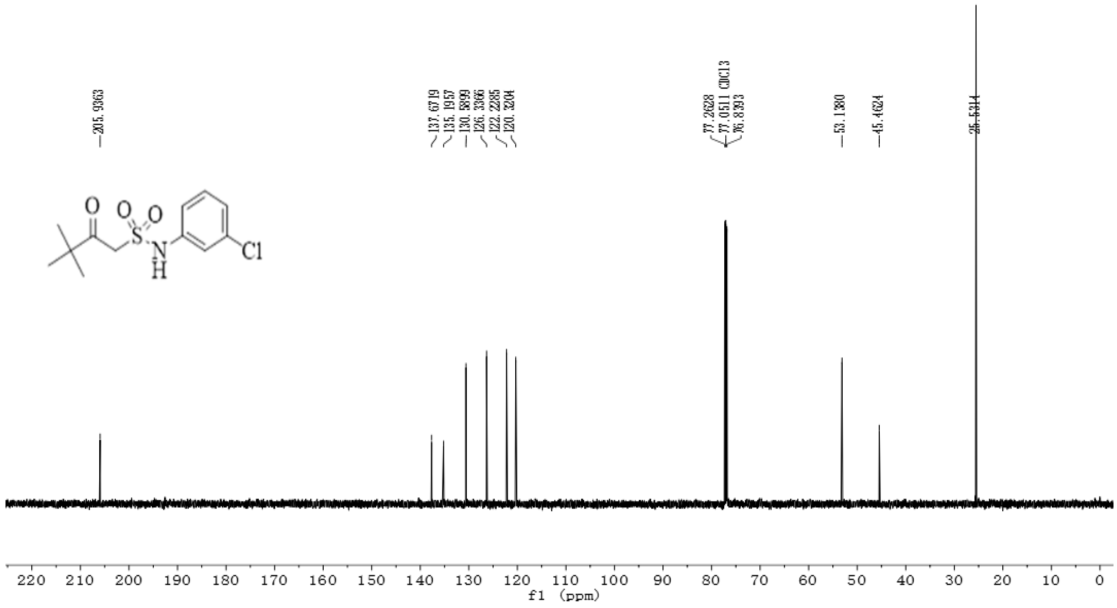

Figure S20. ^{13}C NMR of compound *P-10*

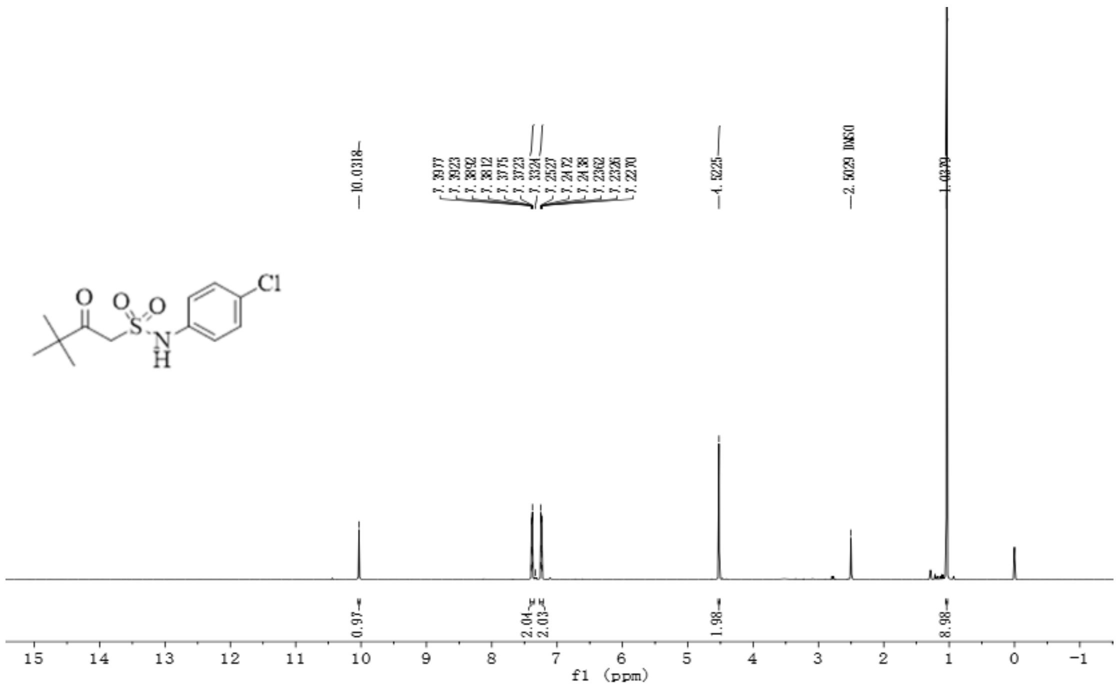

Figure S21. ^{1}H NMR of compound *P-11*

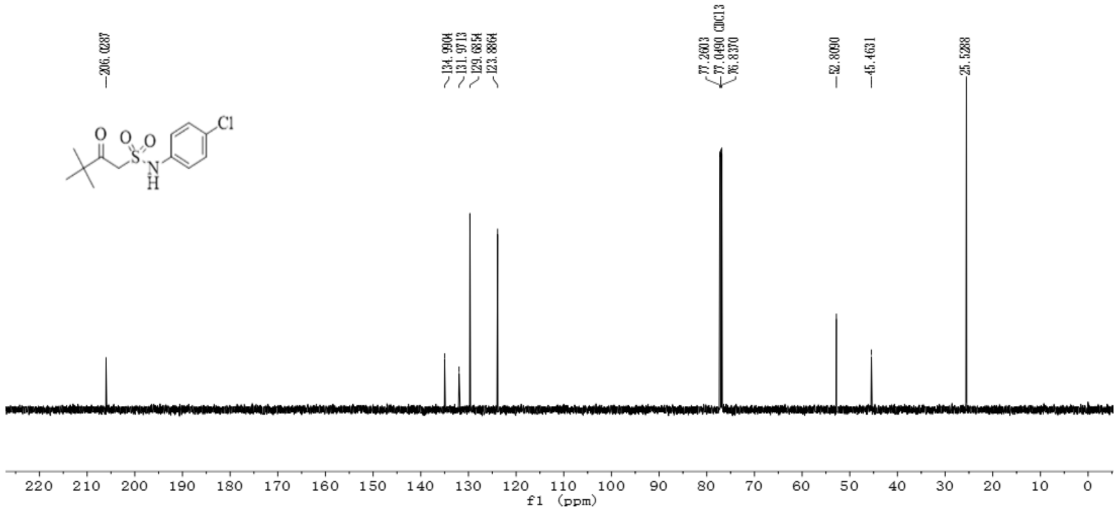

Figure S22. ^{13}C NMR of compound *P-11*

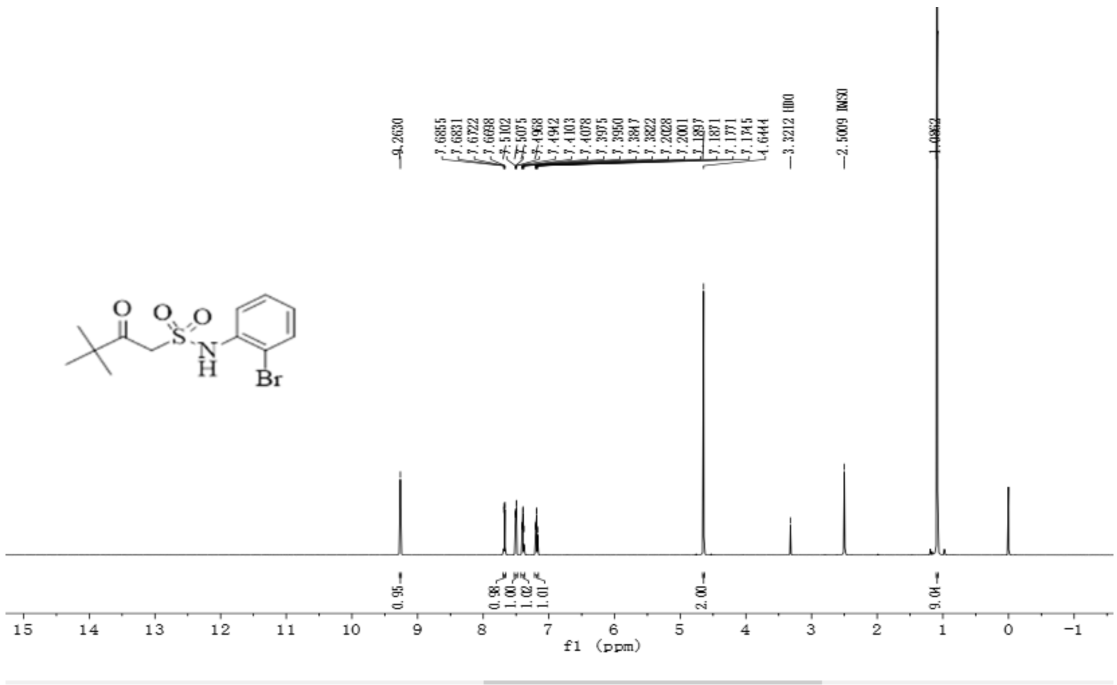

Figure S23. ^{1}H NMR of compound *P-12*

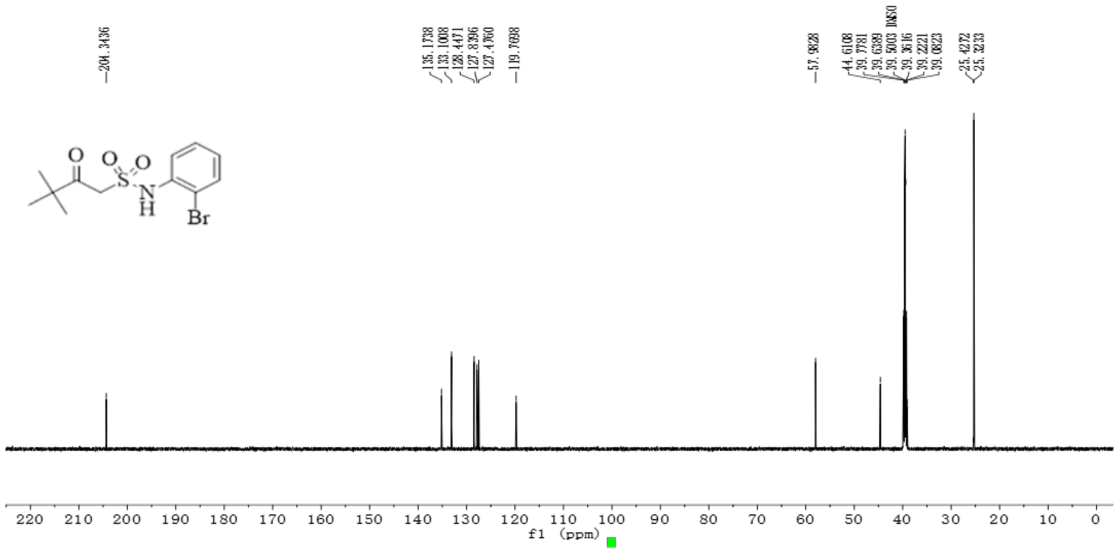

Figure S24. ^{13}C NMR of compound *P-12*

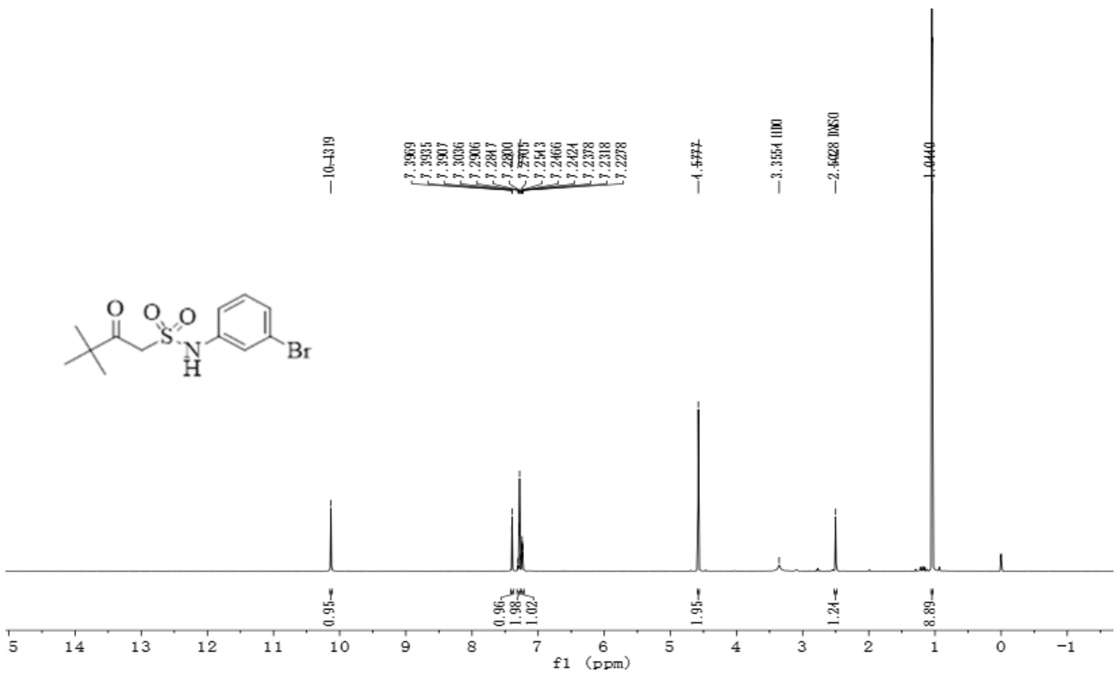

Figure S25. ^{1}H NMR of compound *P-13*

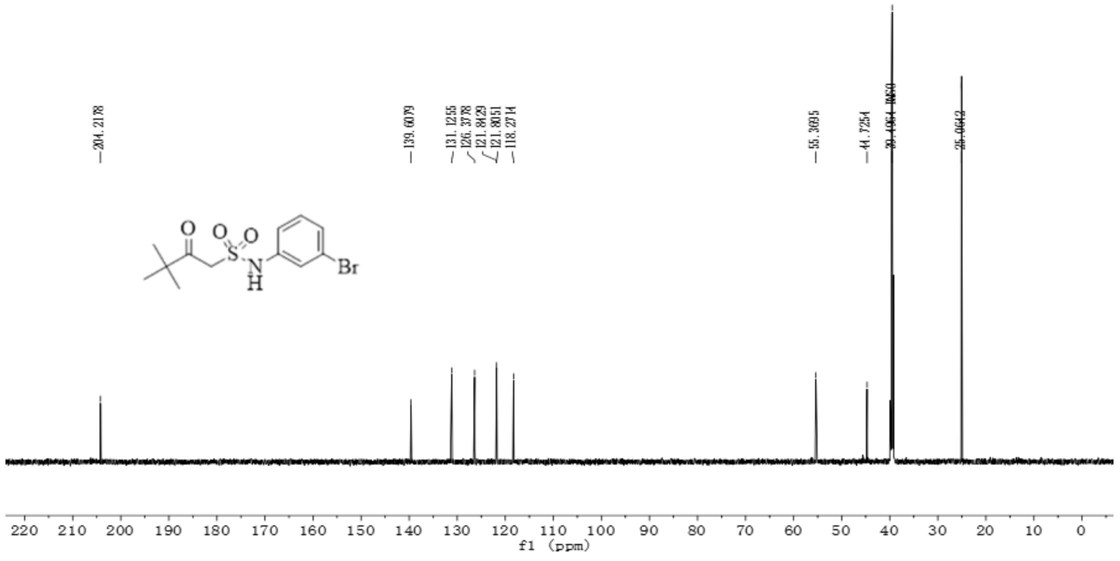

Figure S26. ^{13}C NMR of compound *P-13*

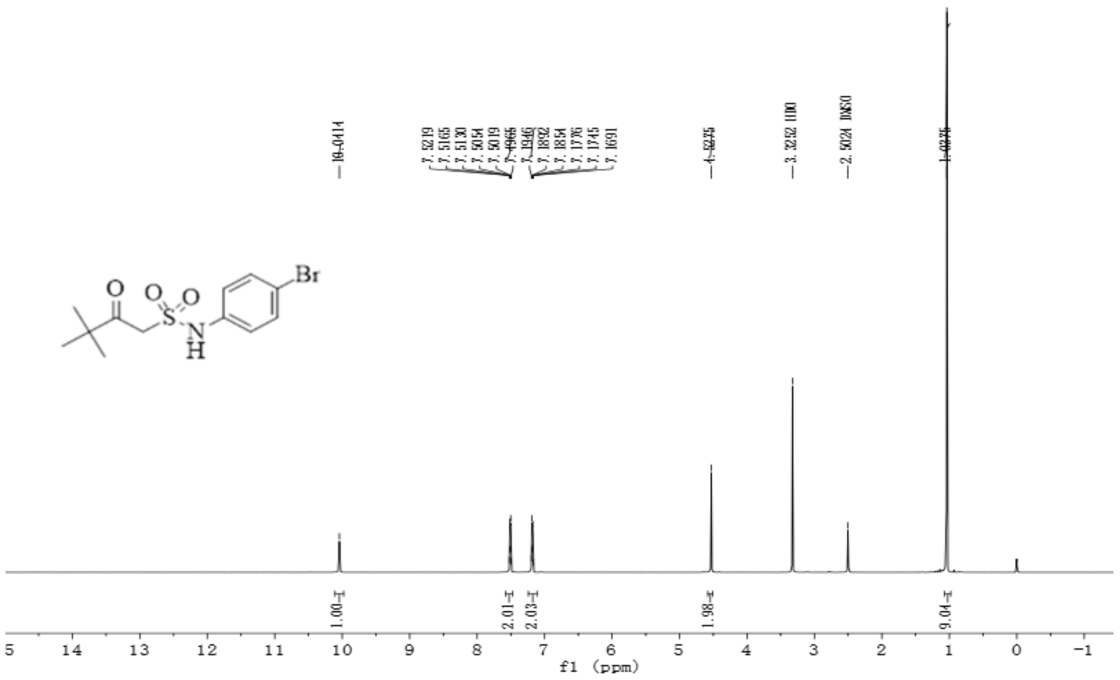

Figure S27. ^{1}H NMR of compound *P-14*

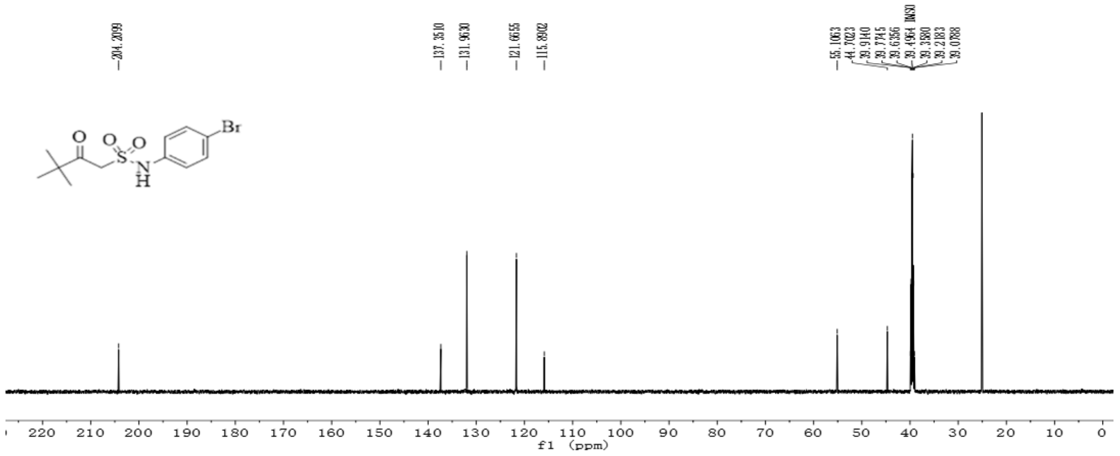

Figure S28. ^{13}C NMR of compound *P-14*

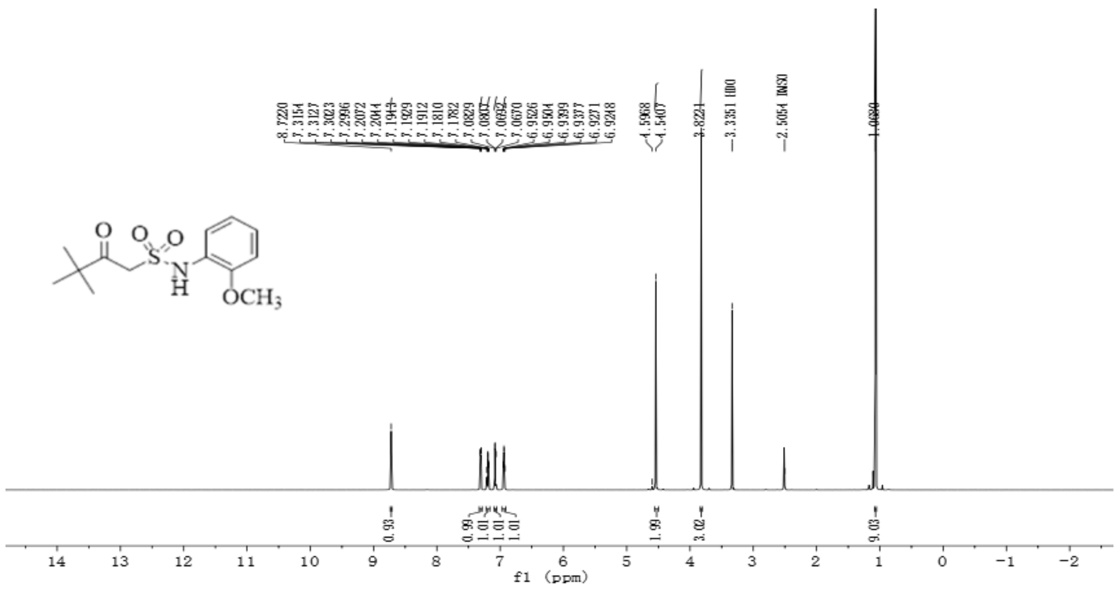

Figure S29. ^{1}H NMR of compound *P-15*

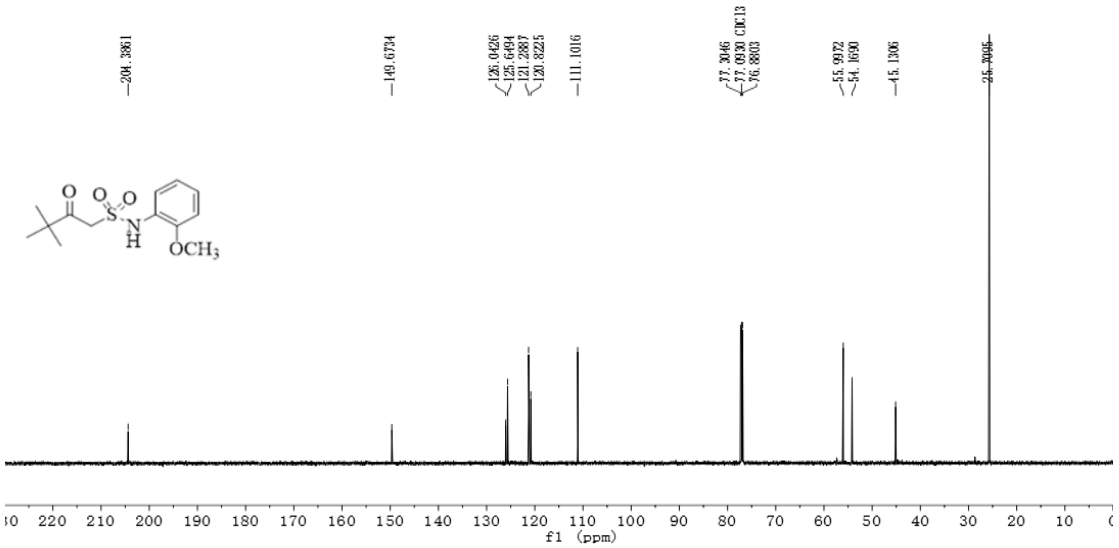

Figure S30. ^{13}C NMR of compound *P-15*

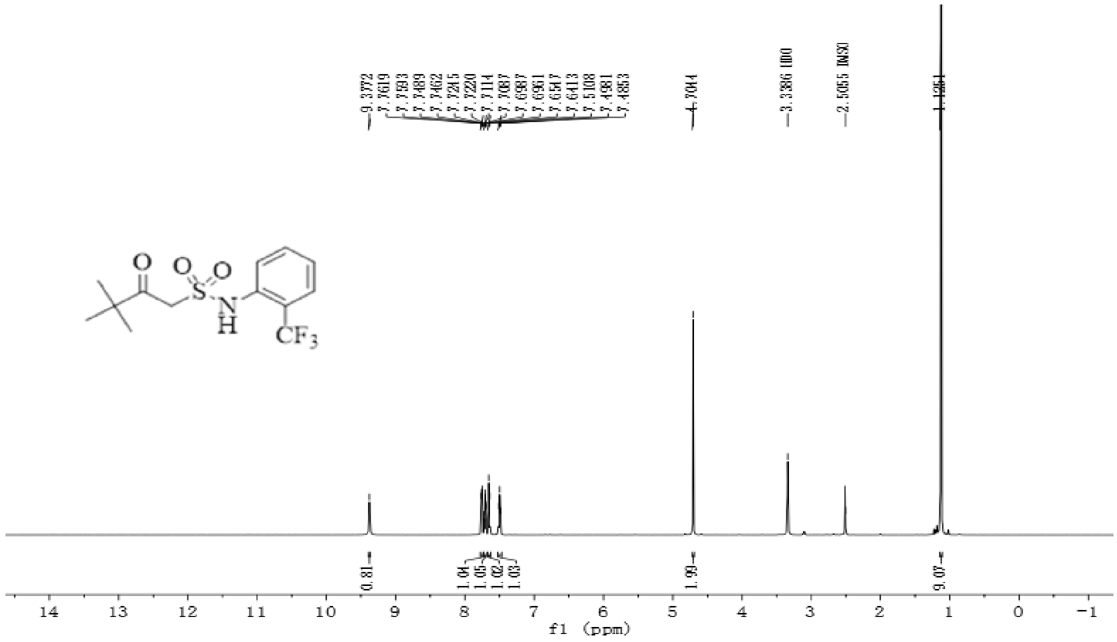

Figure S31. ^{1}H NMR of compound *P-16*

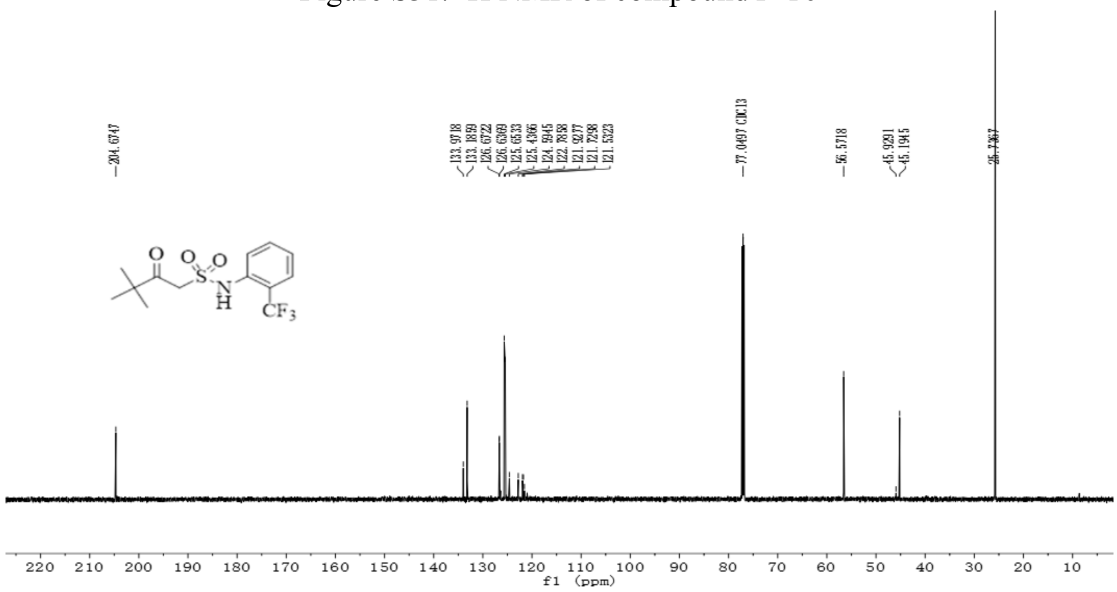

Figure S32. ^{13}C NMR of compound *P-16*

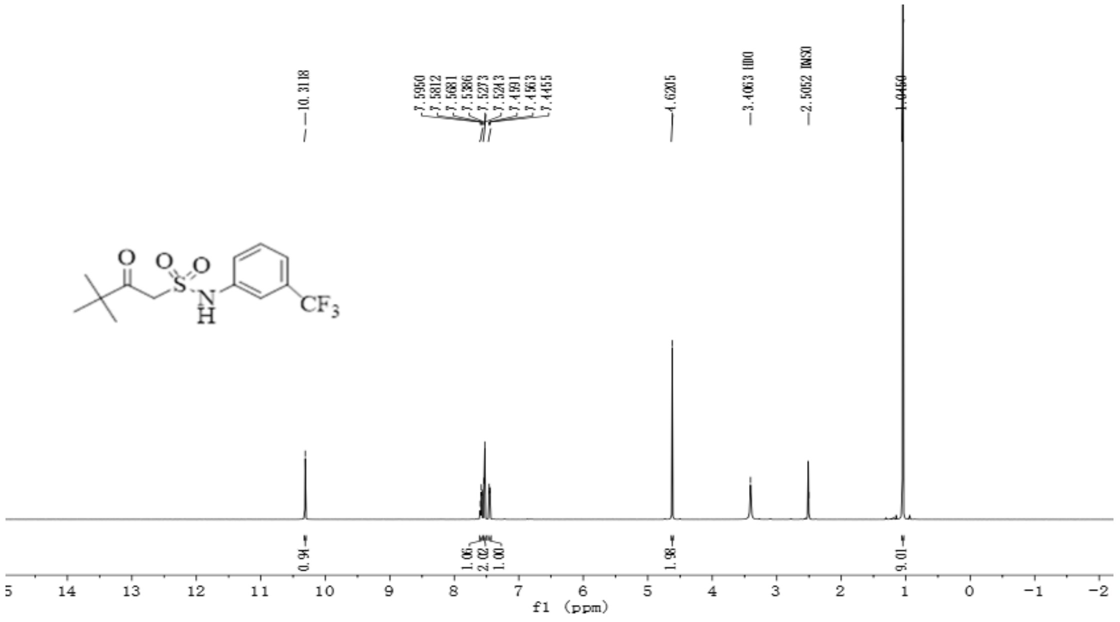

Figure S33. ^{1}H NMR of compound *P-17*

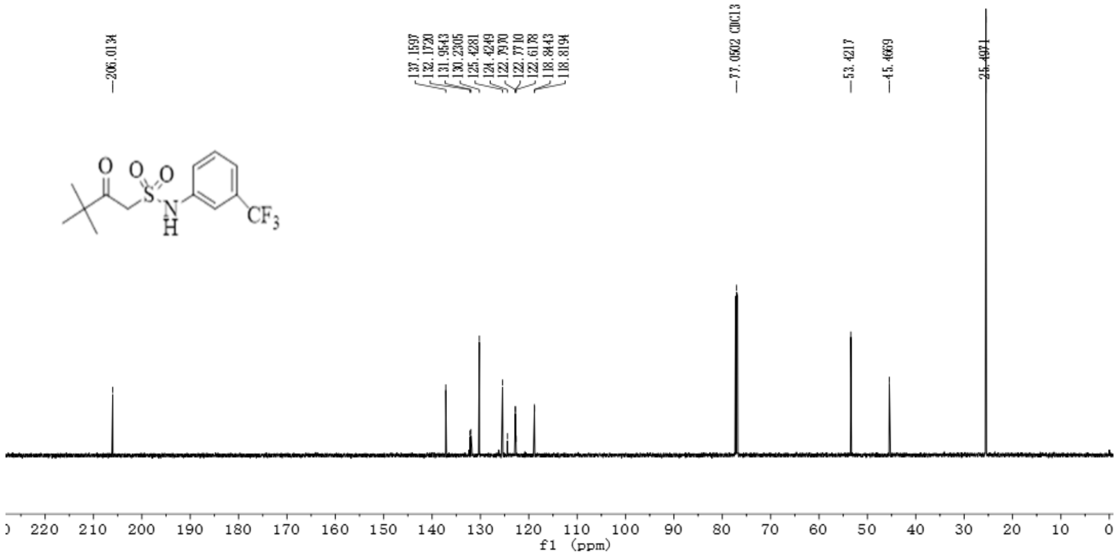

Figure S34. ^{13}C NMR of compound *P-17*

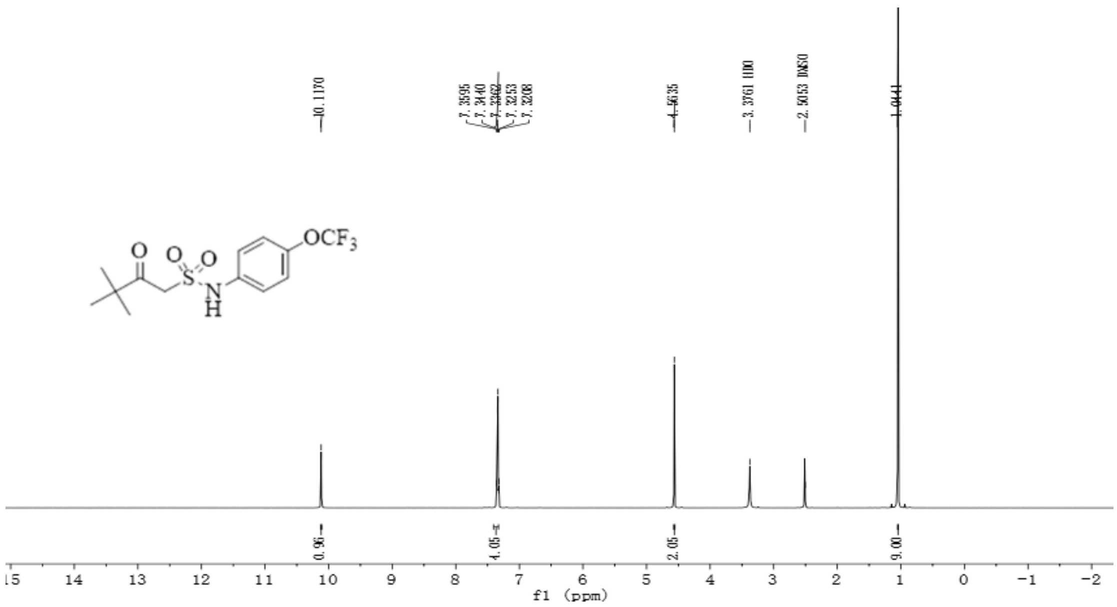

Figure S35. ^{1}H NMR of compound *P-18*

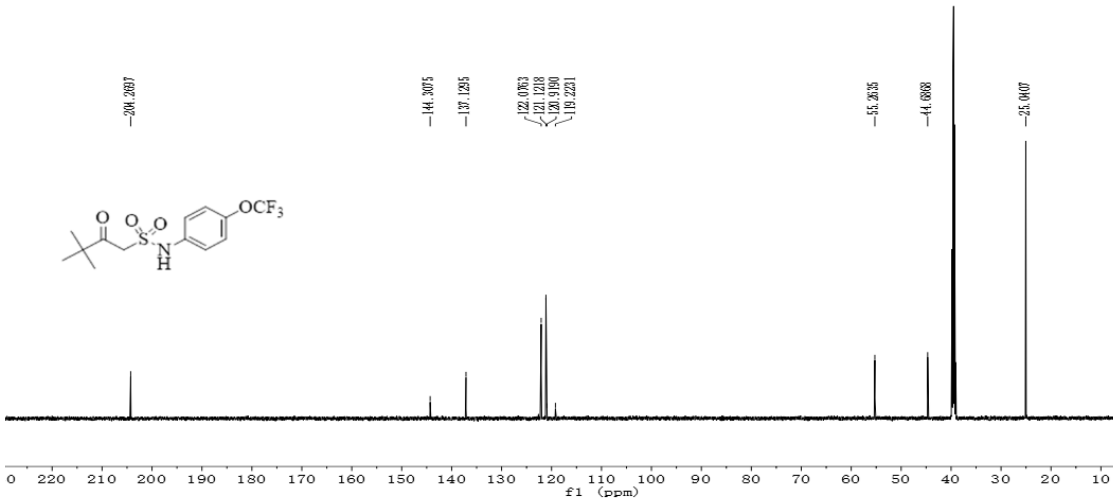

Figure S36. ^{13}C NMR of compound *P-18*

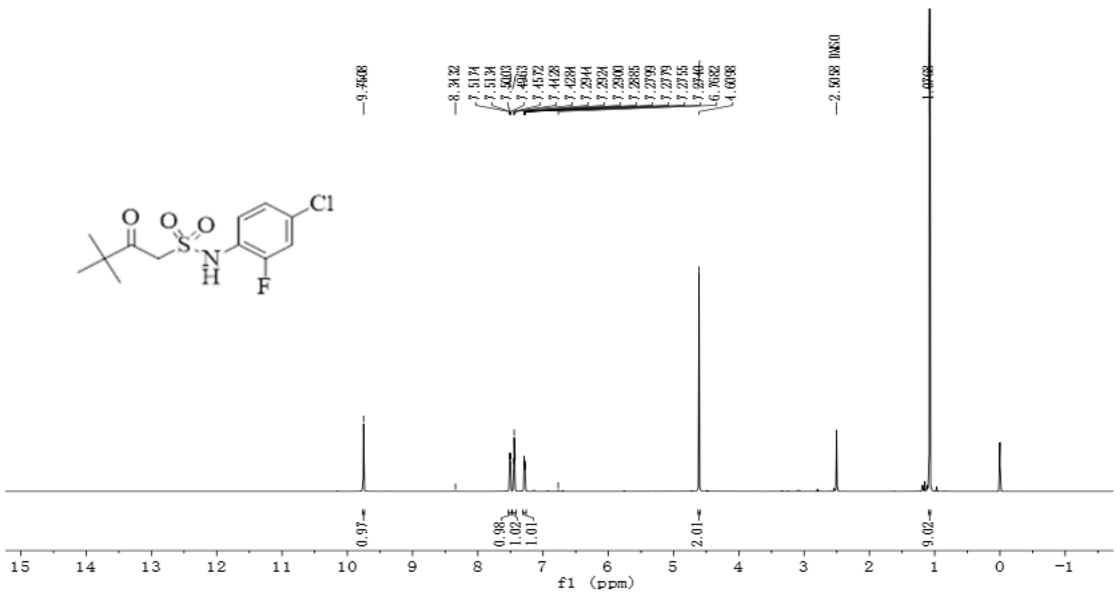

Figure S37. ^{1}H NMR of compound *P-19*

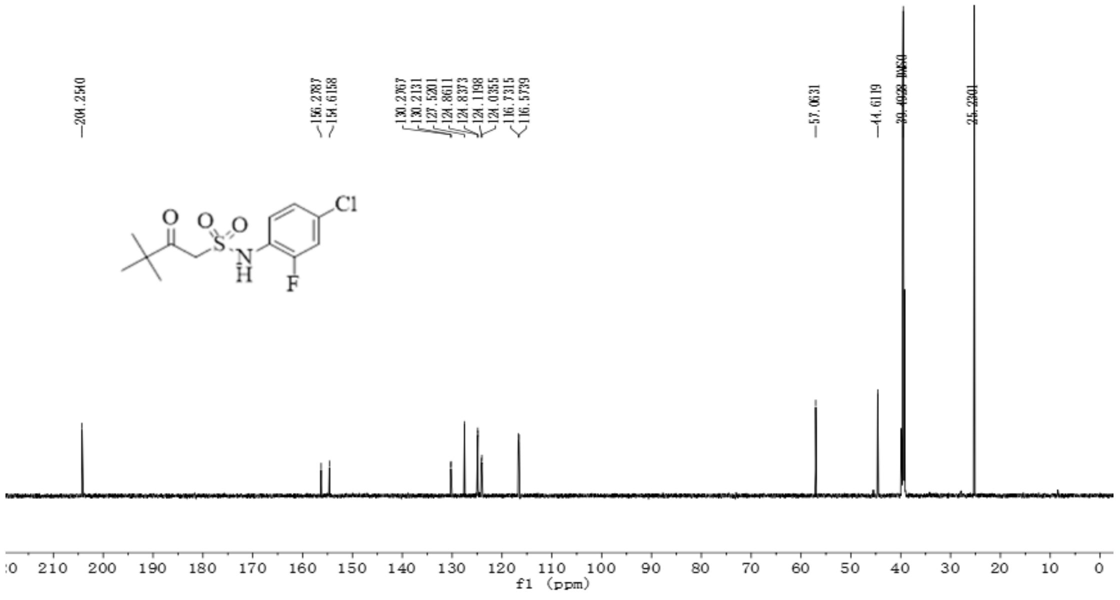

Figure S38. ^{13}C NMR of compound *P-19*

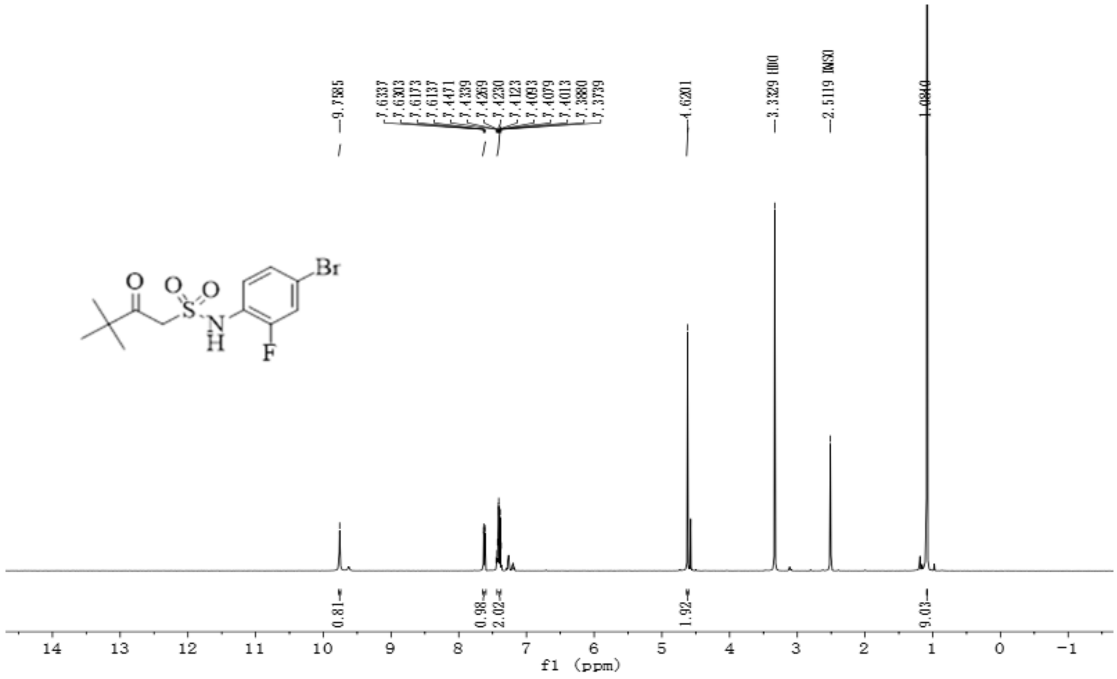

Figure S39. ^{1}H NMR of compound *P-20*

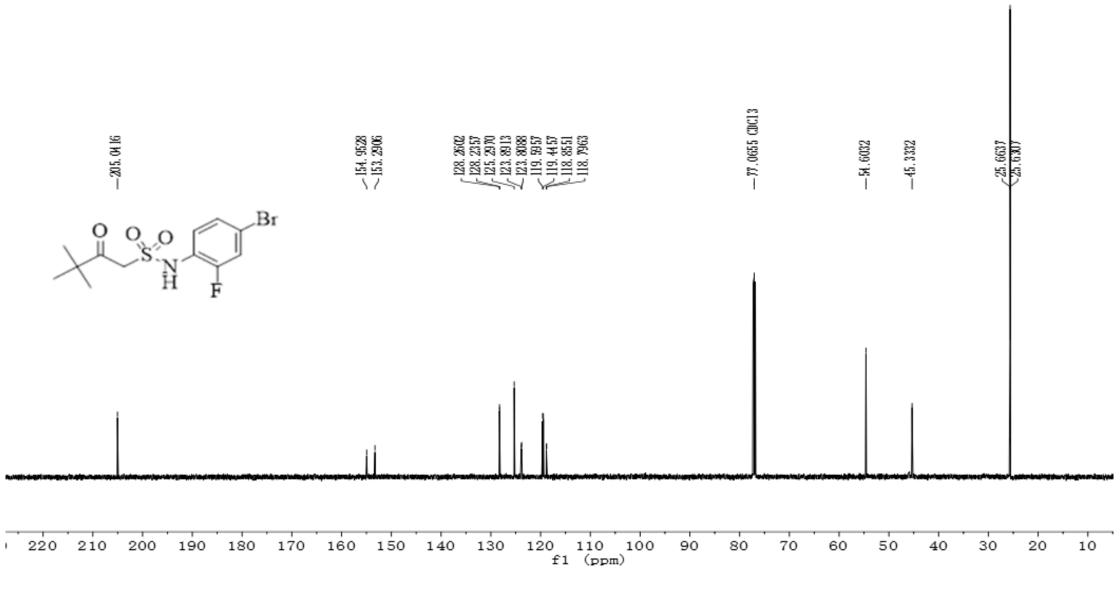

Figure S40. ^{13}C NMR of compound *P-20*

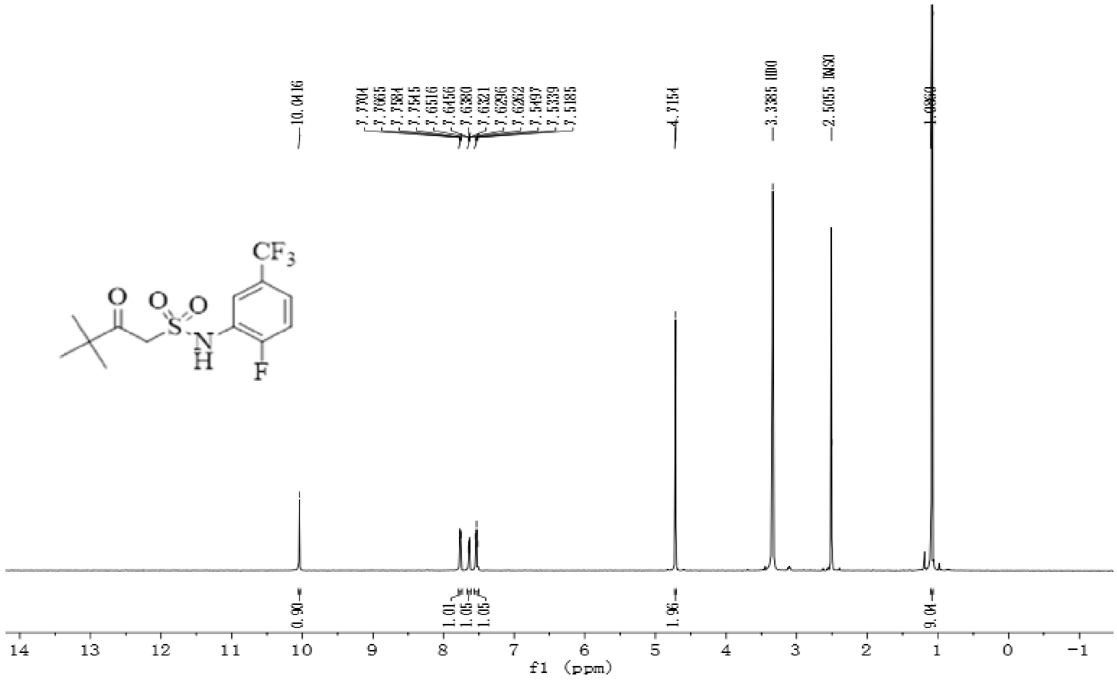

Figure S41. ^{1}H NMR of compound *P-21*

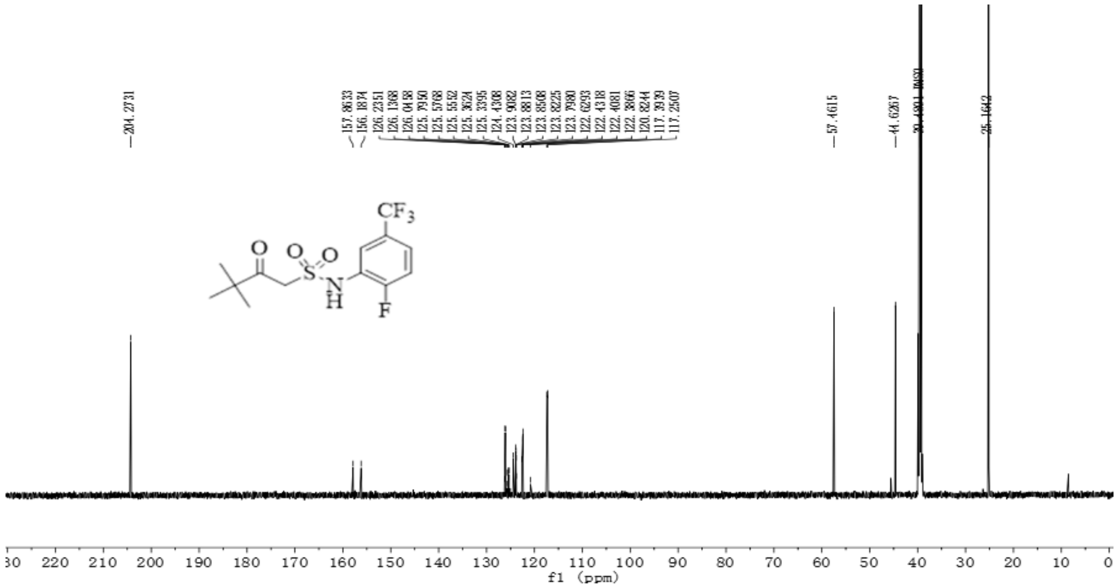

Figure S42. ^{13}C NMR of compound *P-21*

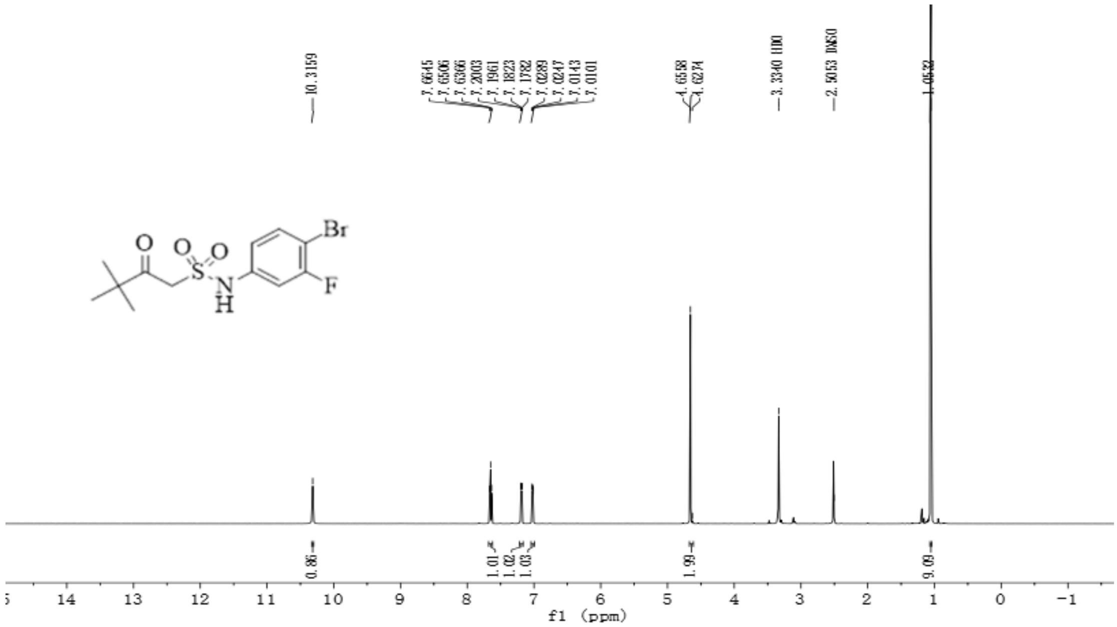

Figure S43. ^{1}H NMR of compound *P-22*

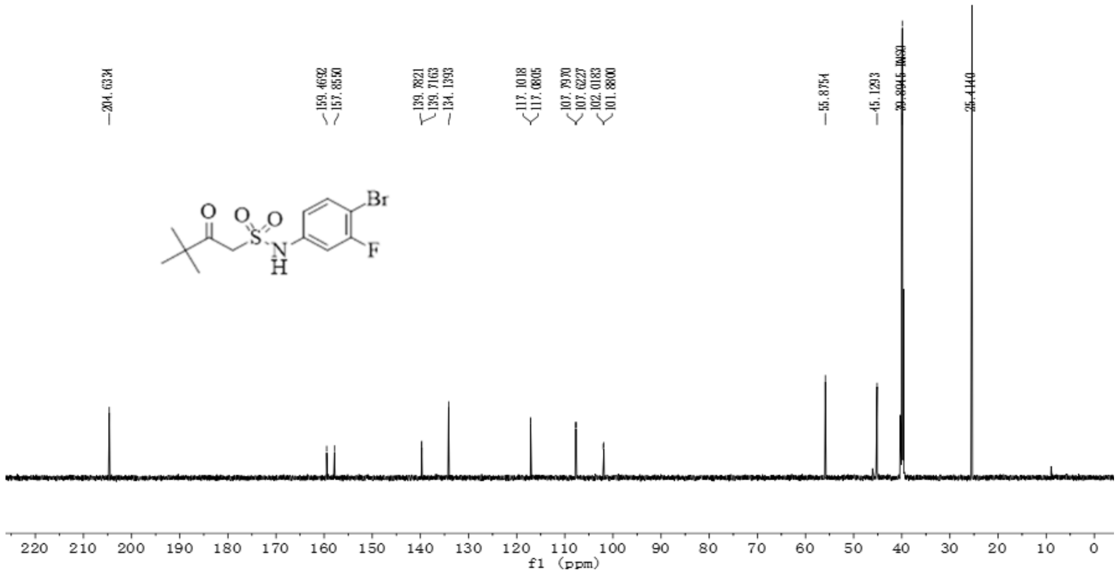

Figure S44. ^{13}C NMR of compound *P-22*

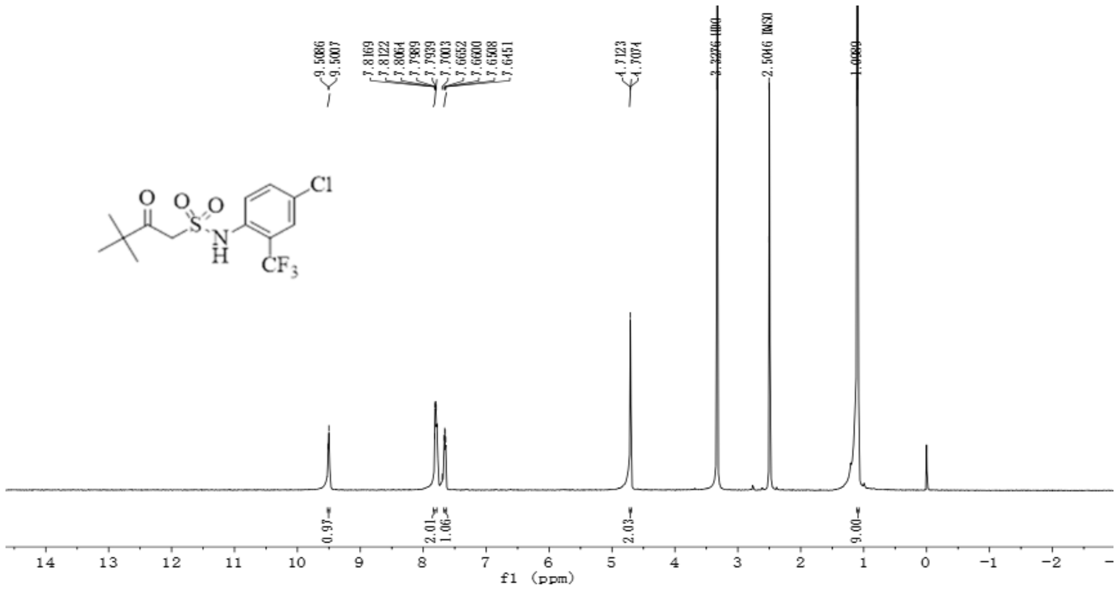

Figure S45. ^{1}H NMR of compound *P-23*

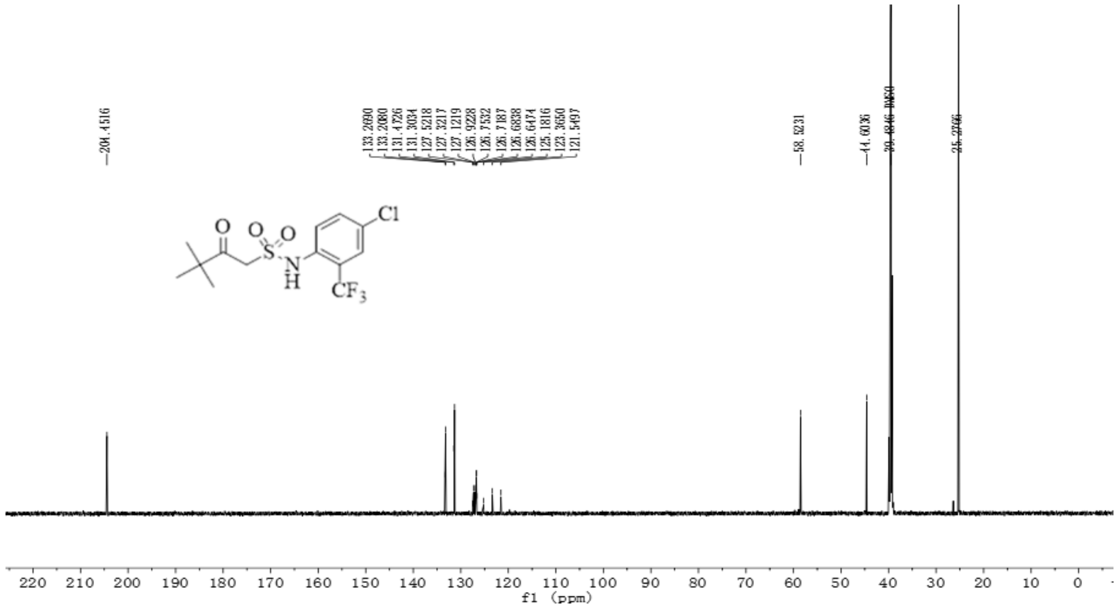

Figure S46. ^{13}C NMR of compound *P-23*

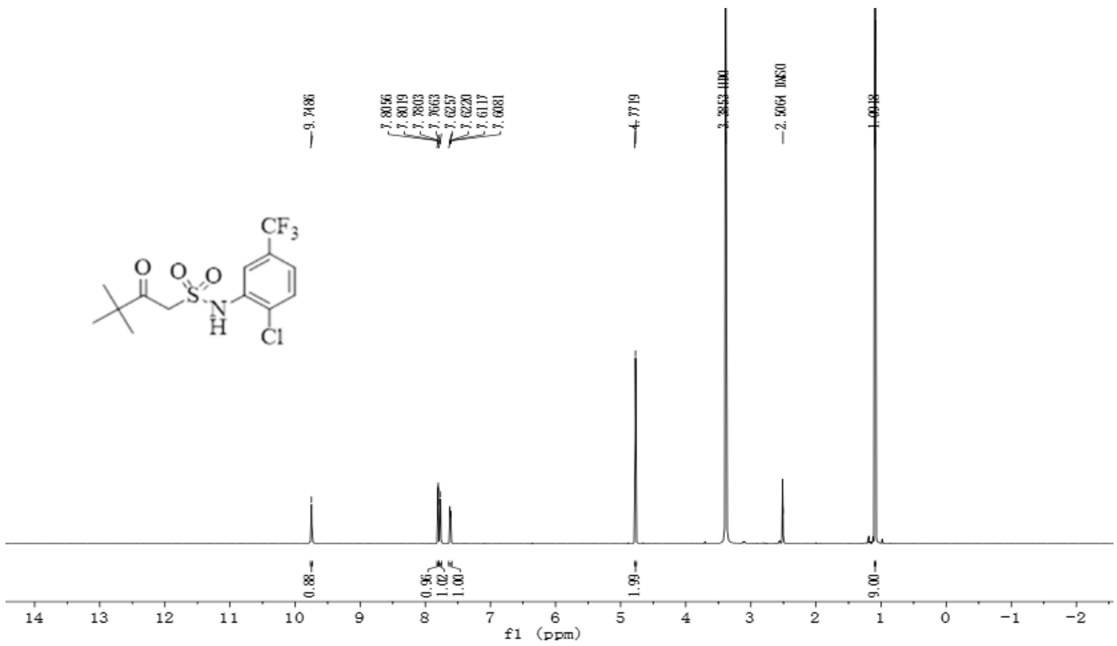

Figure S47. ^{1}H NMR of compound *P-24*

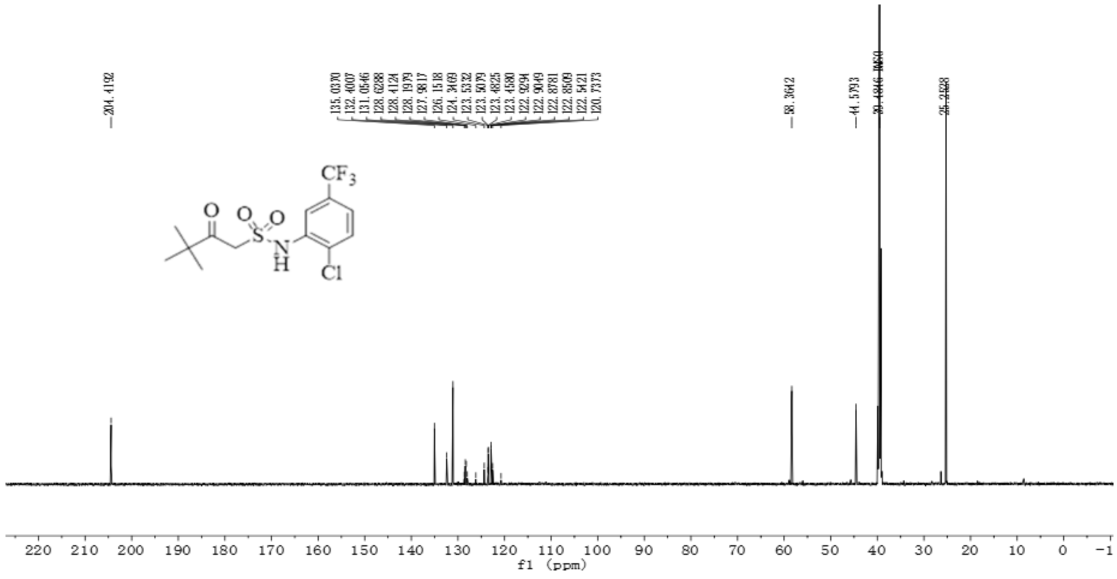

Figure S48. ^{13}C NMR of compound *P-24*

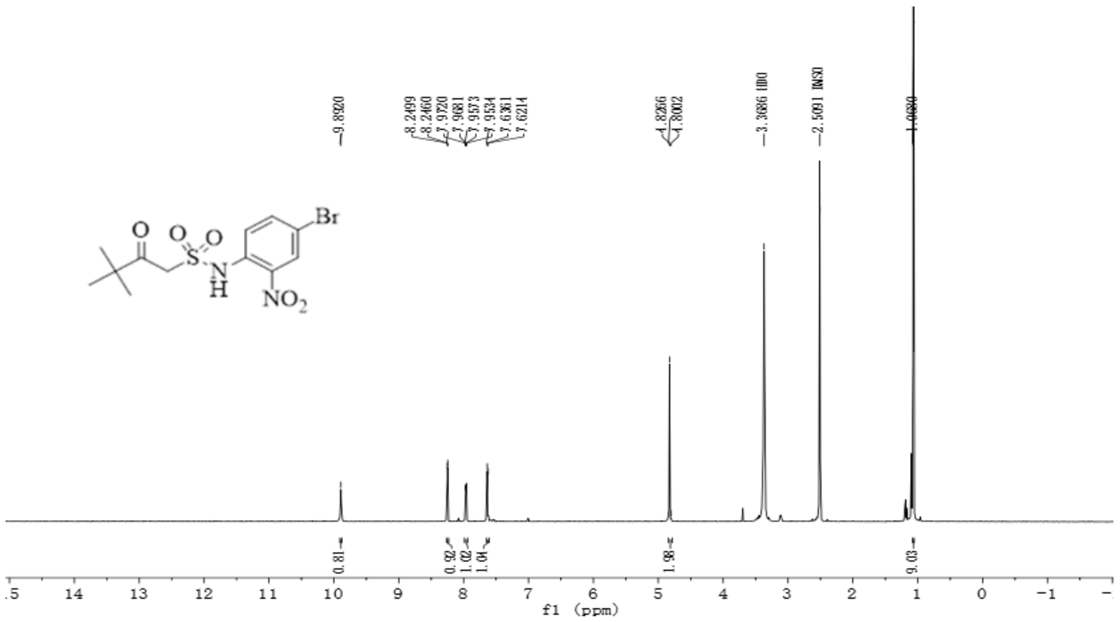

Figure S49. ^{1}H NMR of compound *P-25*

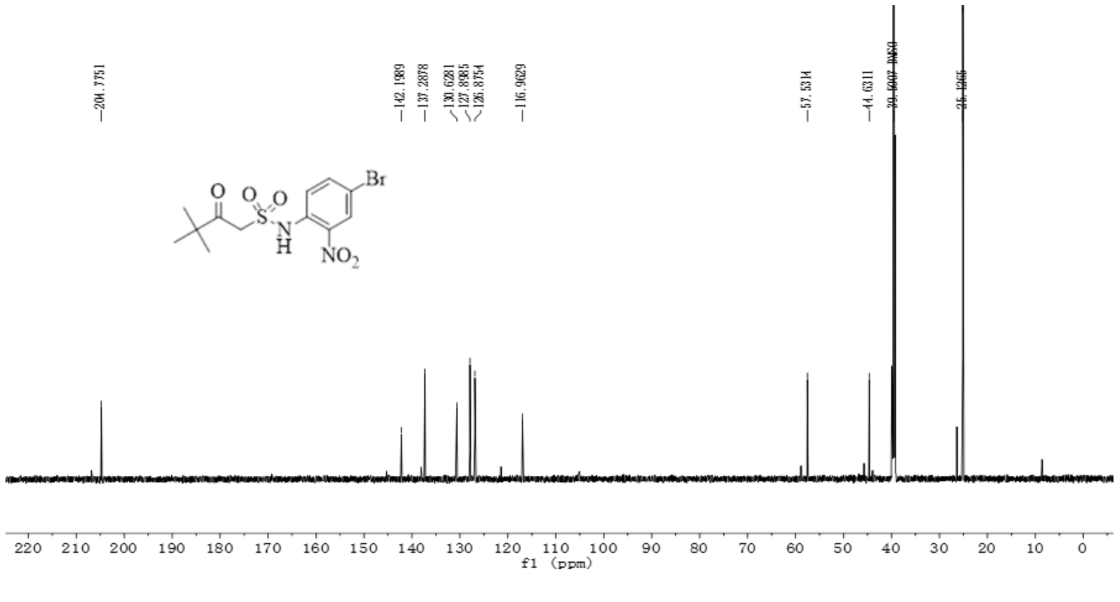

Figure S50. ^{13}C NMR of compound *P-25*

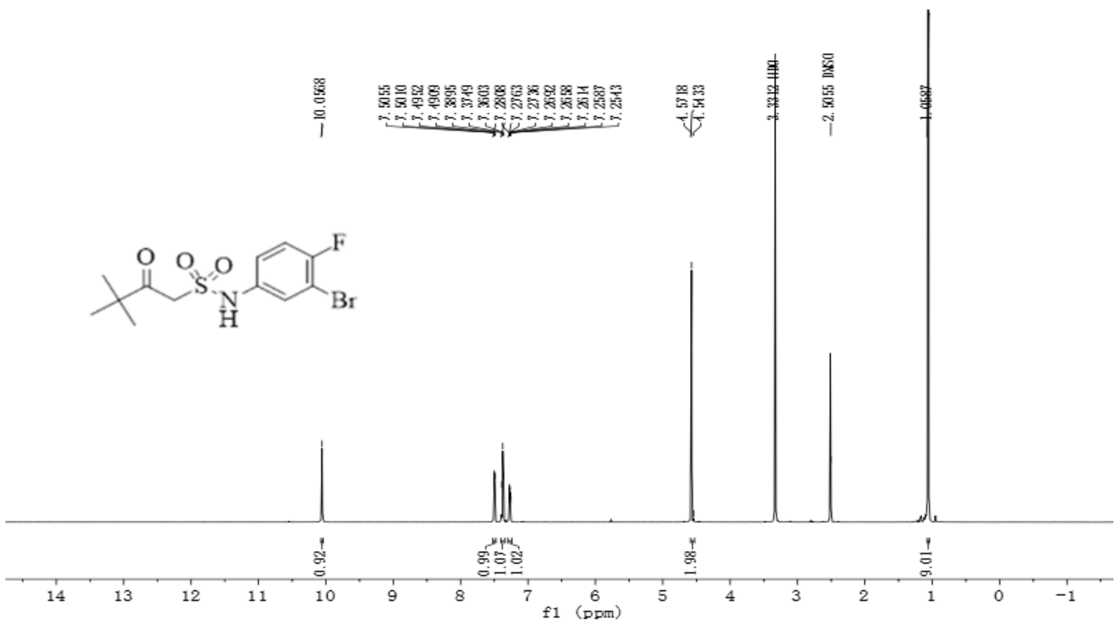

Figure S51. ^{1}H NMR of compound *P-26*

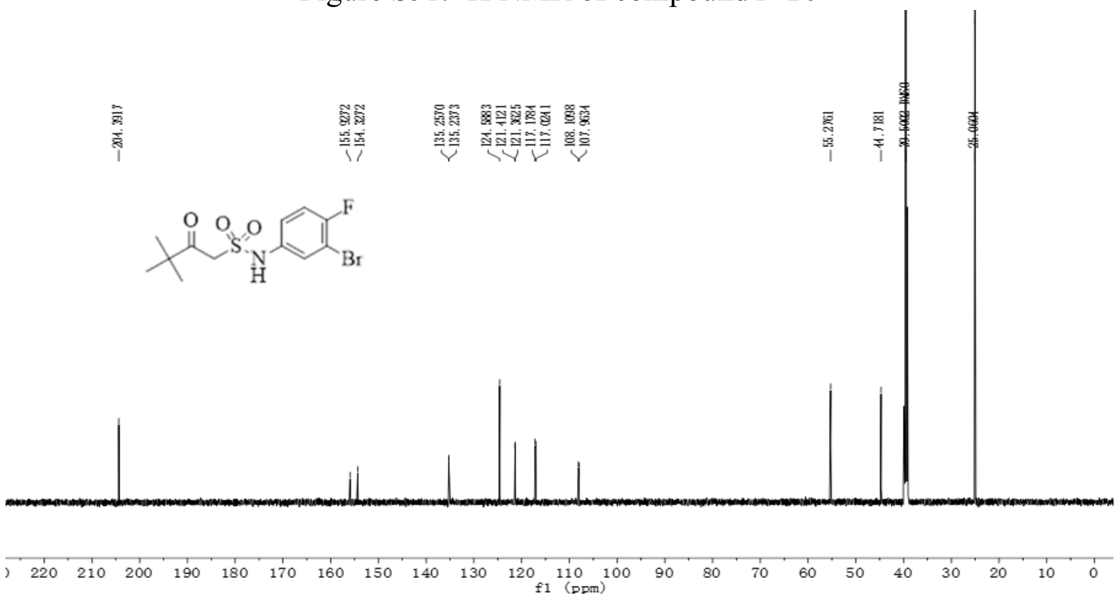

Figure S52. ^{13}C NMR of compound *P-26*

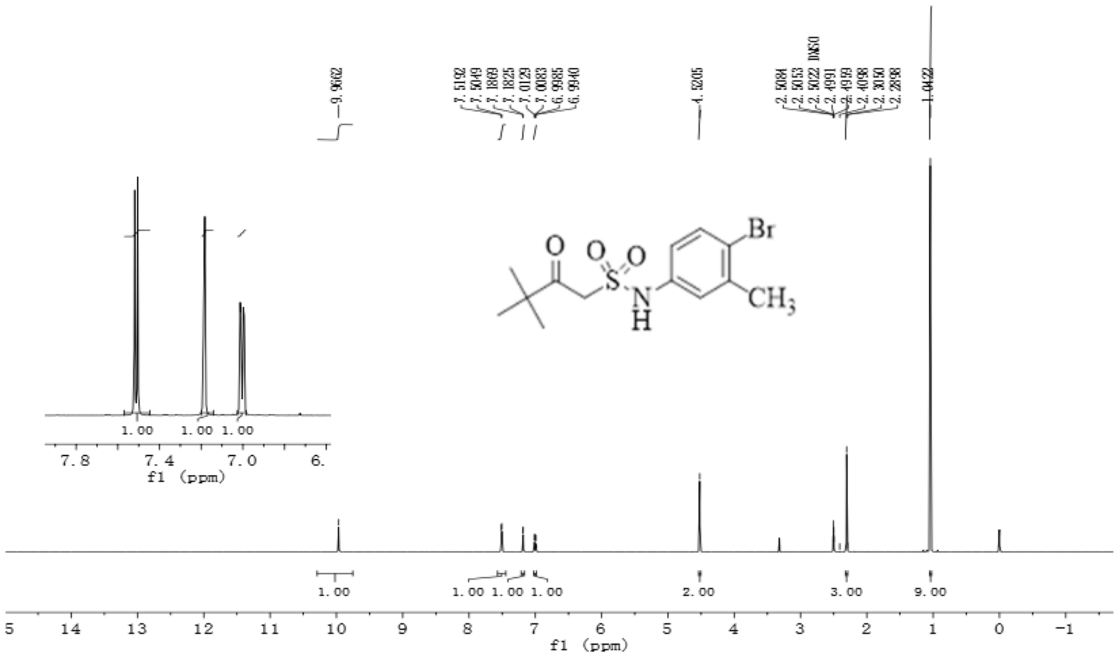

Figure S53. ^{1}H NMR of compound *P-27*

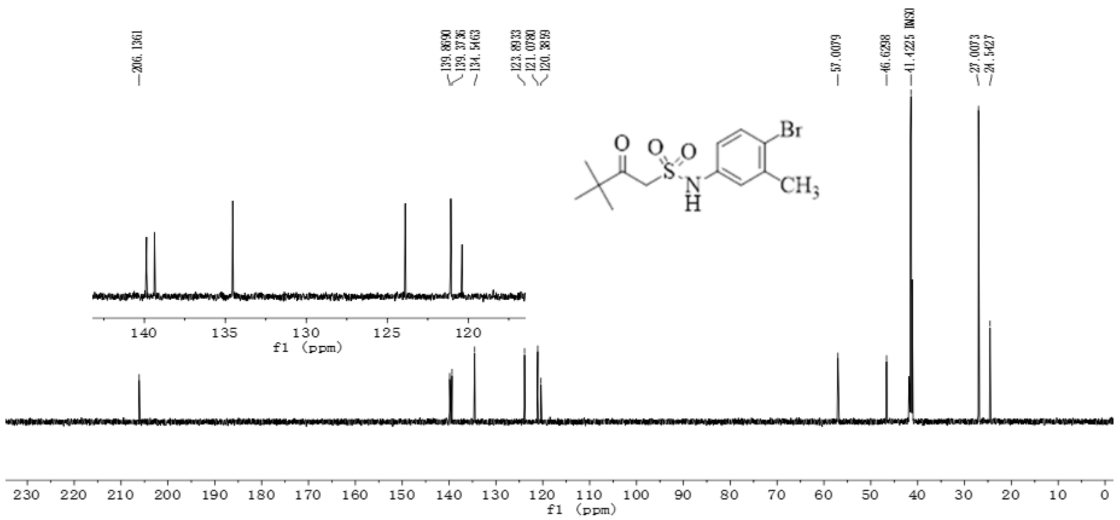

Figure S54. ^{13}C NMR of compound *P-27*

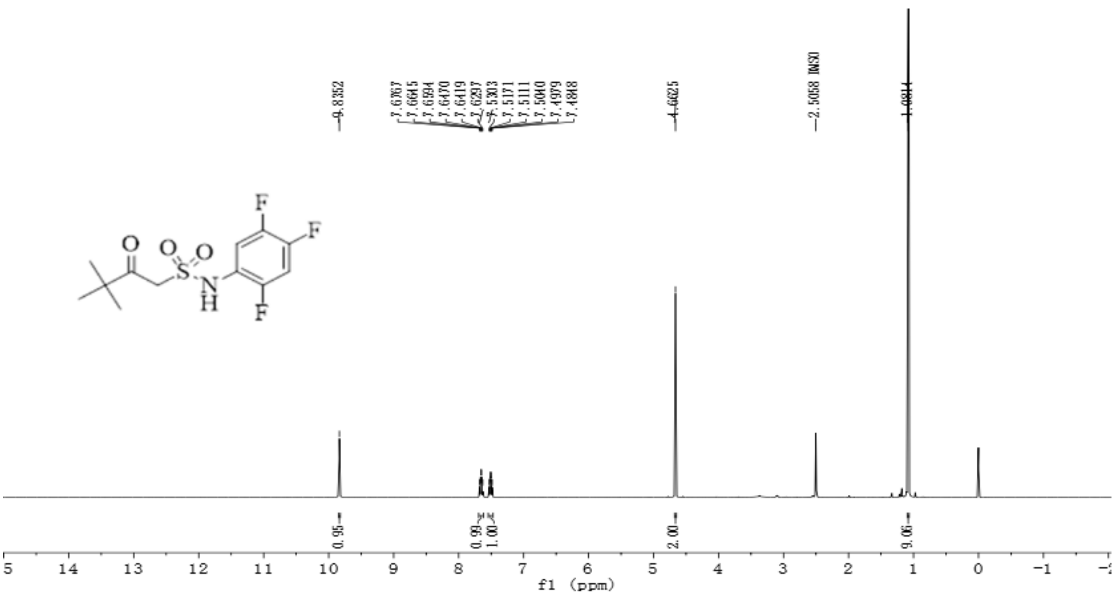

Figure S55. ^{1}H NMR of compound *P*-*28*

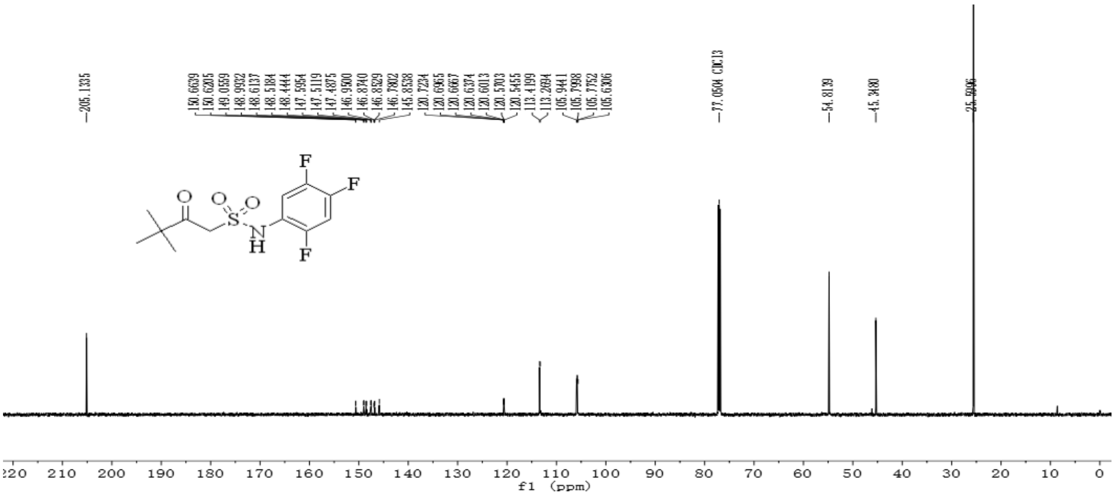

Figure S56. ^{13}C NMR of compound *P*-*28*

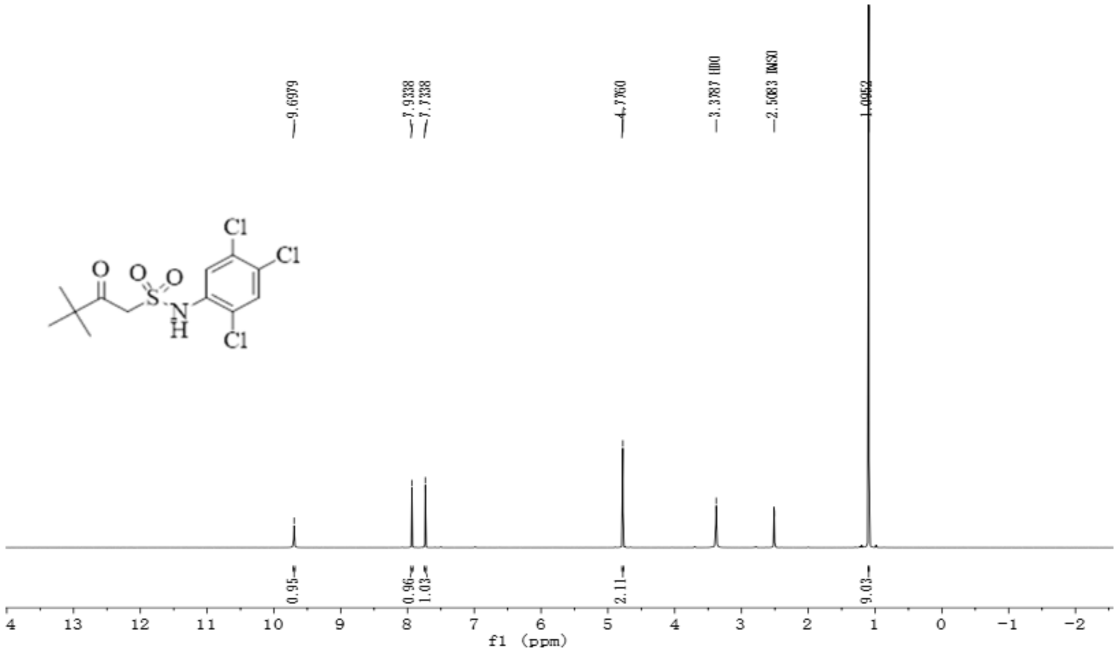

Figure S57. ^{1}H NMR of compound *P-29*

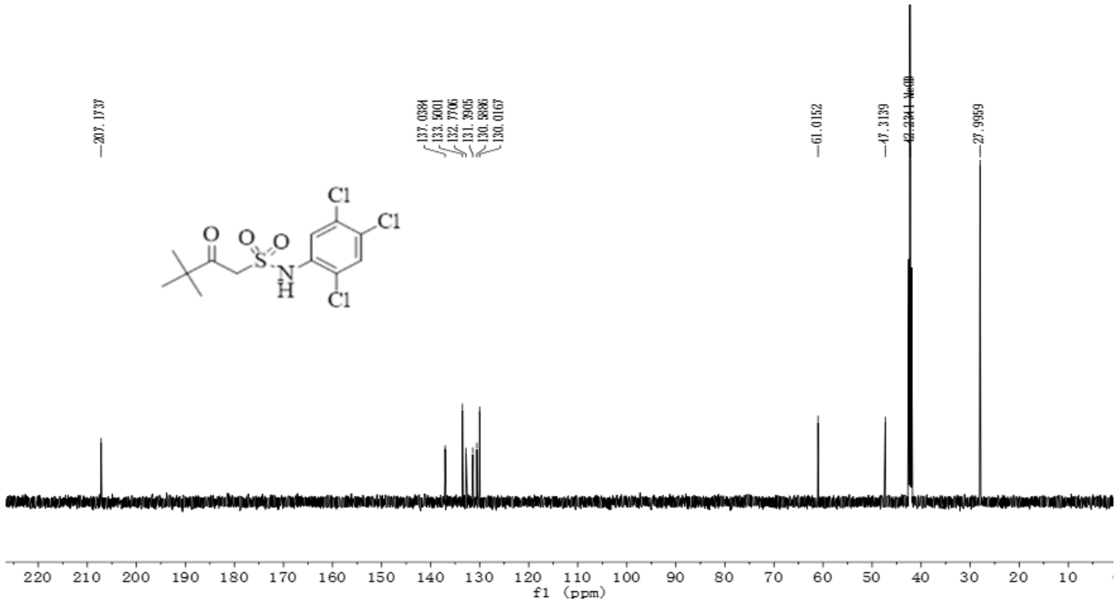

Figure S58. ^{13}C NMR of compound *P-29*

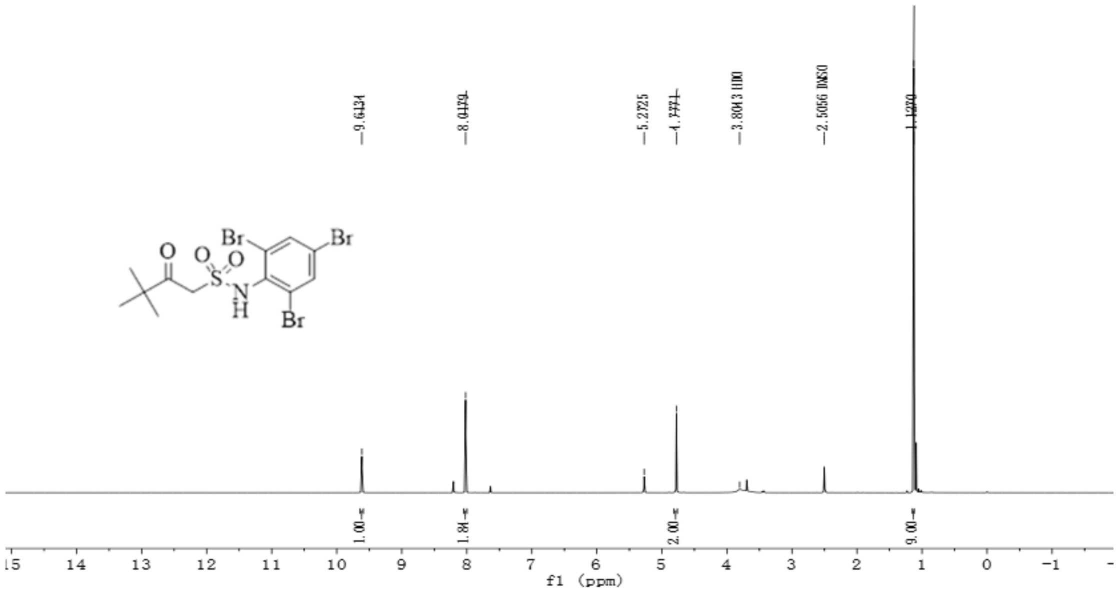

Figure S59. ^{1}H NMR of compound *P-30*

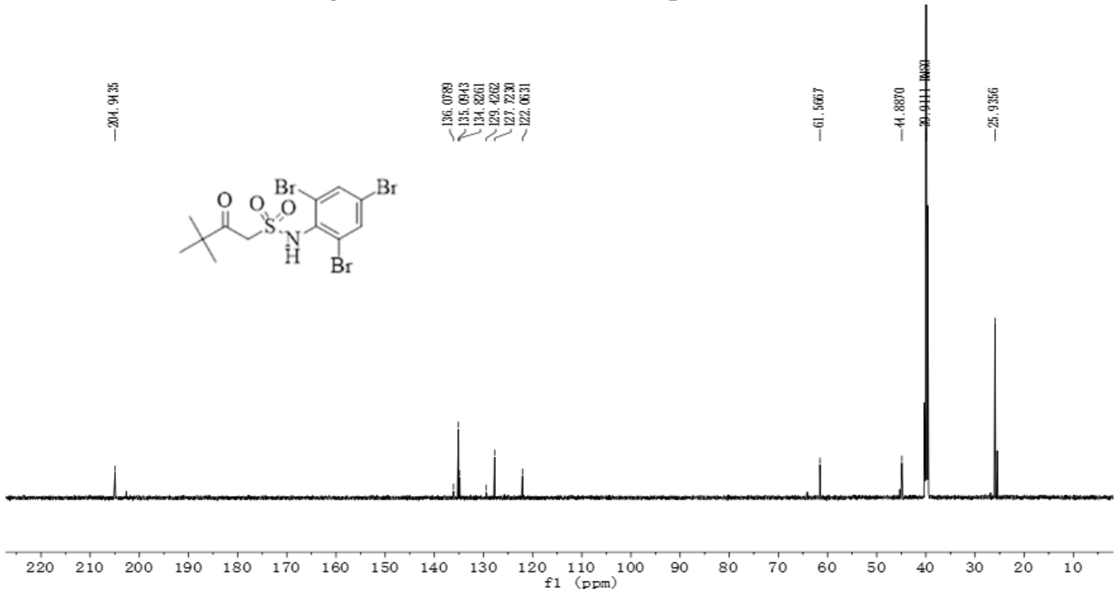

Figure S60. ^{13}C NMR of compound *P-30*

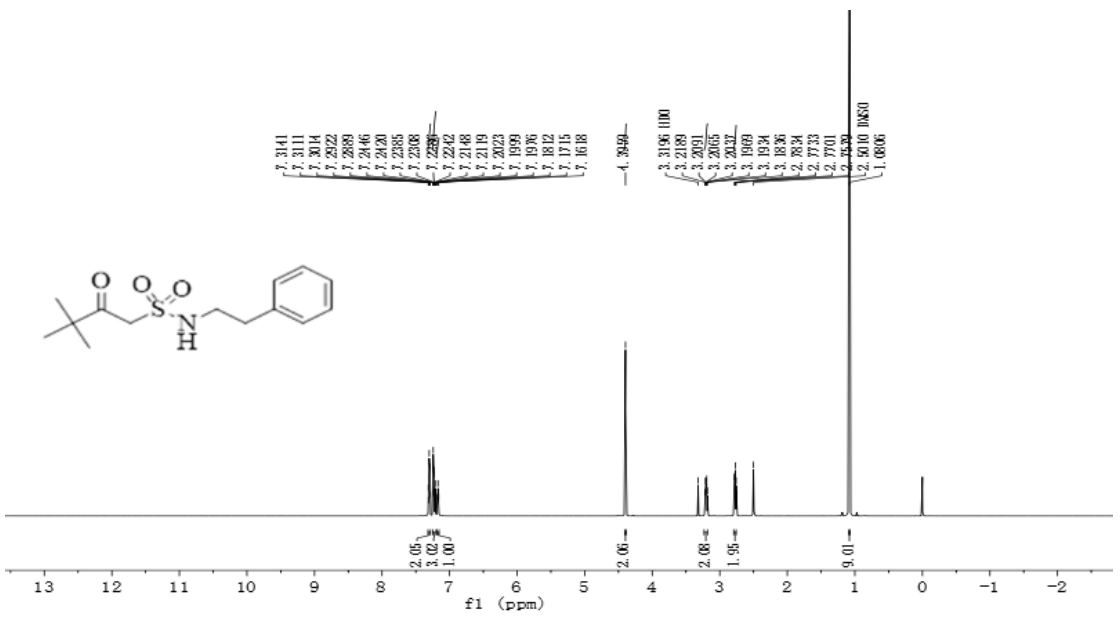

Figure S61. ^{1}H NMR of compound *P-31*

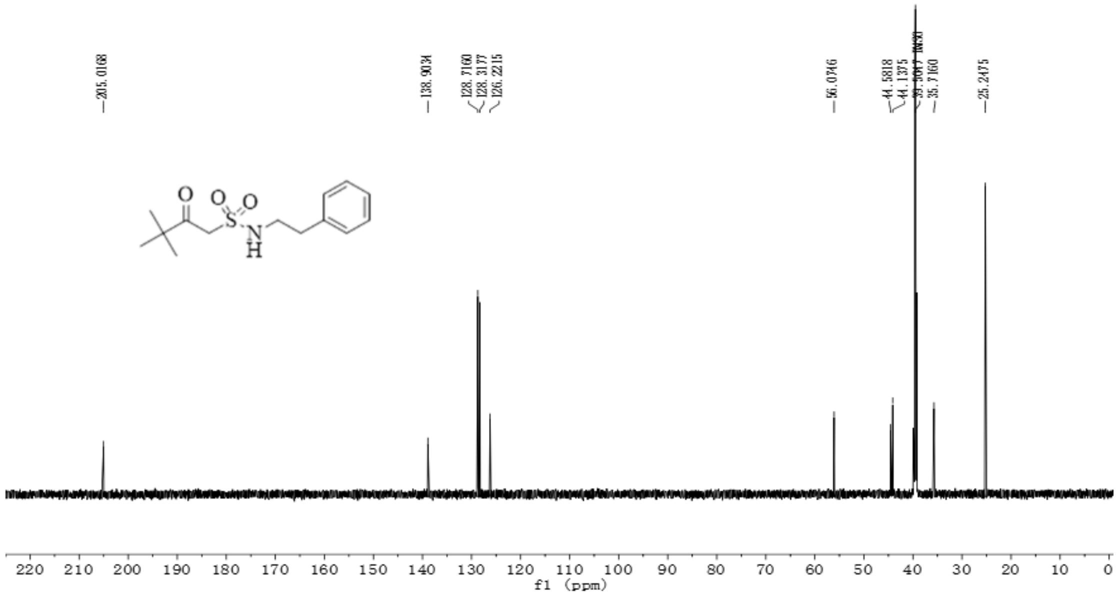

Figure S62. ^{13}C NMR of compound *P-31*

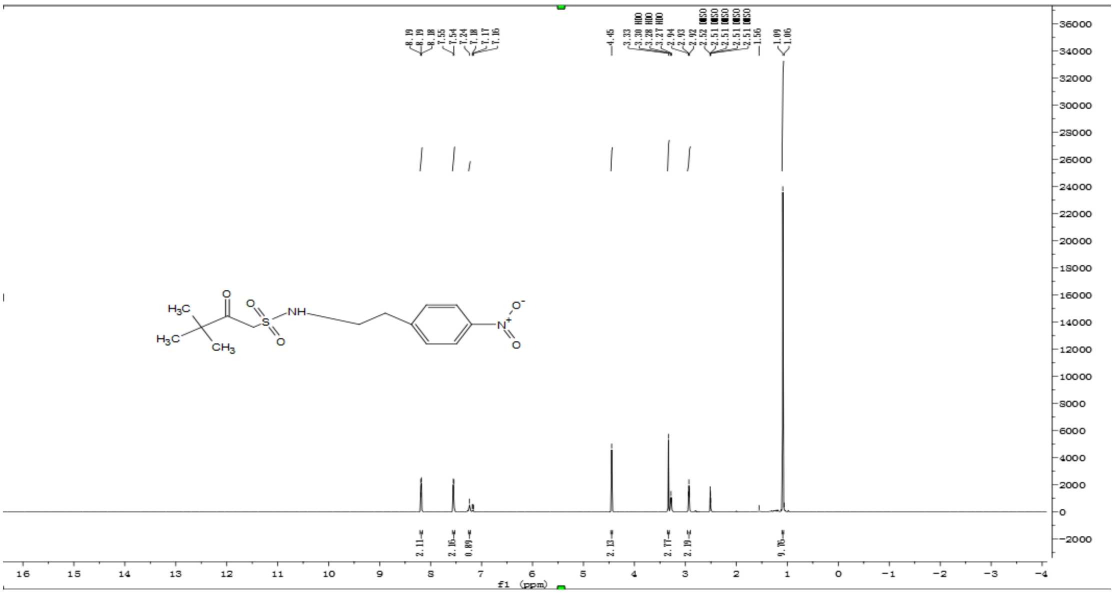

Figure S63. ^{1}H NMR of compound *P-32*

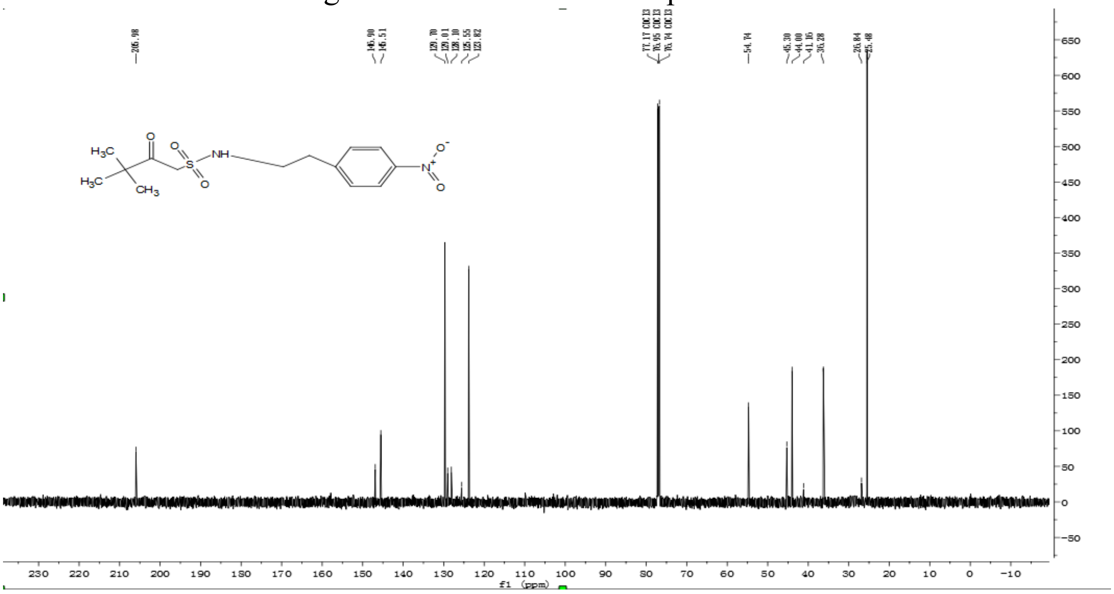

Figure S64. ^{13}C NMR of compound *P-32*

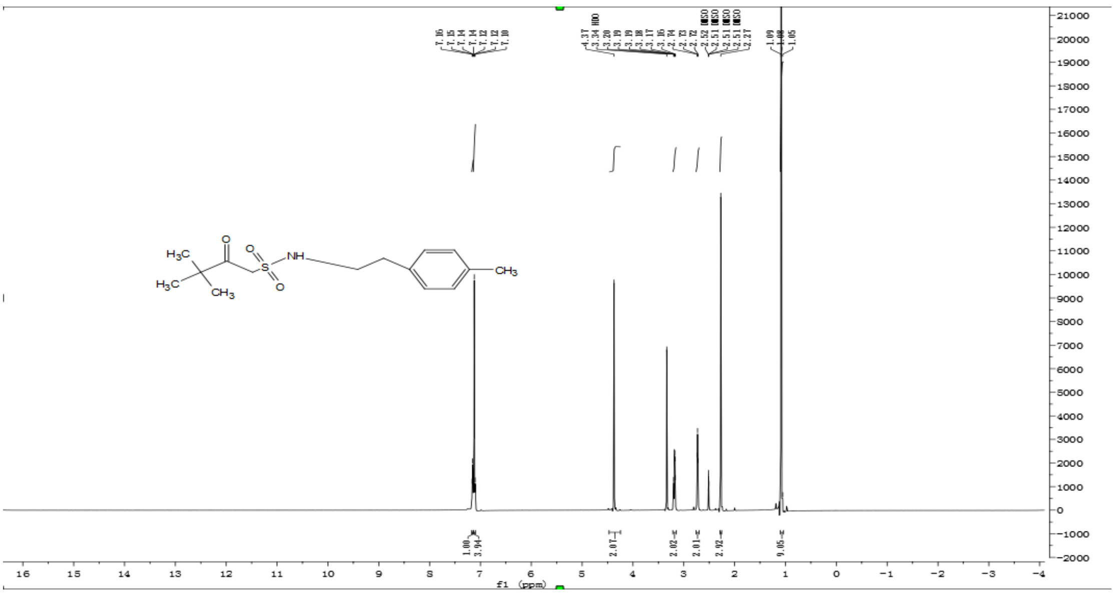

Figure S65. ^{1}H NMR of compound *P-33*

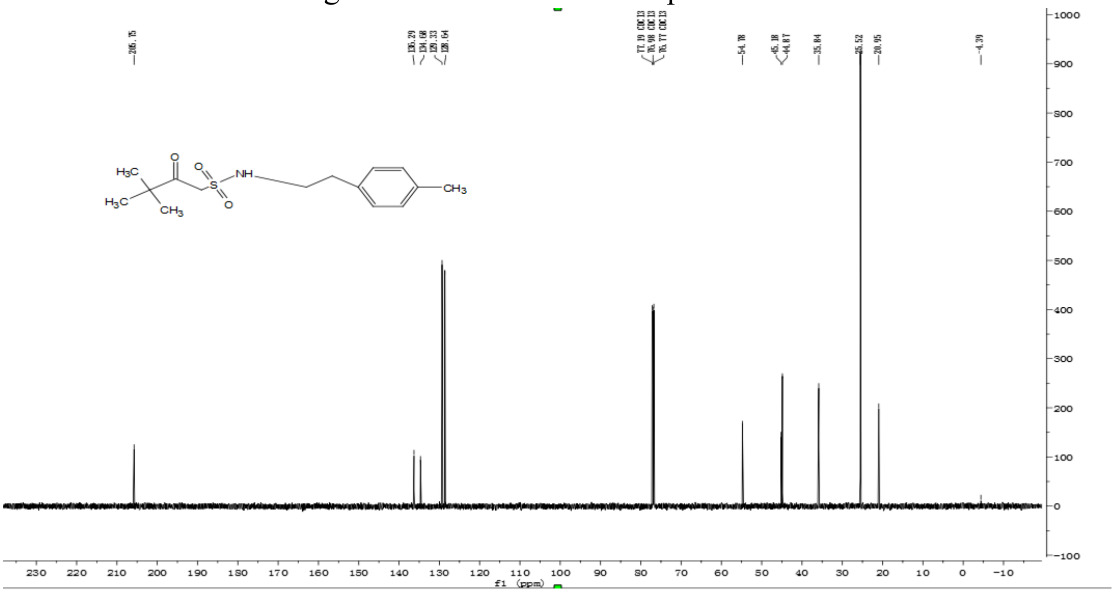

Figure S66. ^{13}C NMR of compound *P-33*

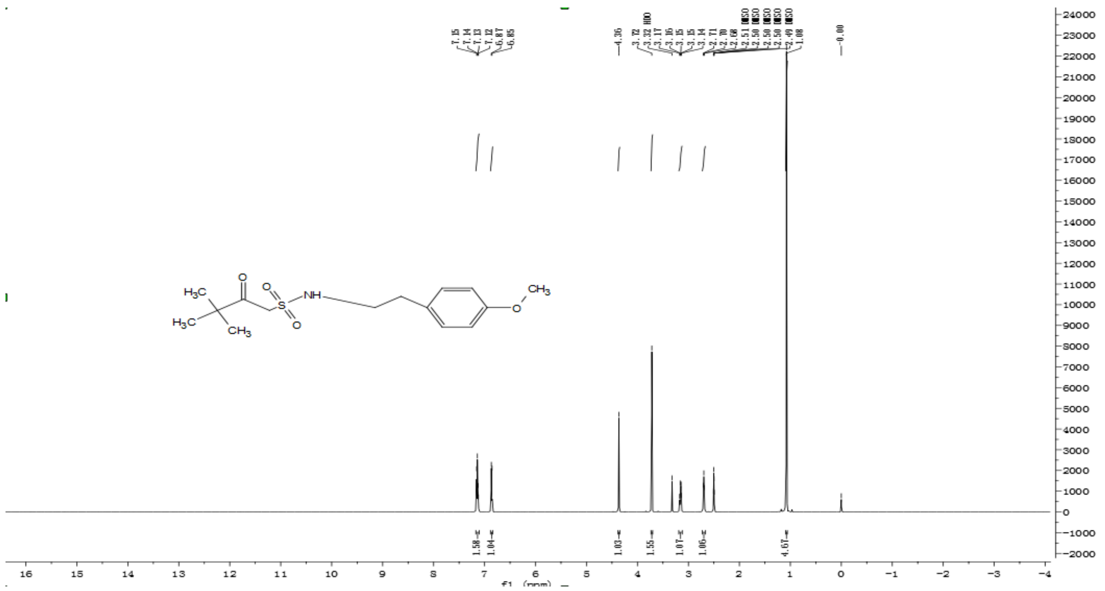

Figure S67. ^{1}H NMR of compound *P-34*

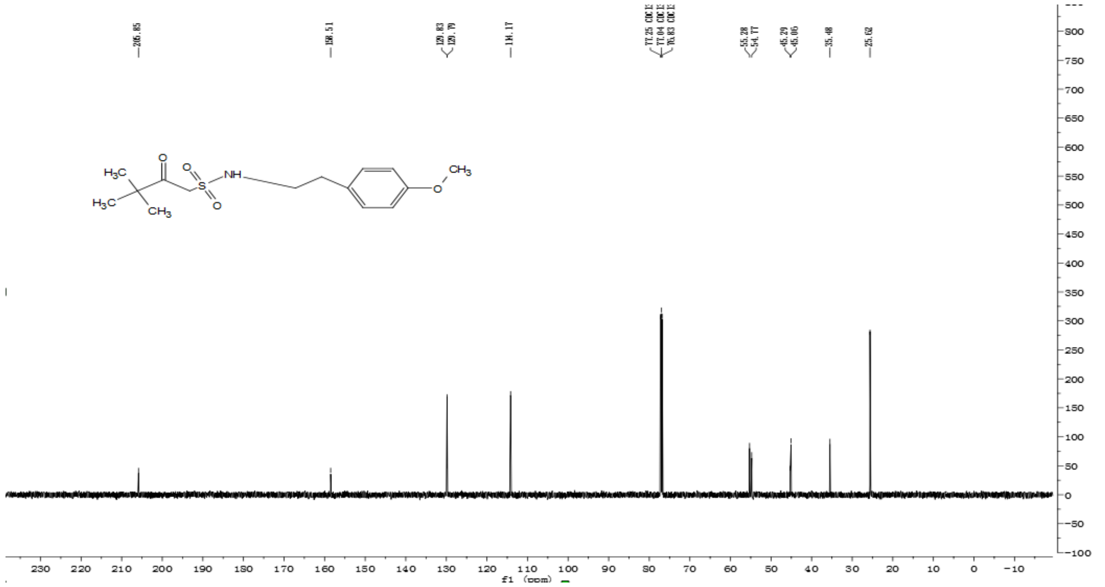

Figure S68. ^{13}C NMR of compound *P-34*

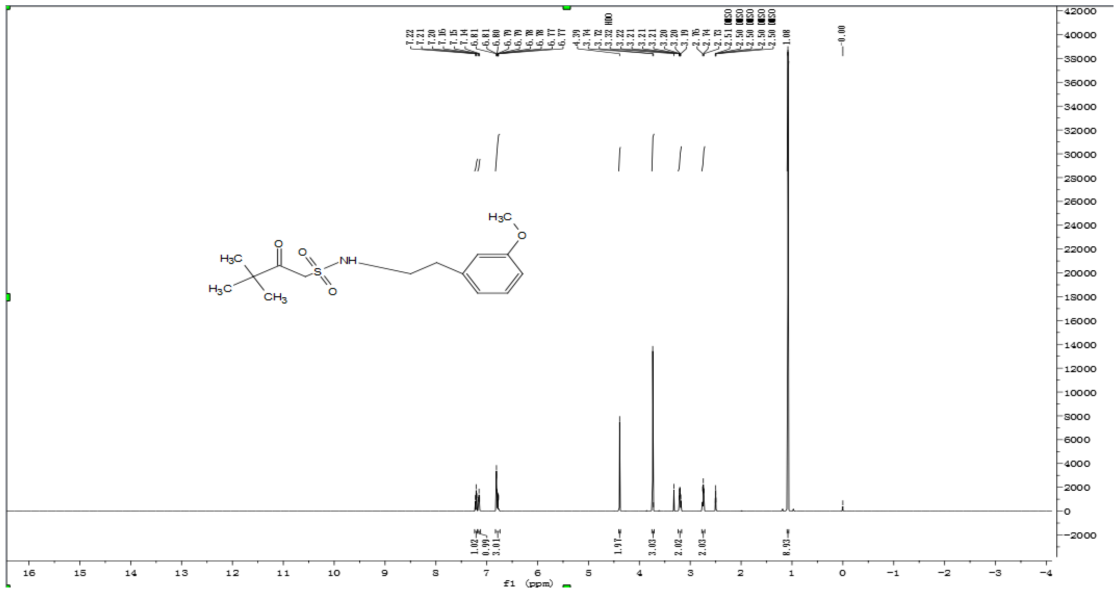

Figure S69. ^{1}H NMR of compound *P-35*

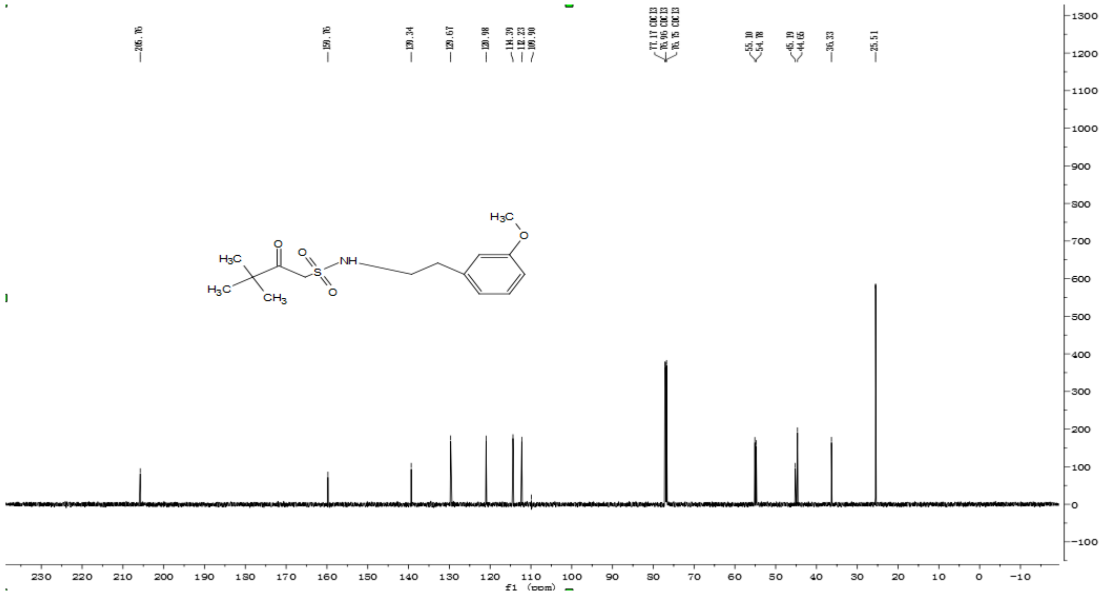

Figure S70. ^{13}C NMR of compound *P-35*

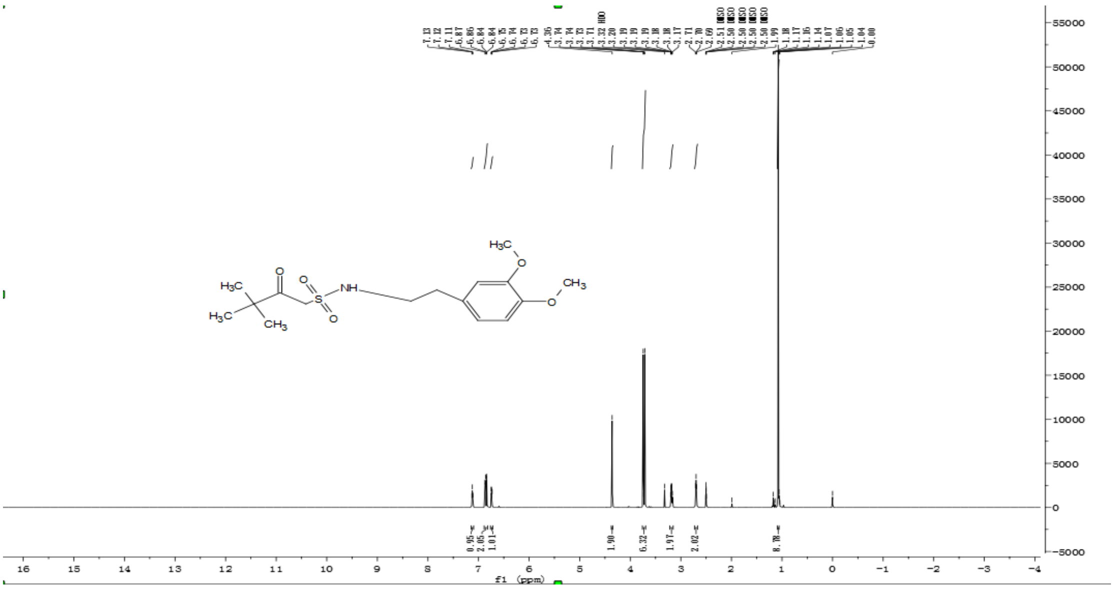

Figure S71. ^{1}H NMR of compound *P-36*

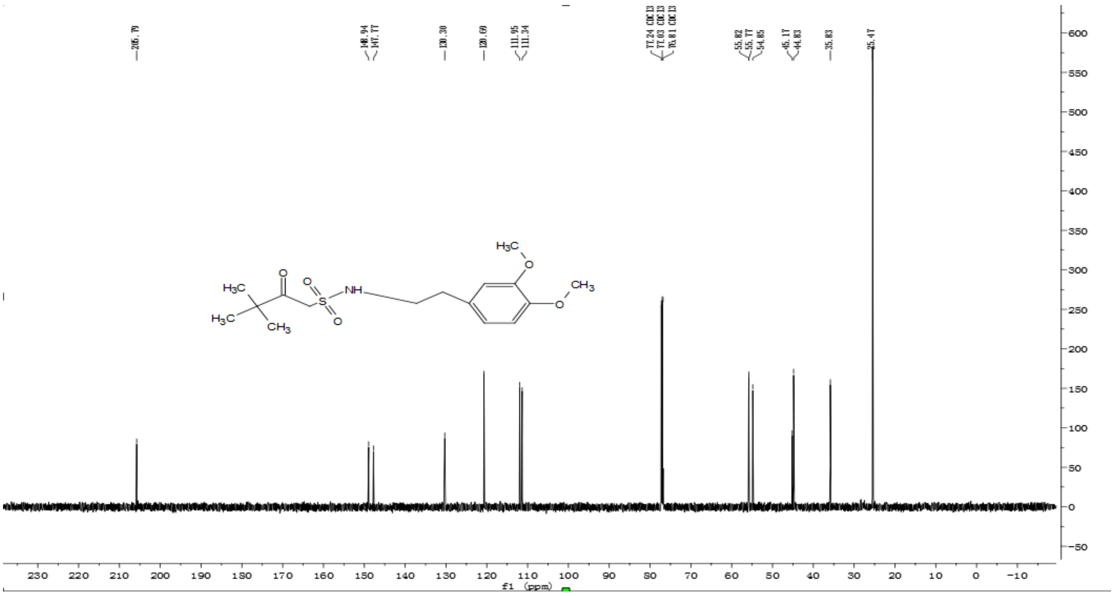

Figure S72. ^{13}C NMR of compound *P-36*

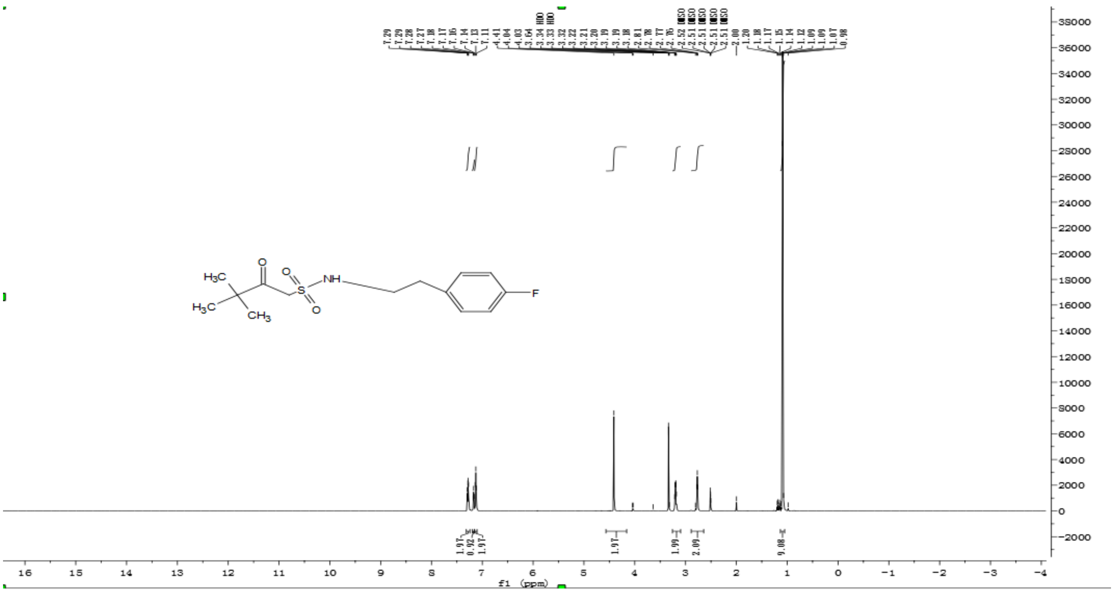

Figure S73. ^{1}H NMR of compound *P-37*

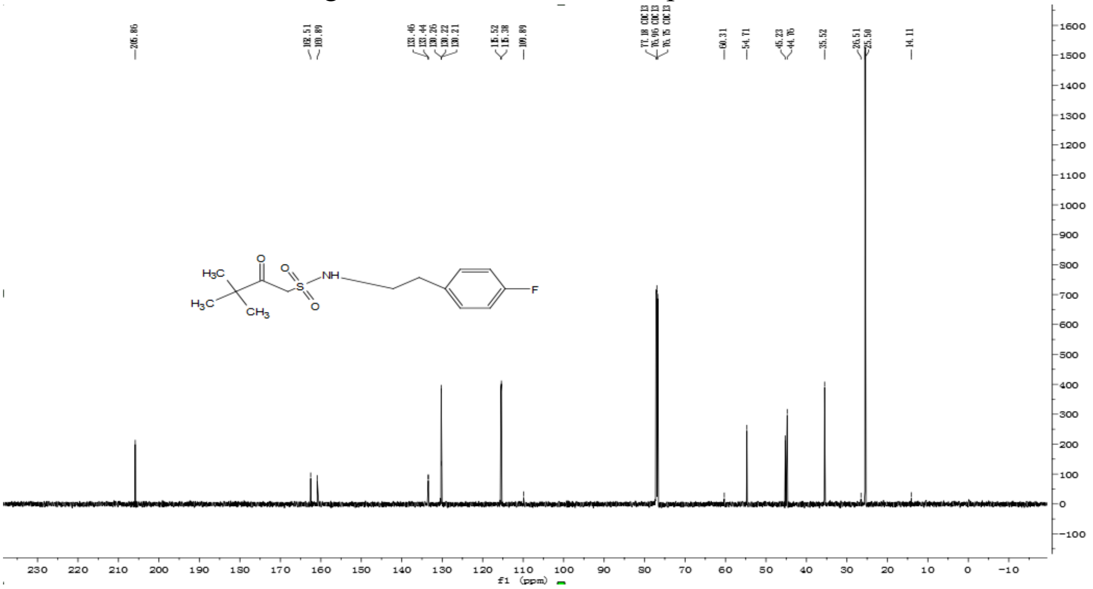

Figure S74. ^{13}C NMR of compound *P-37*

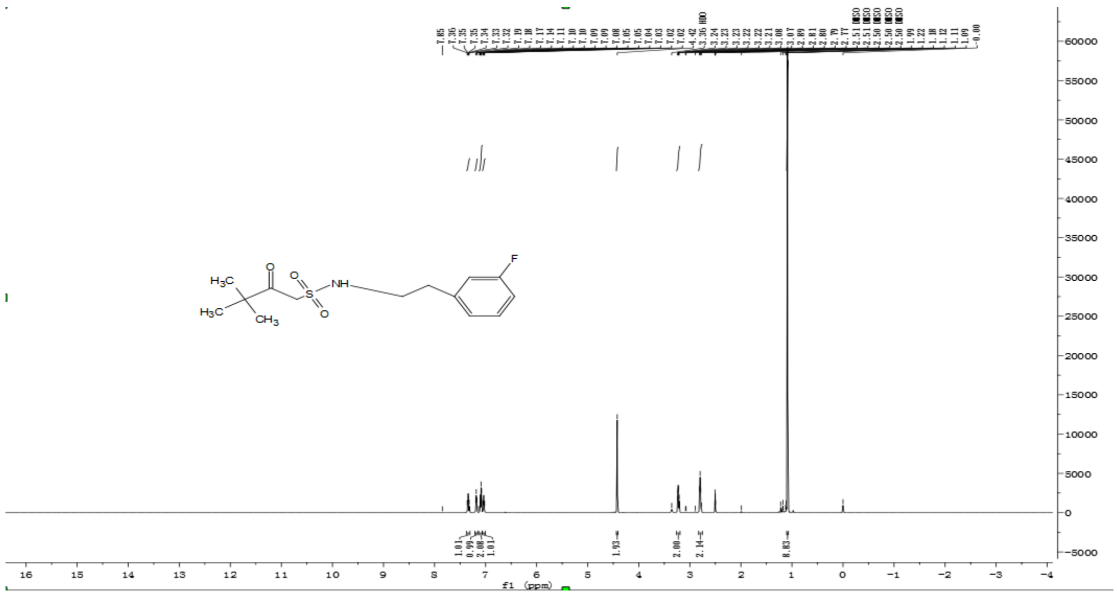

Figure S75. ^{1}H NMR of compound *P-38*

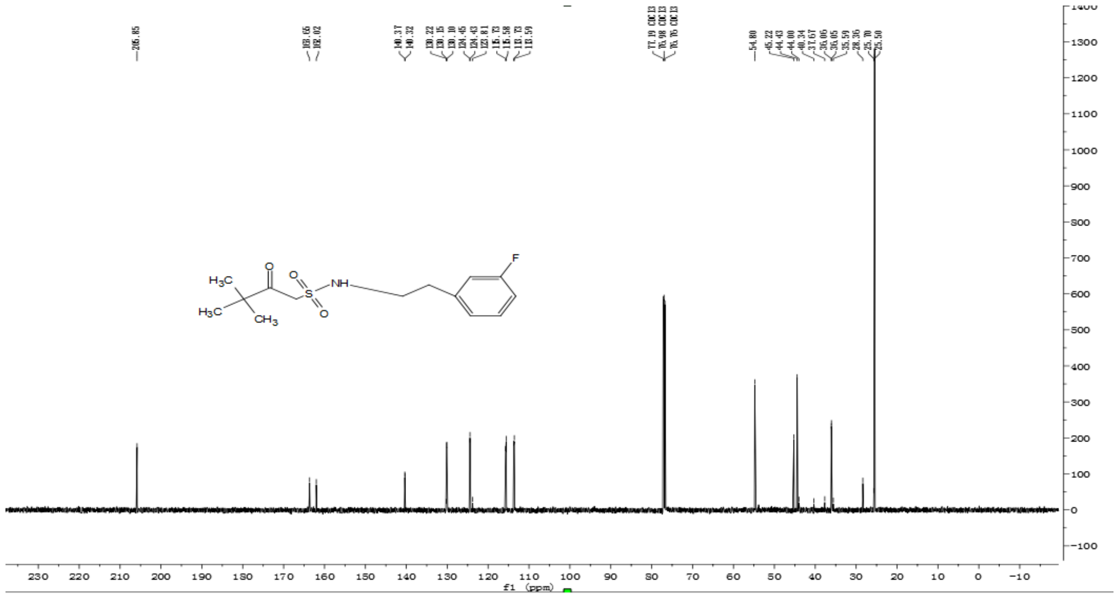

Figure S76. ^{13}C NMR of compound *P-38*

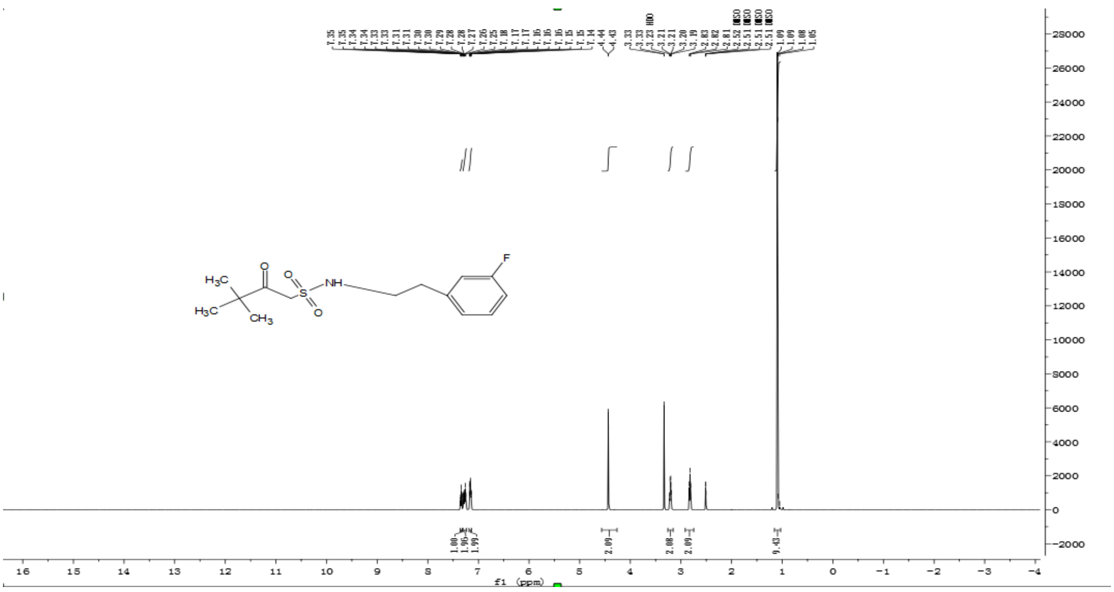

Figure S77. ^{1}H NMR of compound *P-39*

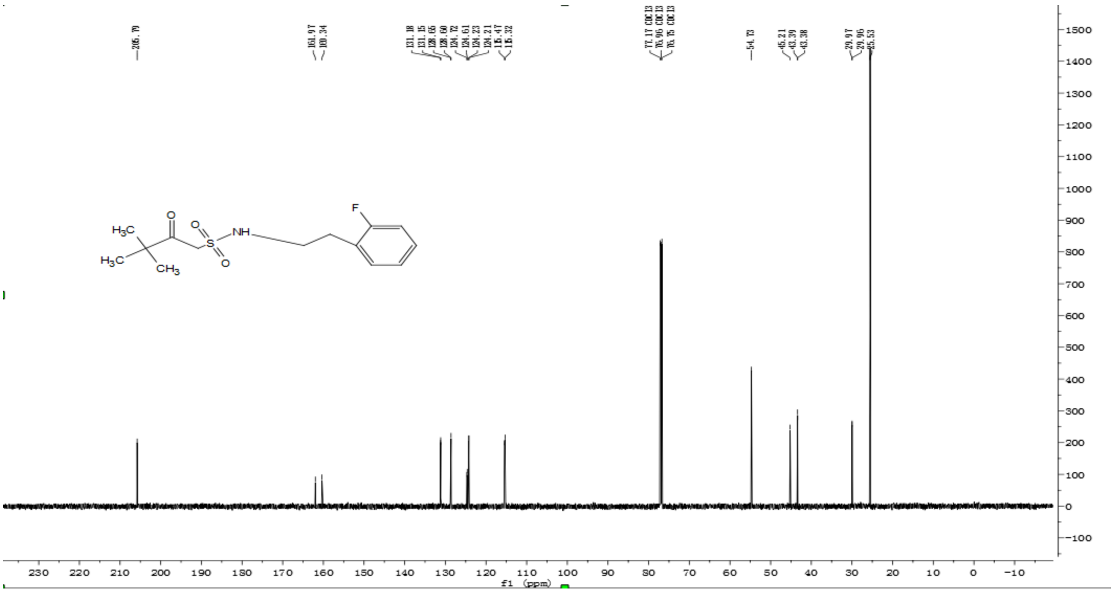

Figure S78. ^{13}C NMR of compound *P-39*

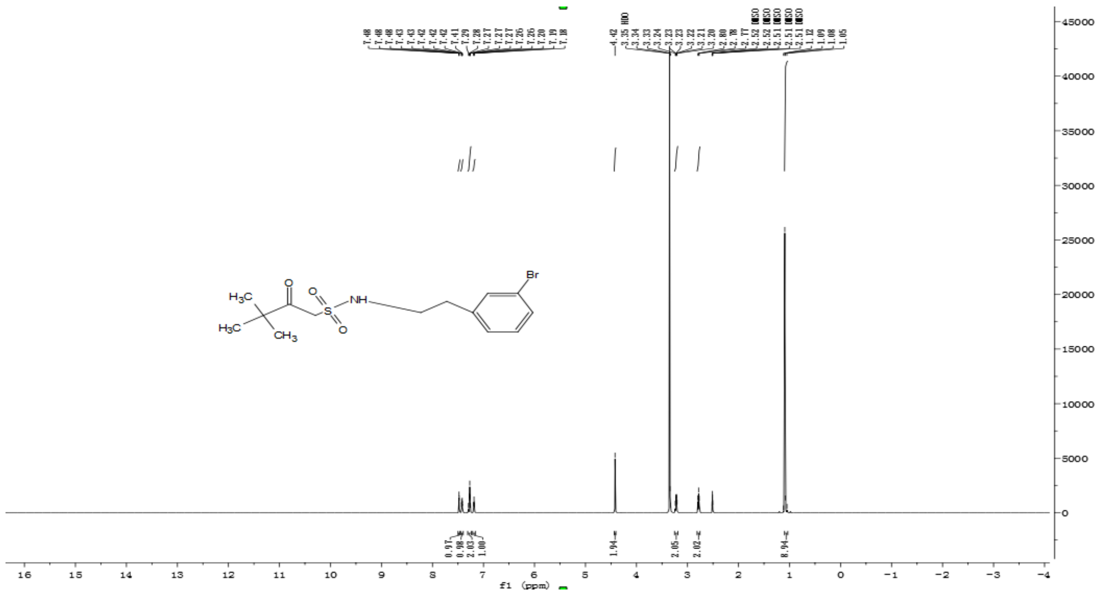

Figure S79. ^{1}H NMR of compound *P-40*

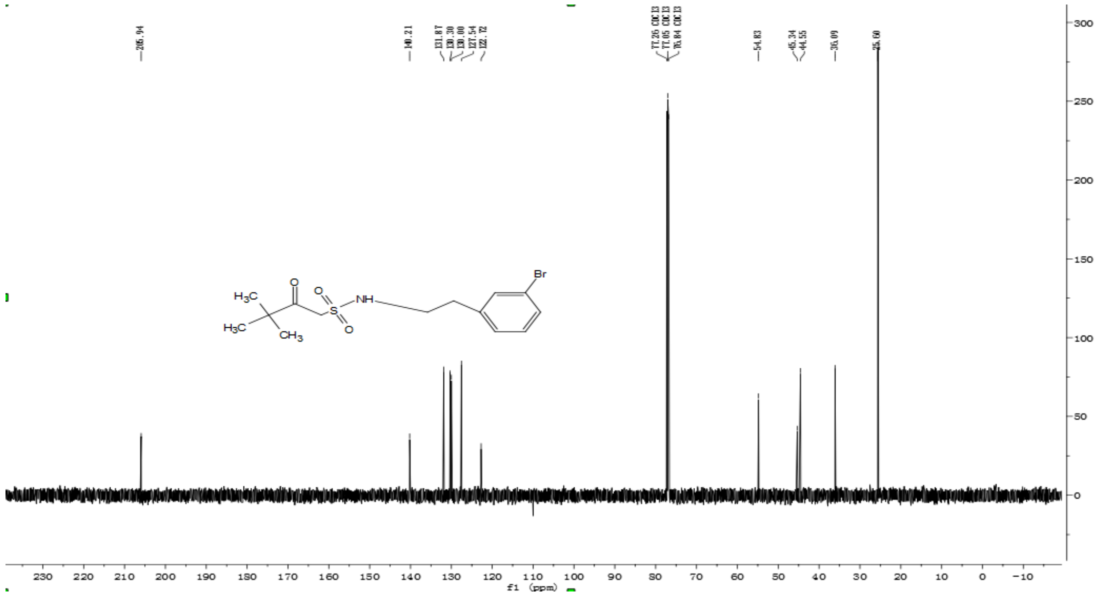

Figure S80. ^{13}C NMR of compound *P-40*

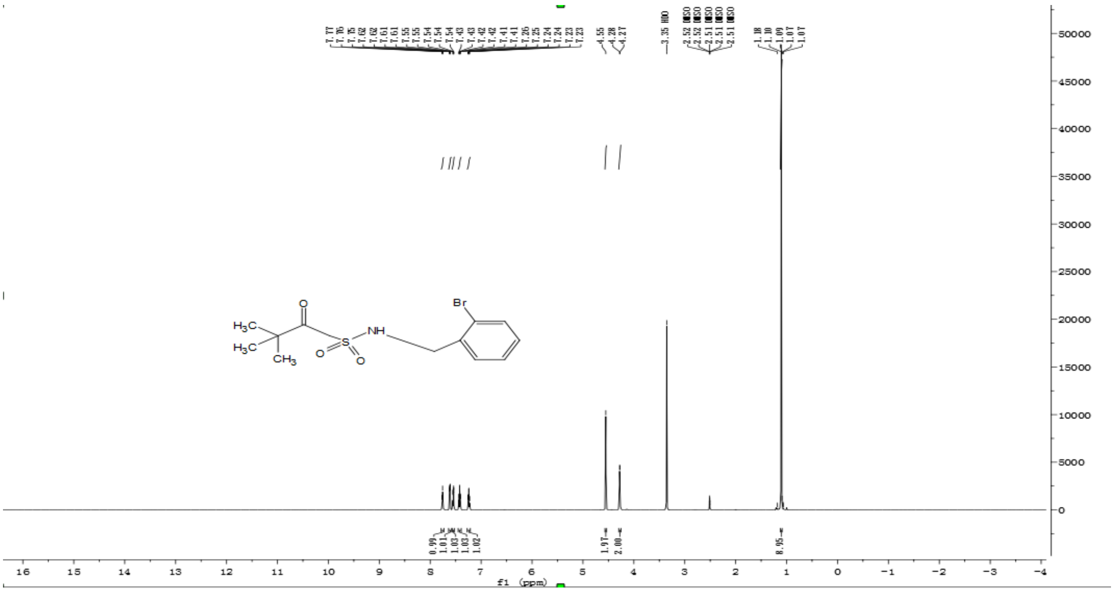

Figure S81. ^{1}H NMR of compound *P-41*

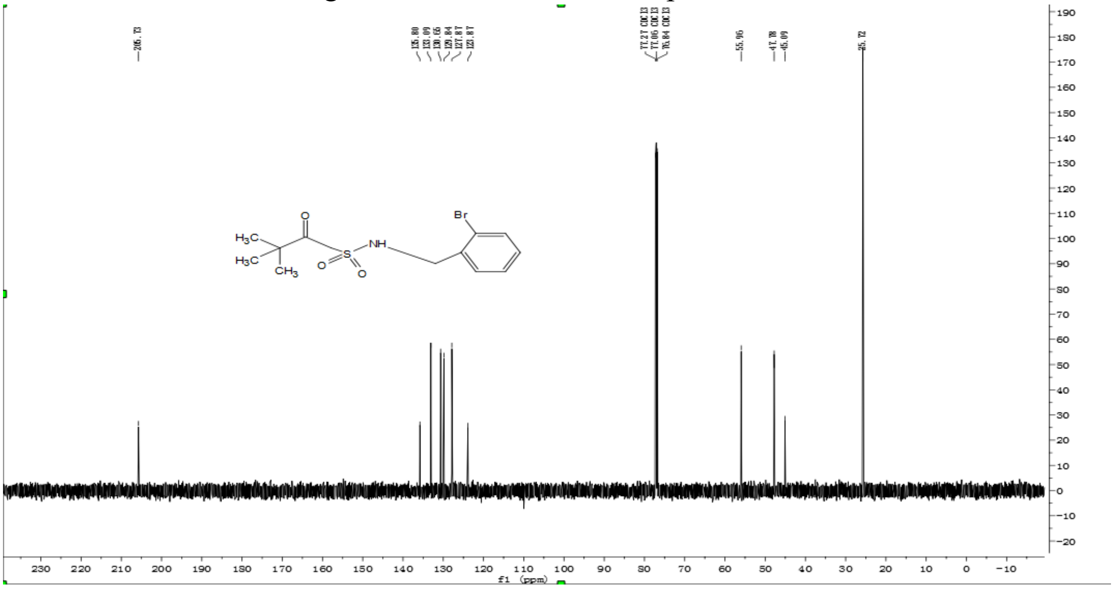

Figure S82. ^{13}C NMR of compound *P-41*

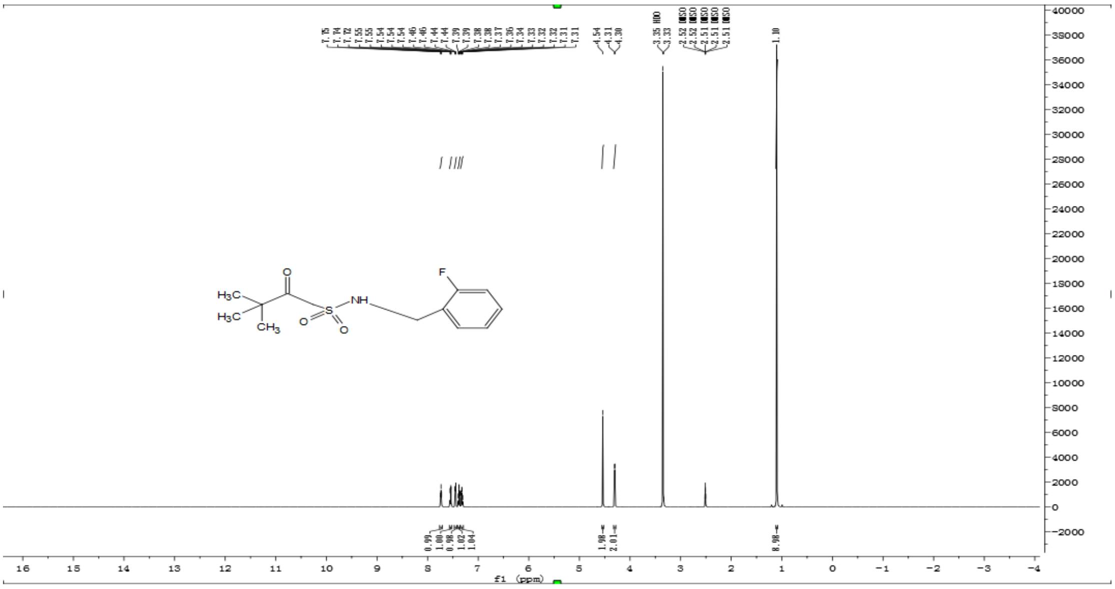

Figure S83. ^{1}H NMR of compound *P-42*

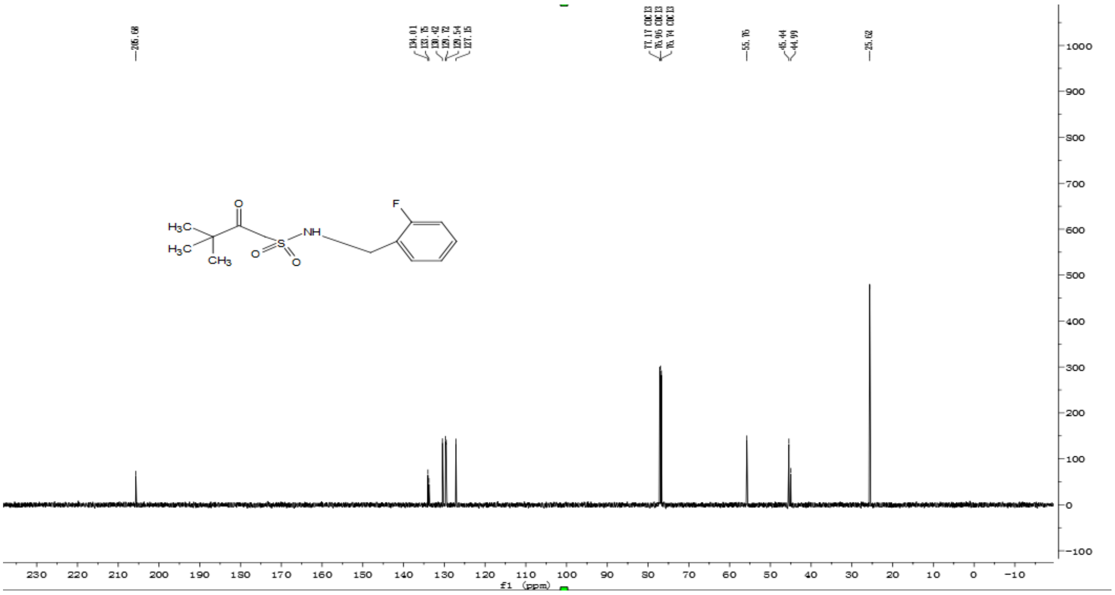

Figure S84. ^{13}C NMR of compound *P-42*

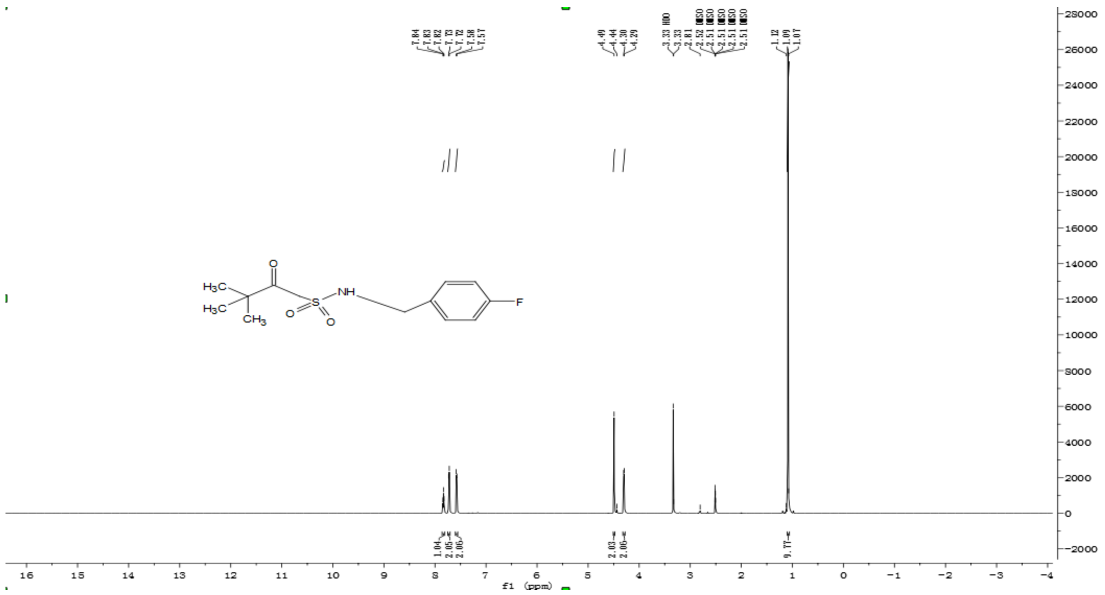

Figure S85. ^{1}H NMR of compound *P-43*

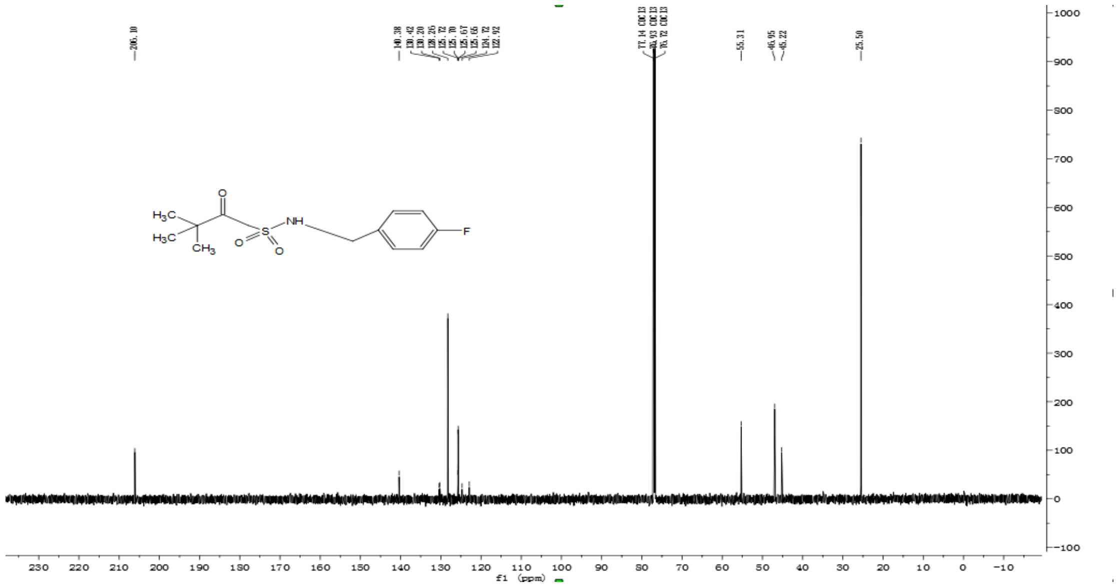

Figure S86. ^{13}C NMR of compound *P-43*

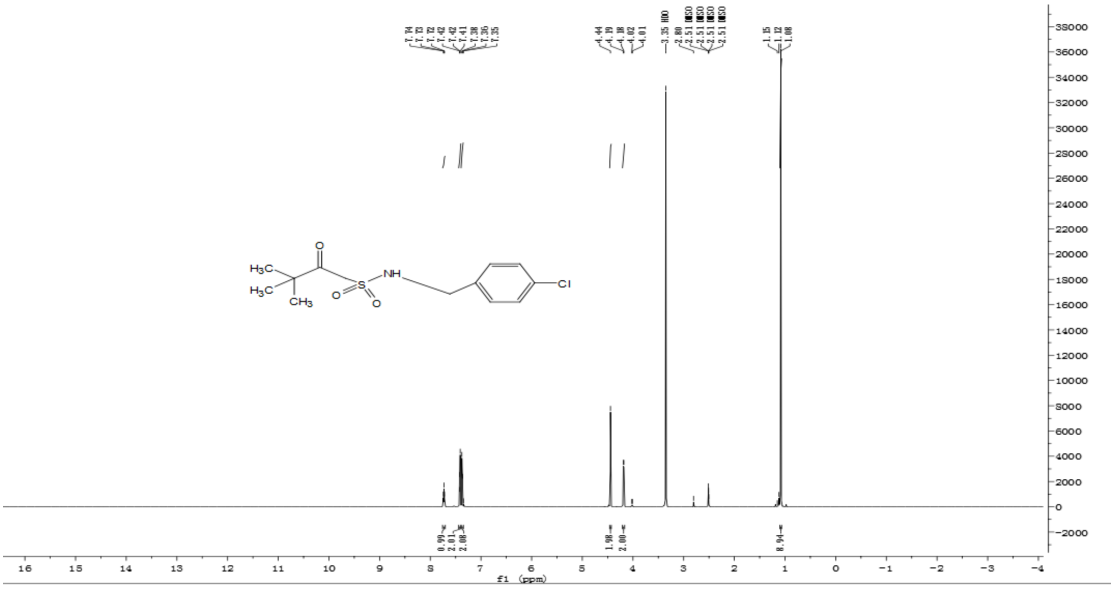

Figure S87. ^{1}H NMR of compound *P-44*

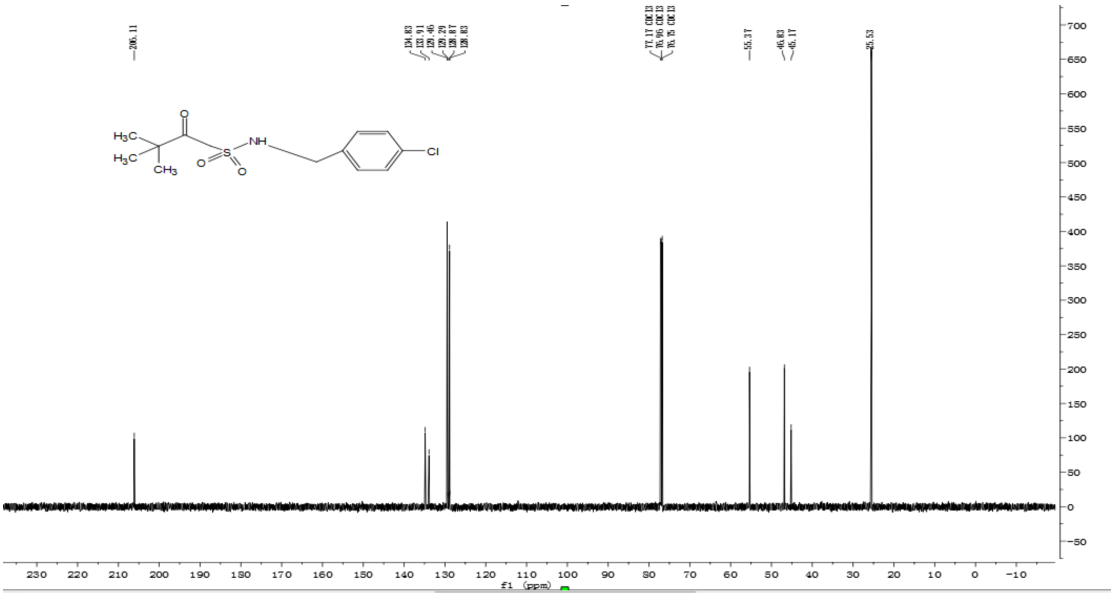

Figure S88. ^{13}C NMR of compound *P-44*

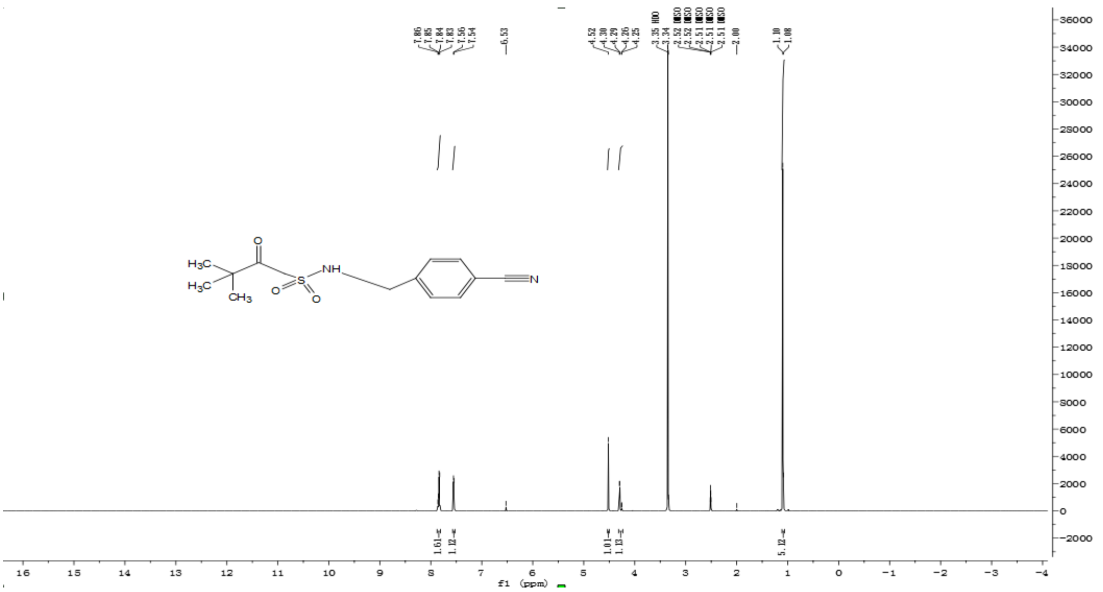

Figure S89. ^{1}H NMR of compound *P-45*

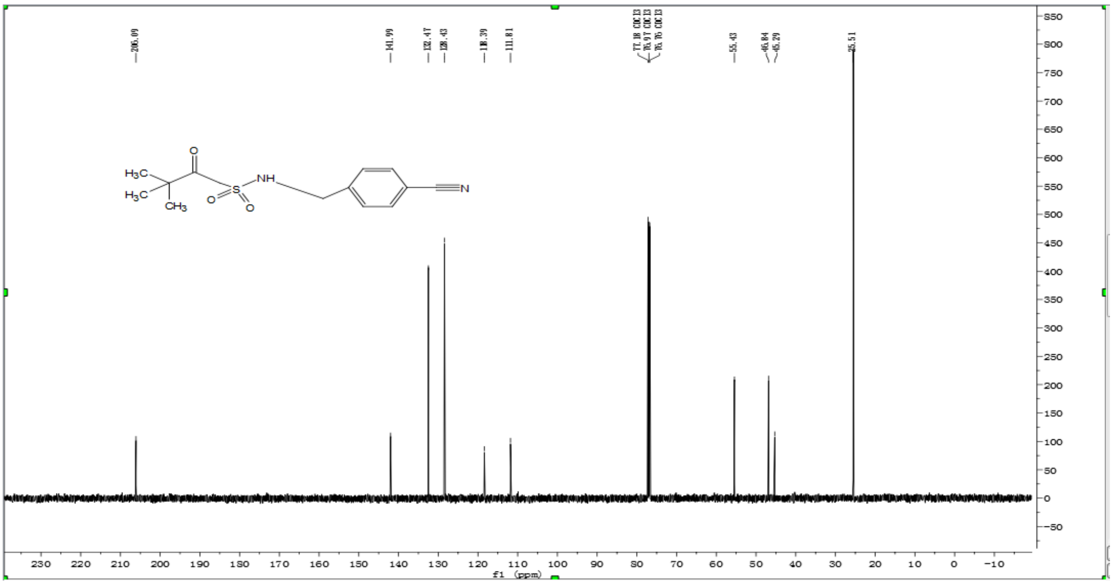

Figure S90. ^{13}C NMR of compound *P-45*

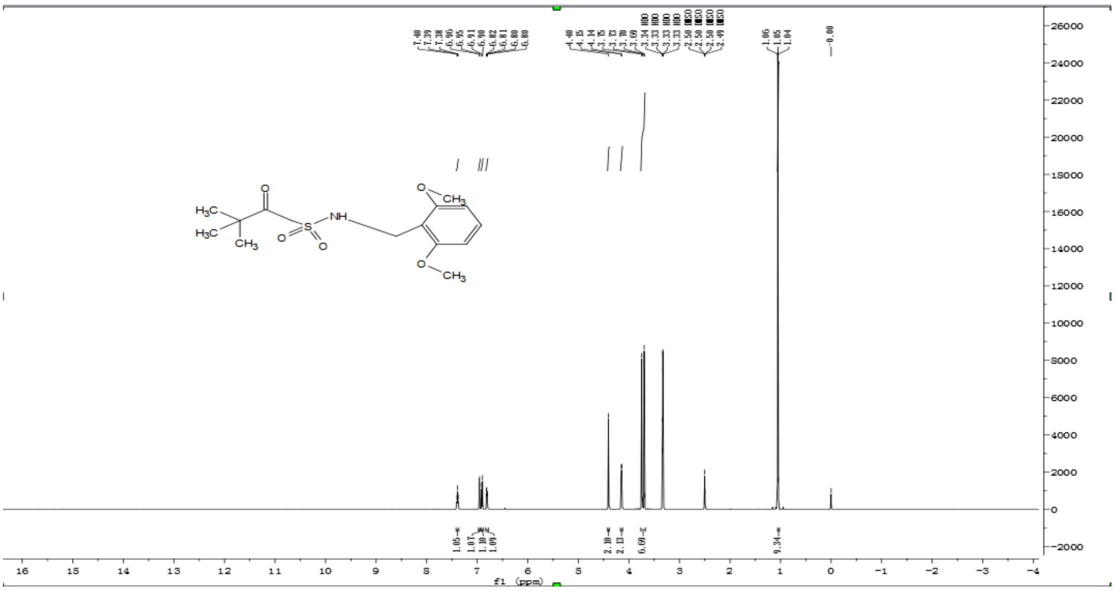

Figure S91. ^{1}H NMR of compound *P-46*

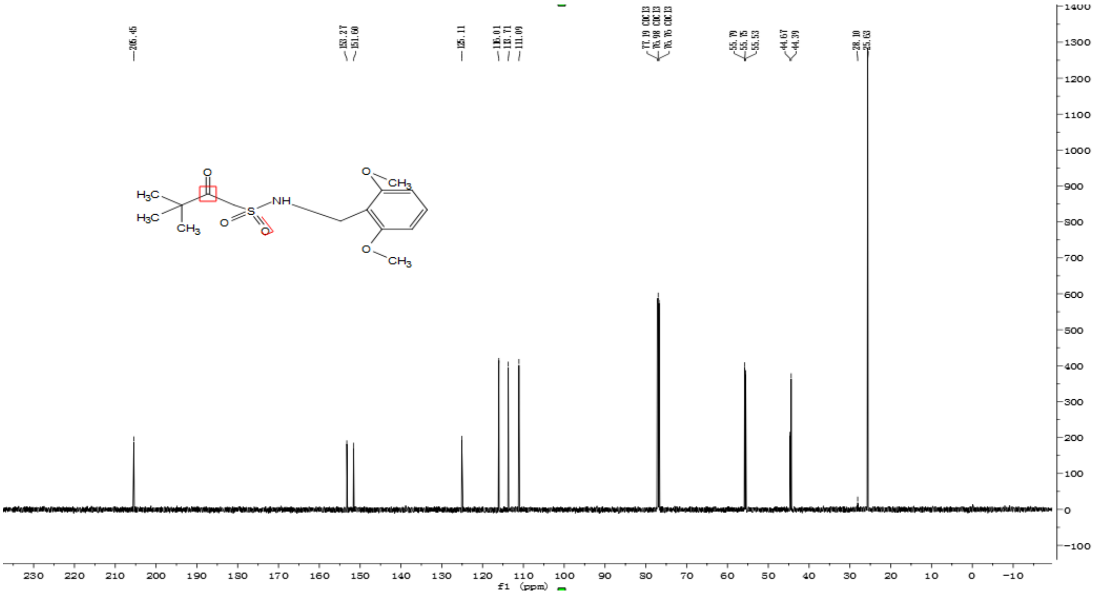

Figure S92. ^{13}C NMR of compound *P-46*

2. MS of P-1~P-46

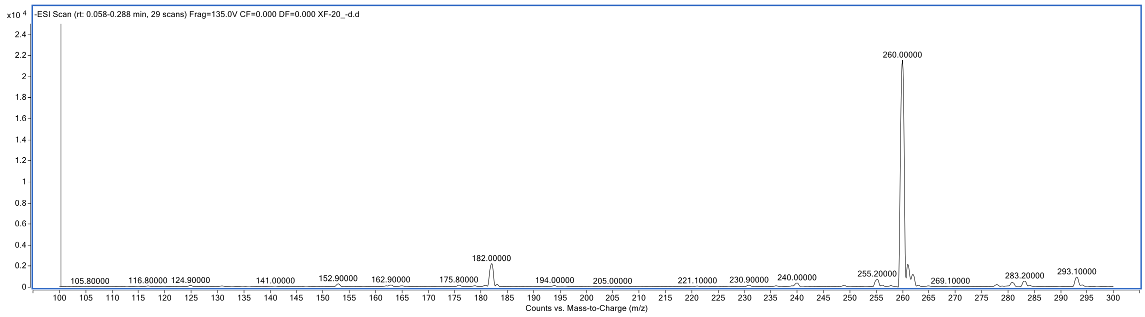

Figure S93. MS of compound *P-1*

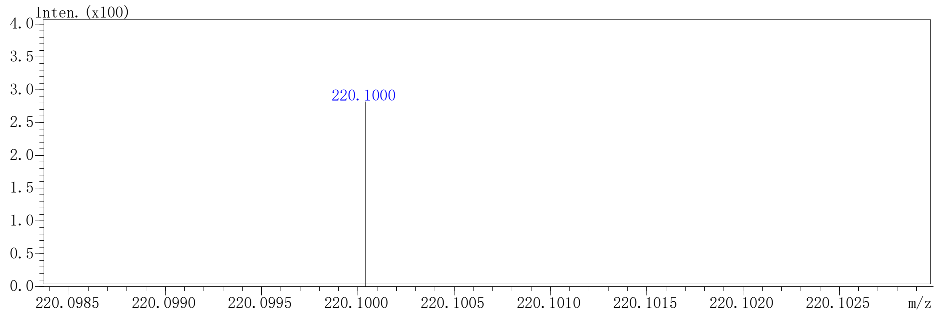

Figure S94. MS of compound *P-2*

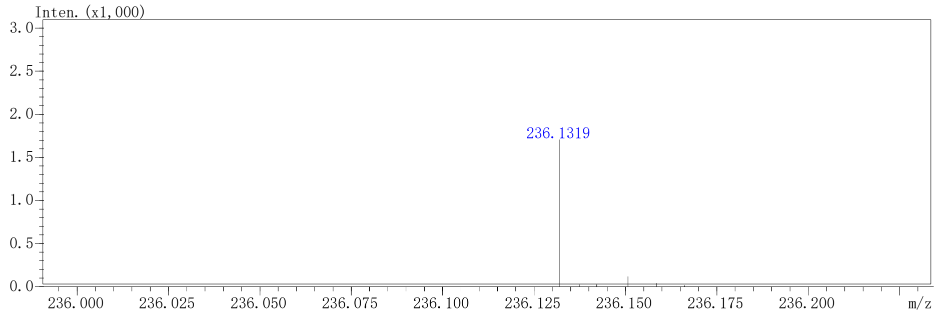

Figure S95. MS of compound *P-3*

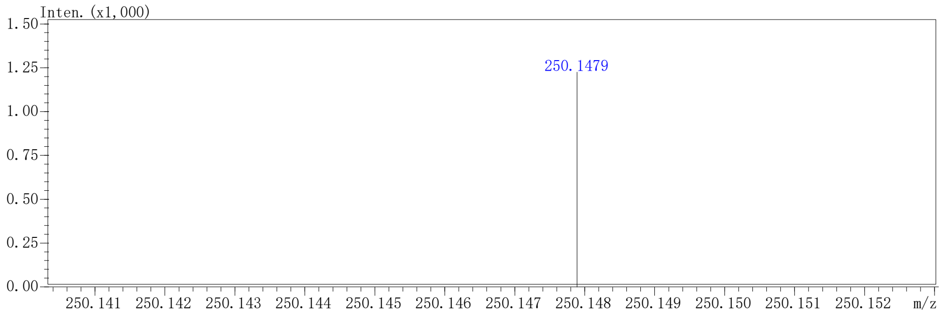

Figure S96. MS of compound *P-4*

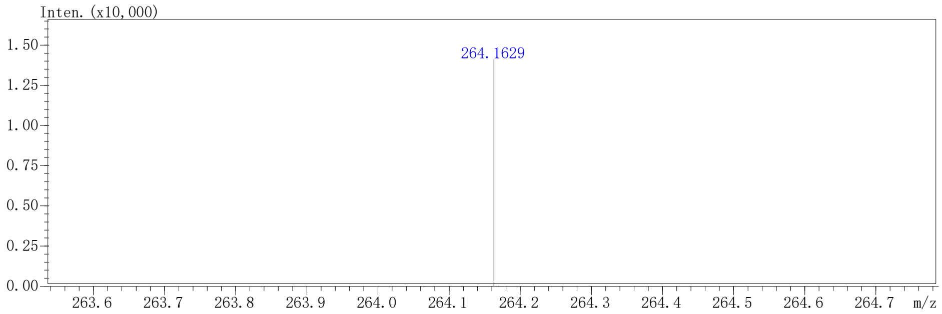

Figure S97. MS of compound *P-5*

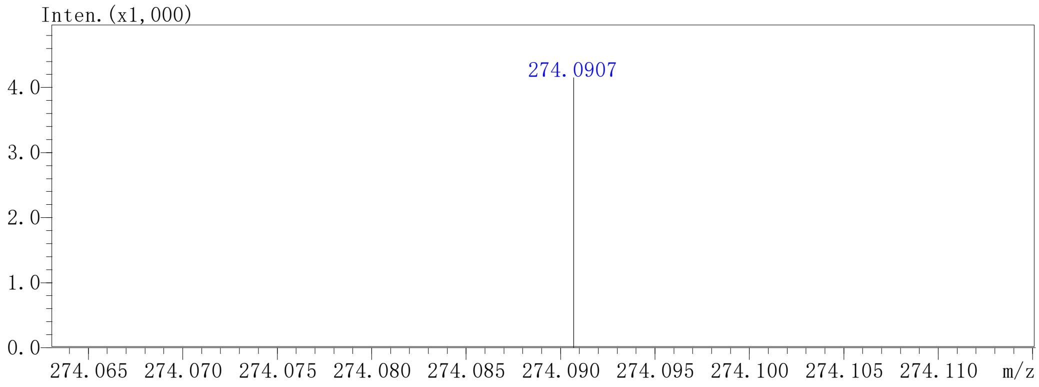

Figure S98. MS of compound *P-6*

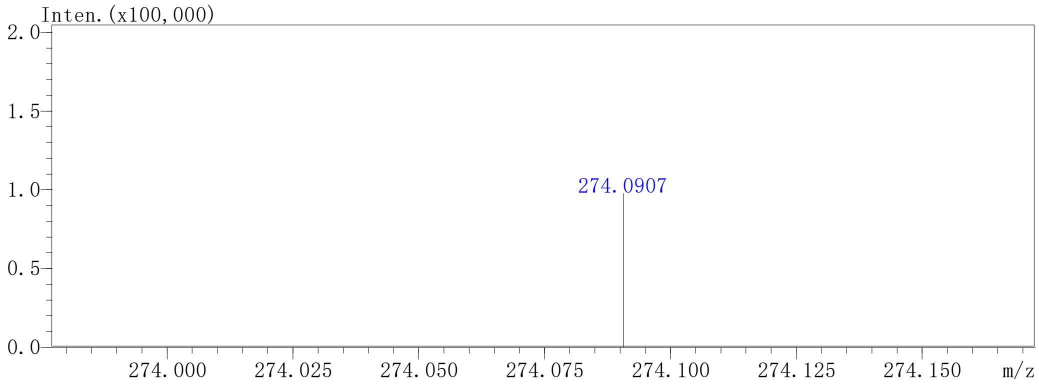

Figure S99. MS of compound *P-7*

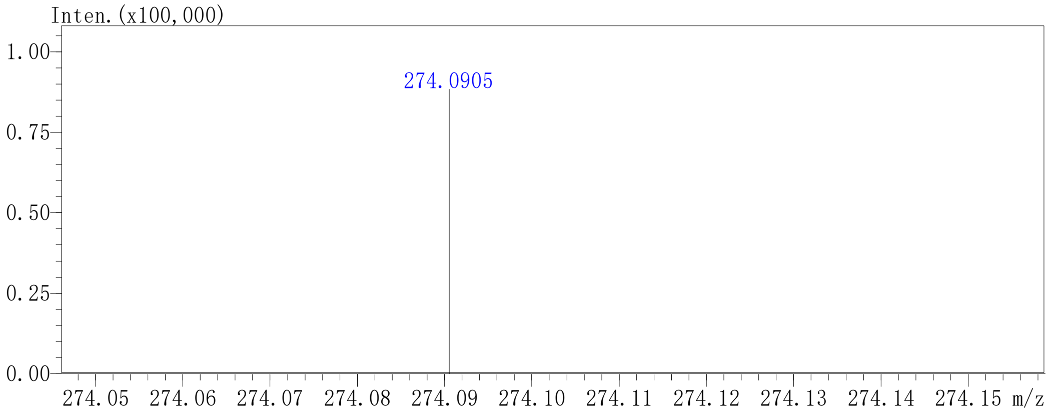

Figure S100. MS of compound *P-8*

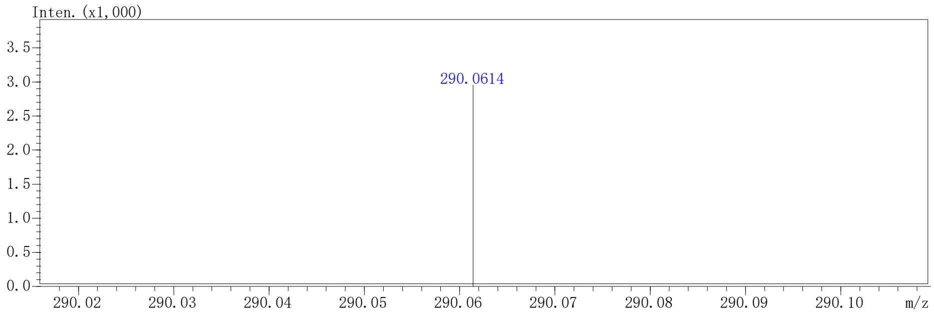

Figure S101. MS of compound *P-9*

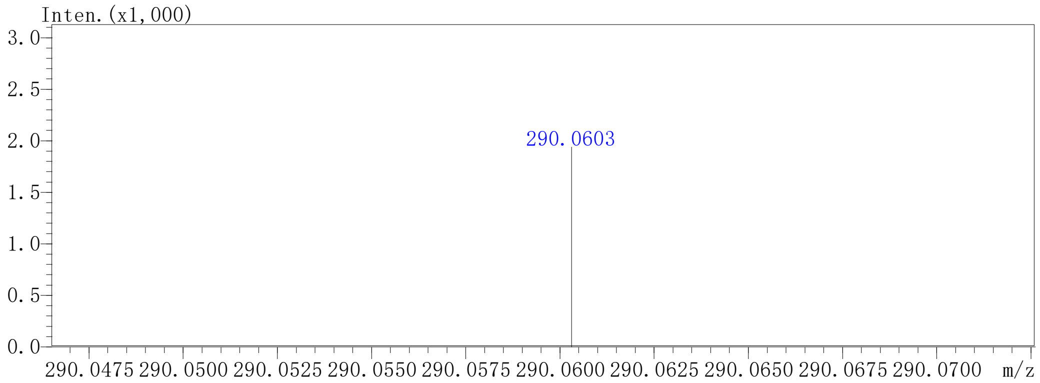

Figure S102. MS of compound *P-10*

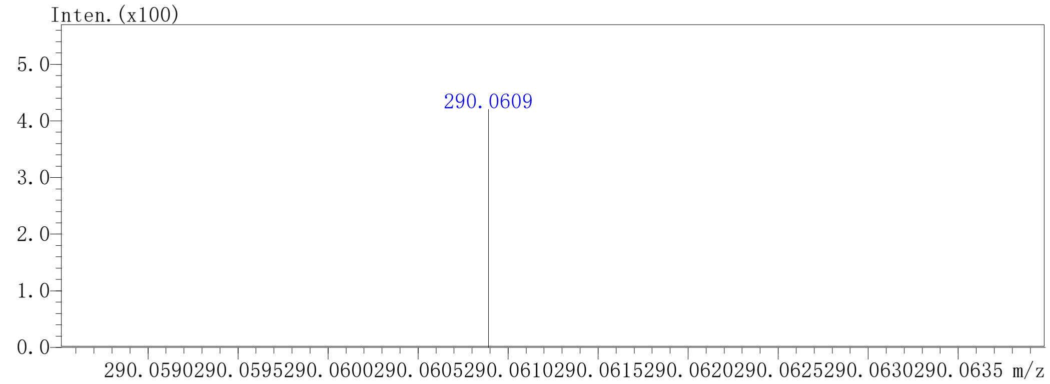

Figure S103. MS of compound *P-11*

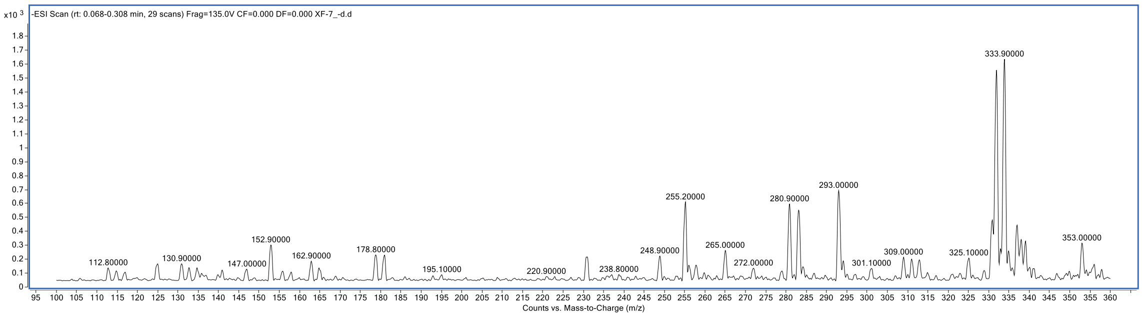

Figure S104. MS of compound *P-12*

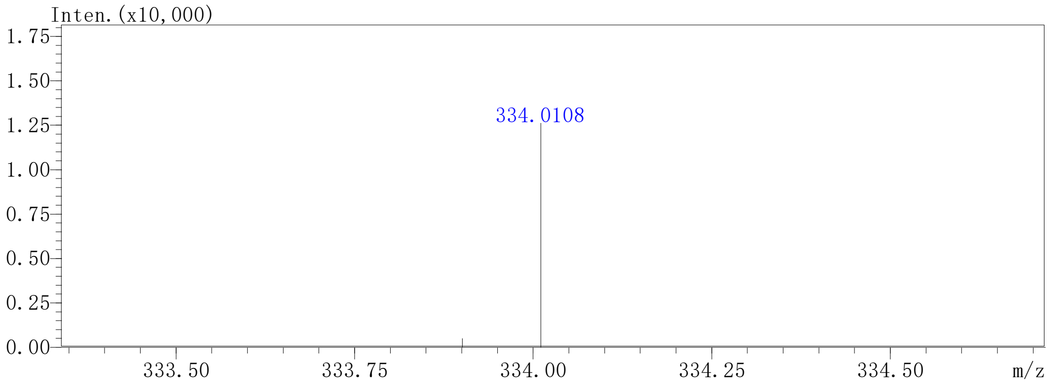

Figure S105. MS of compound *P-13*

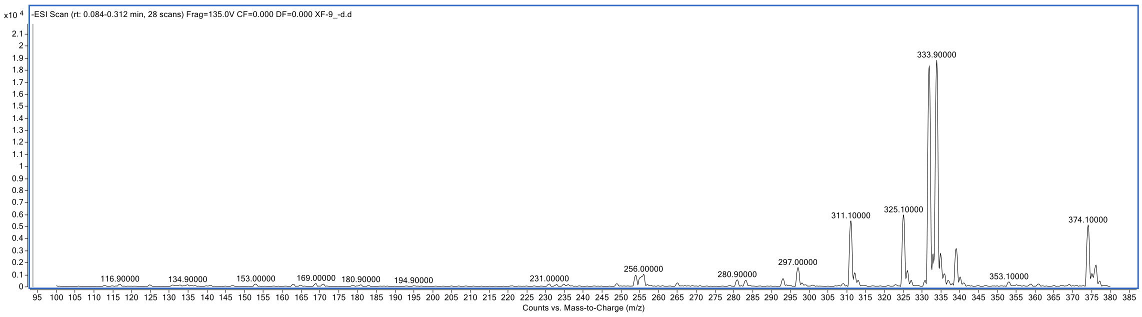

Figure S106. MS of compound *P-14*

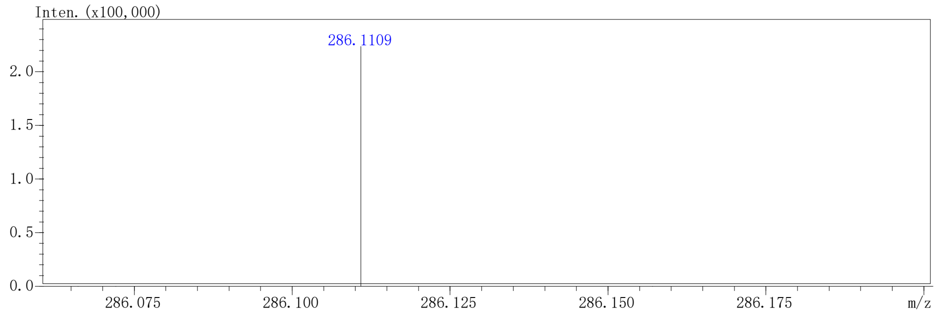

Figure S107. MS of compound *P-15*

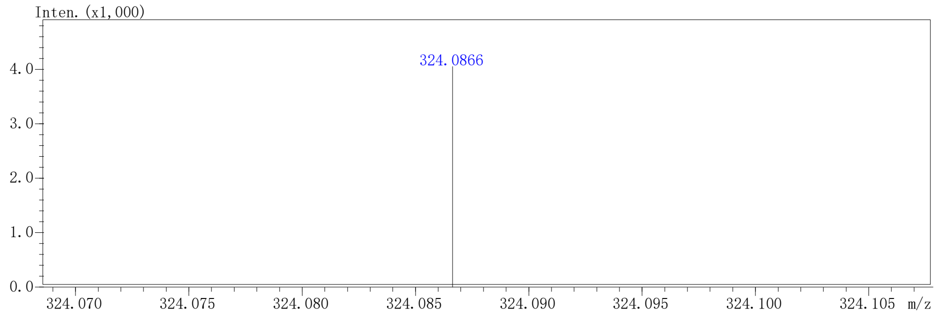

Figure S108. MS of compound *P-16*

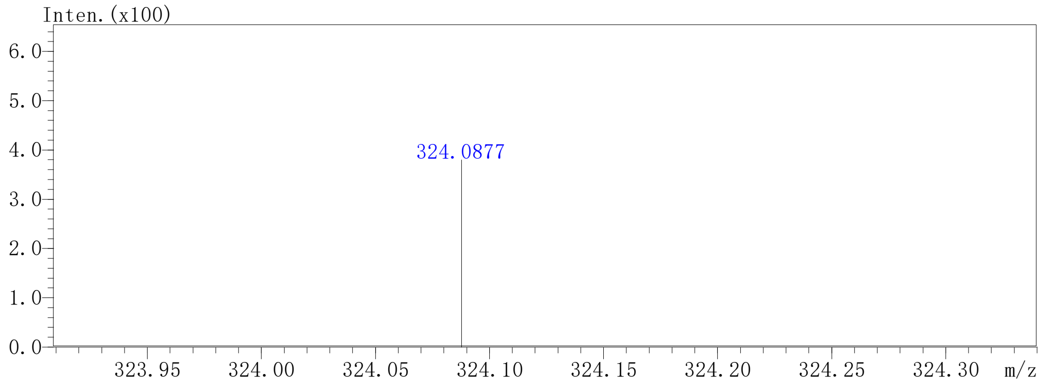

Figure S109. MS of compound *P-17*

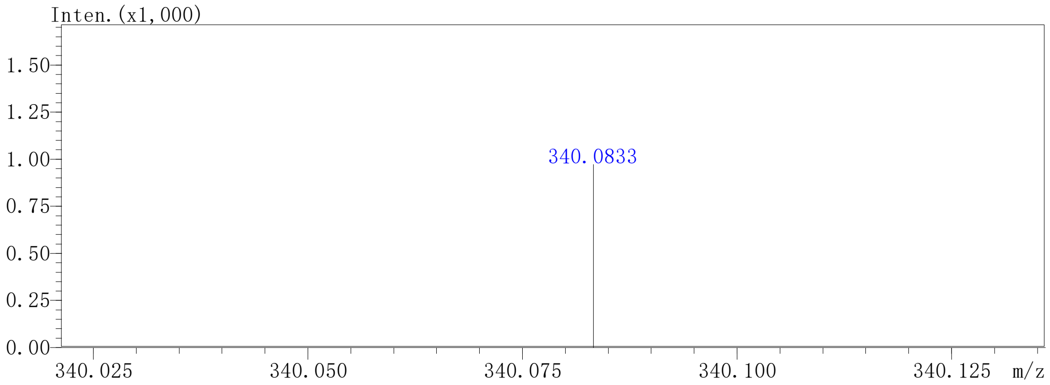

Figure S110. MS of compound *P-18*

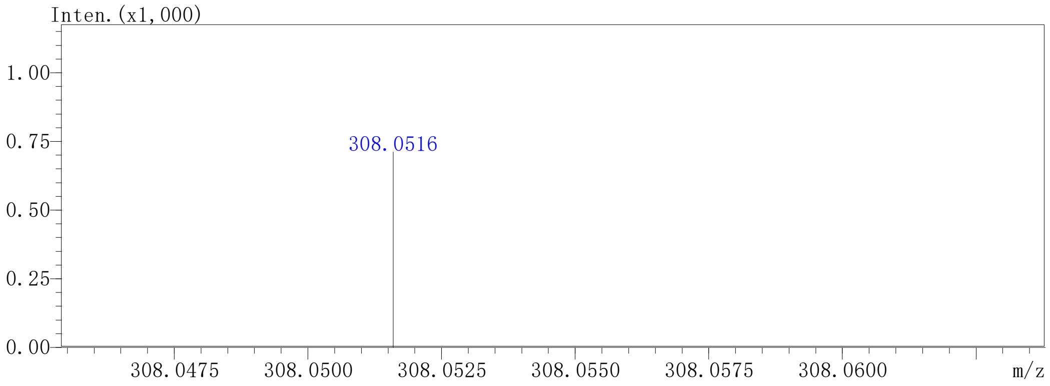

Figure S111. MS of compound *P-19*

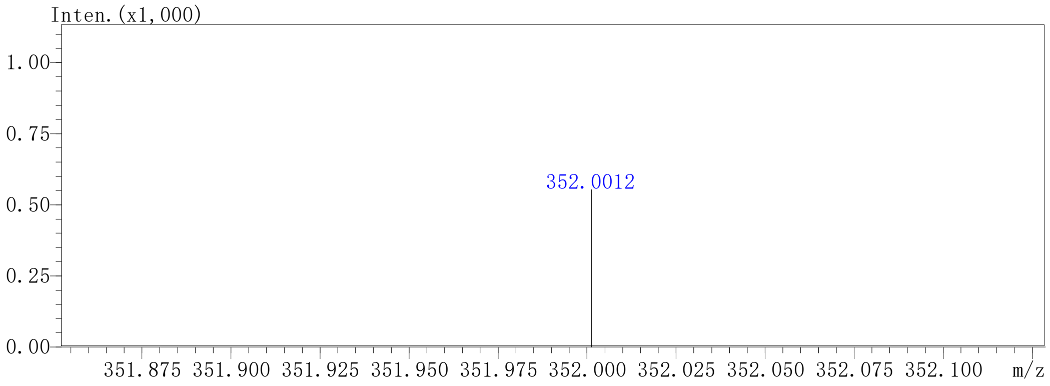

Figure 112. MS of compound *P-20*

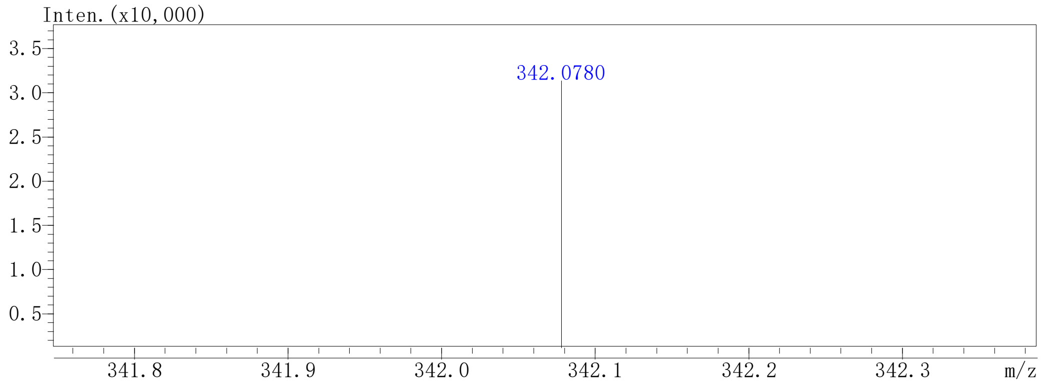

Figure S113. MS of compound *P-21*

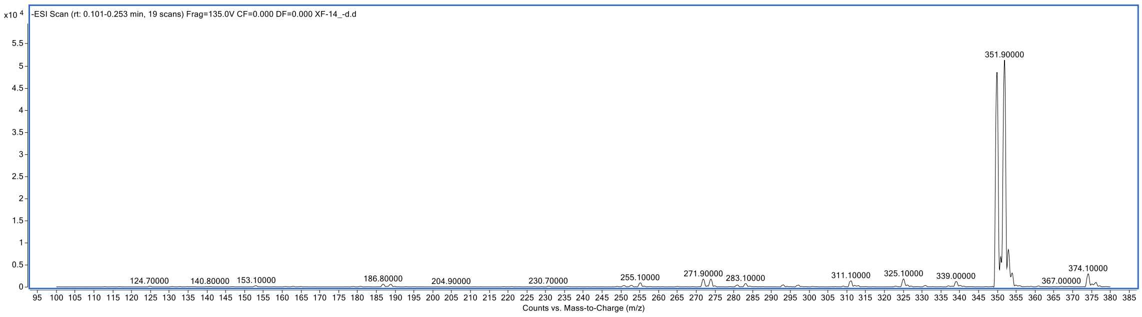

Figure S114. MS of compound *P-22*

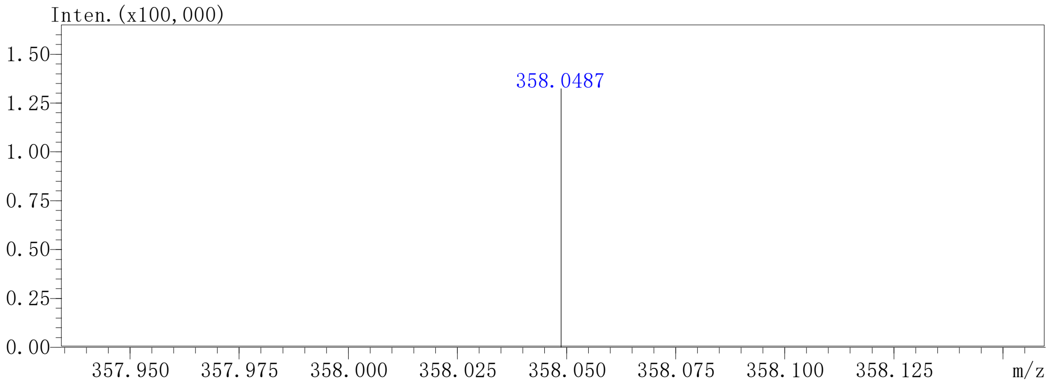

Figure S115. MS of compound *P-23*

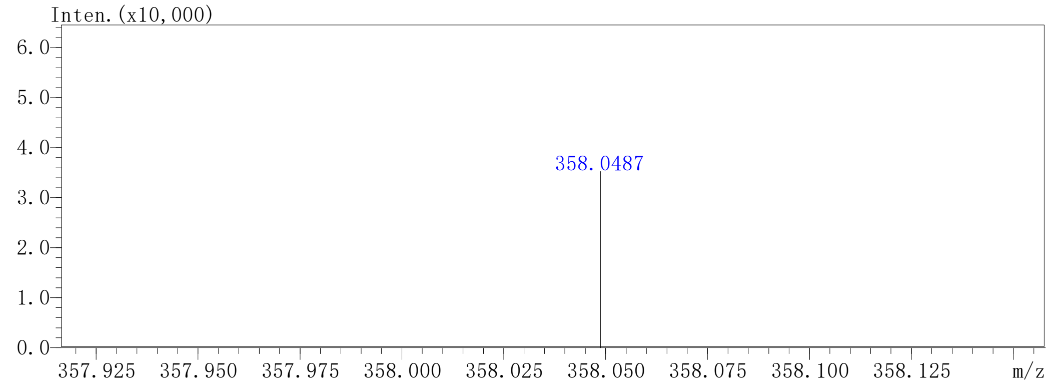

Figure S116. MS of compound *P-24*

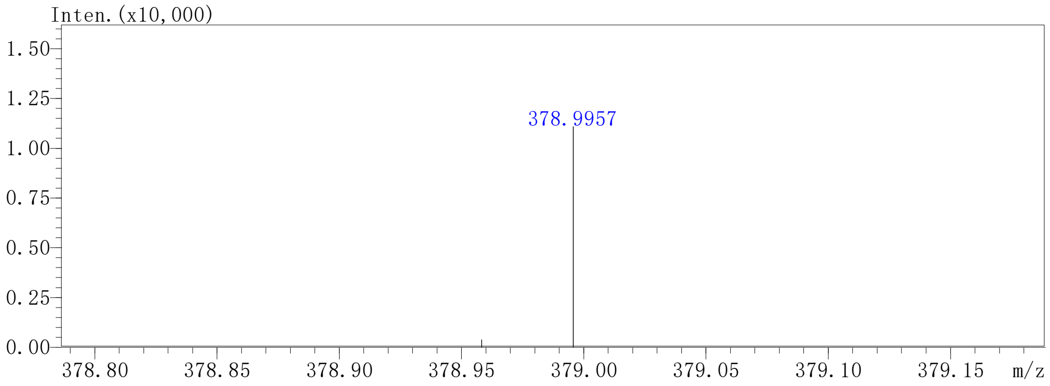

Figure S117. MS of compound *P-25*

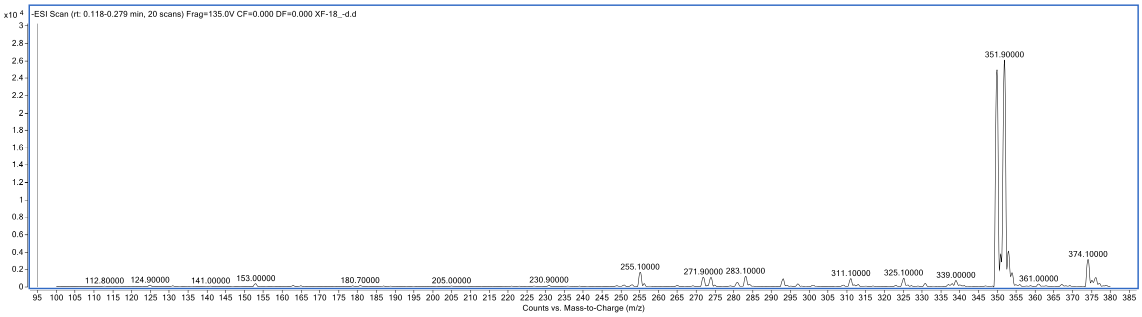

Figure S118. MS of compound *P-26*

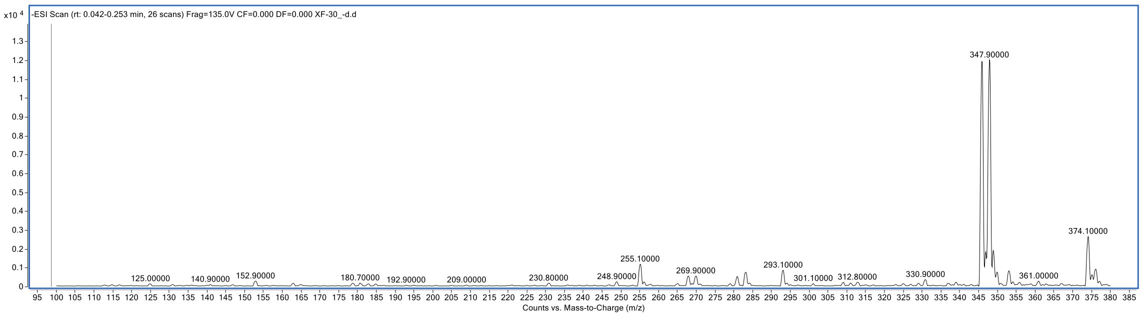

Figure S119. MS of compound *P-27*

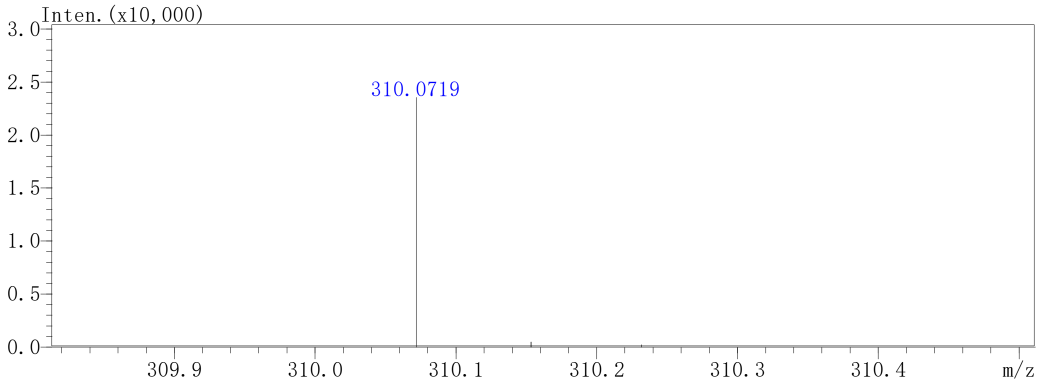

Figure S120. MS of compound *P-28*

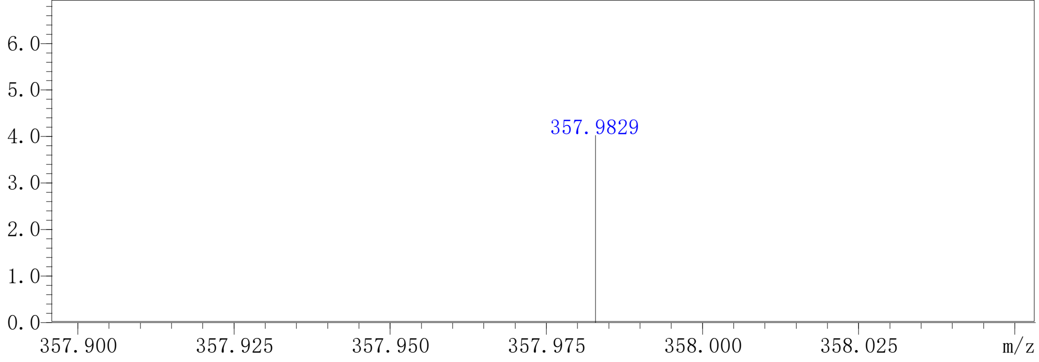

Figure S121. MS of compound *P-29*

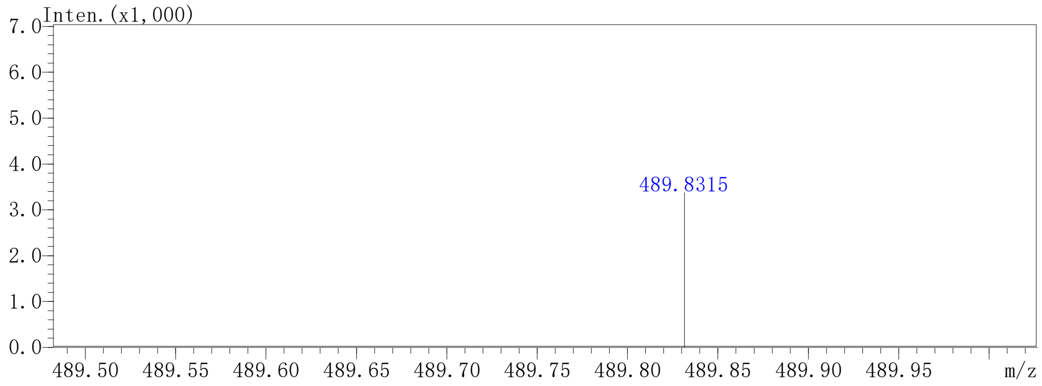

Figure S122. MS of compound *P-30*

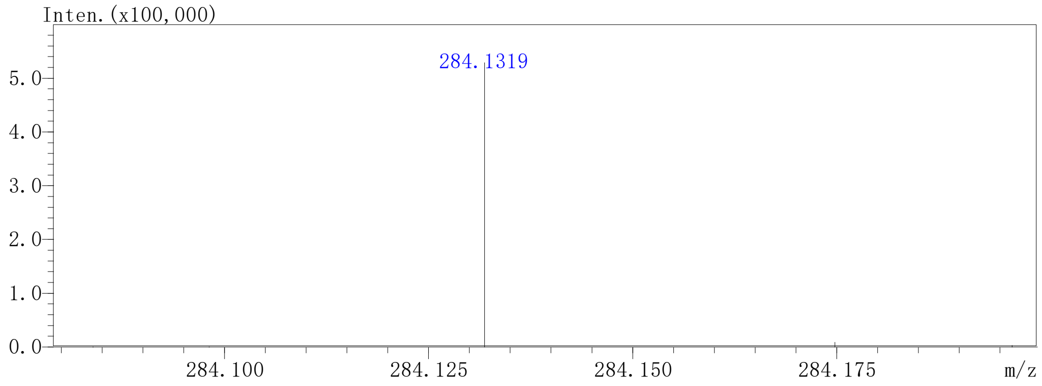

Figure S123. MS of compound *P-31*

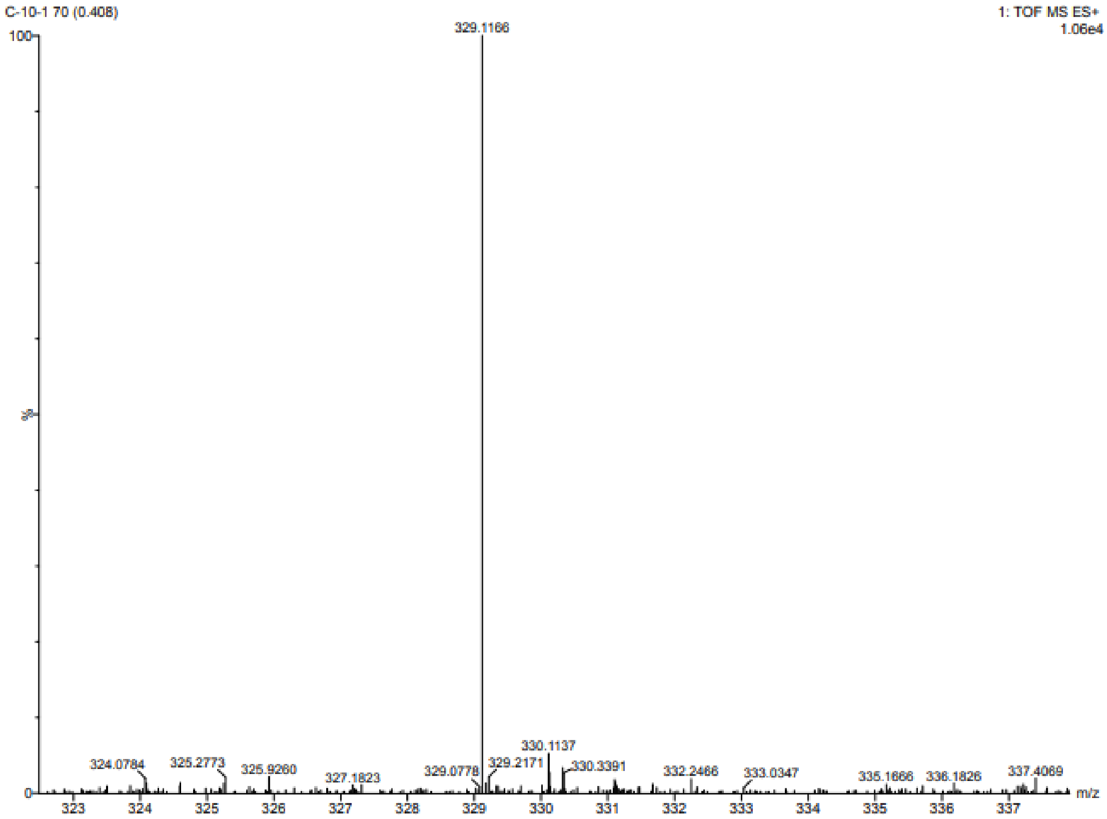

Figure S124. MS of compound *P-32*

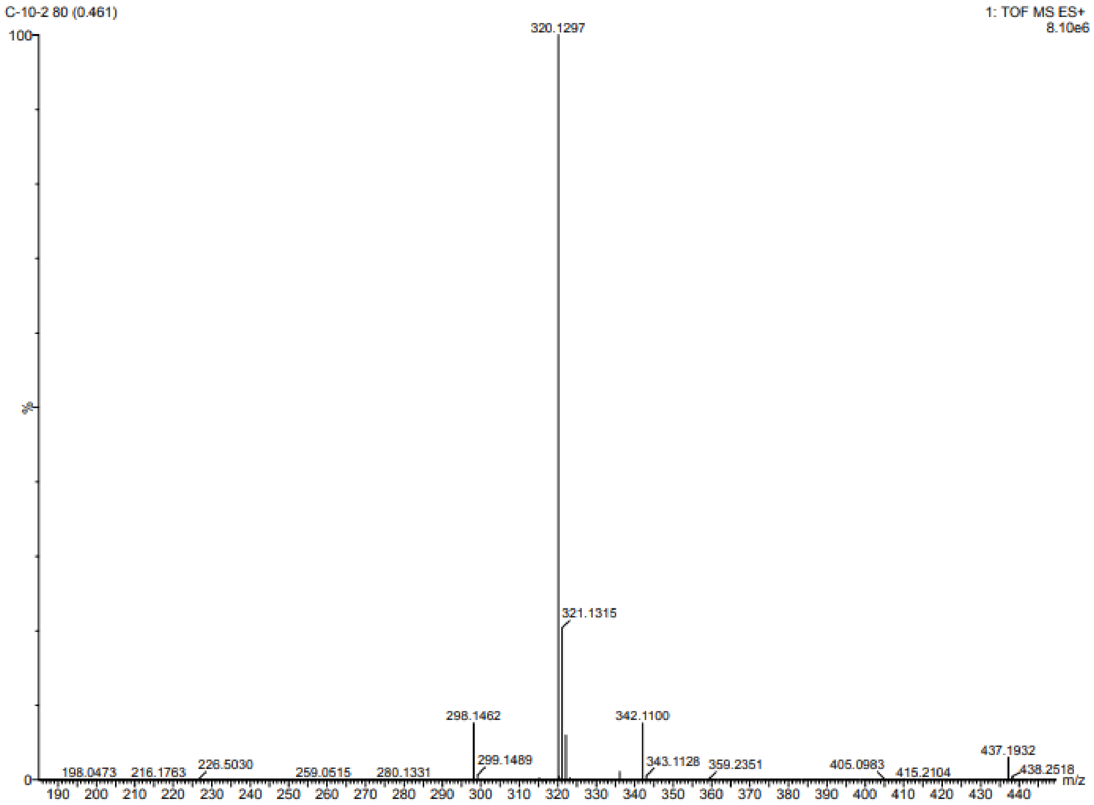

Figure S125. MS of compound *P-33*

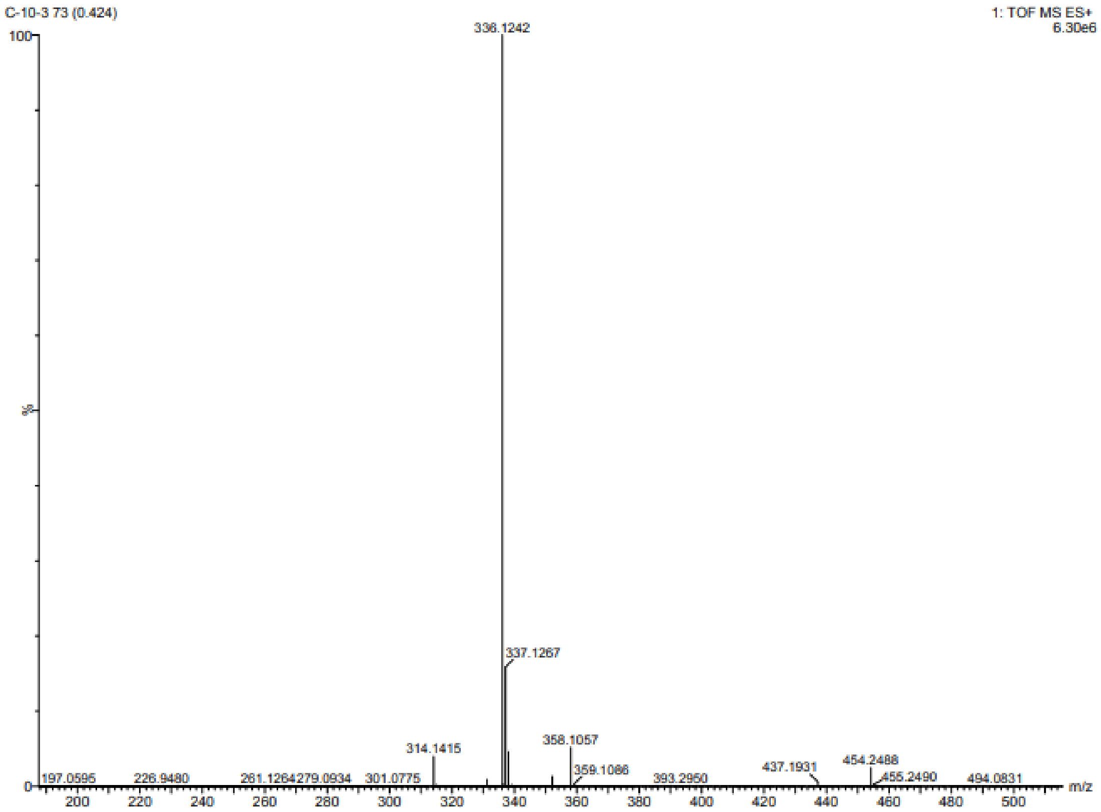

Figure S126. MS of compound *P-34*

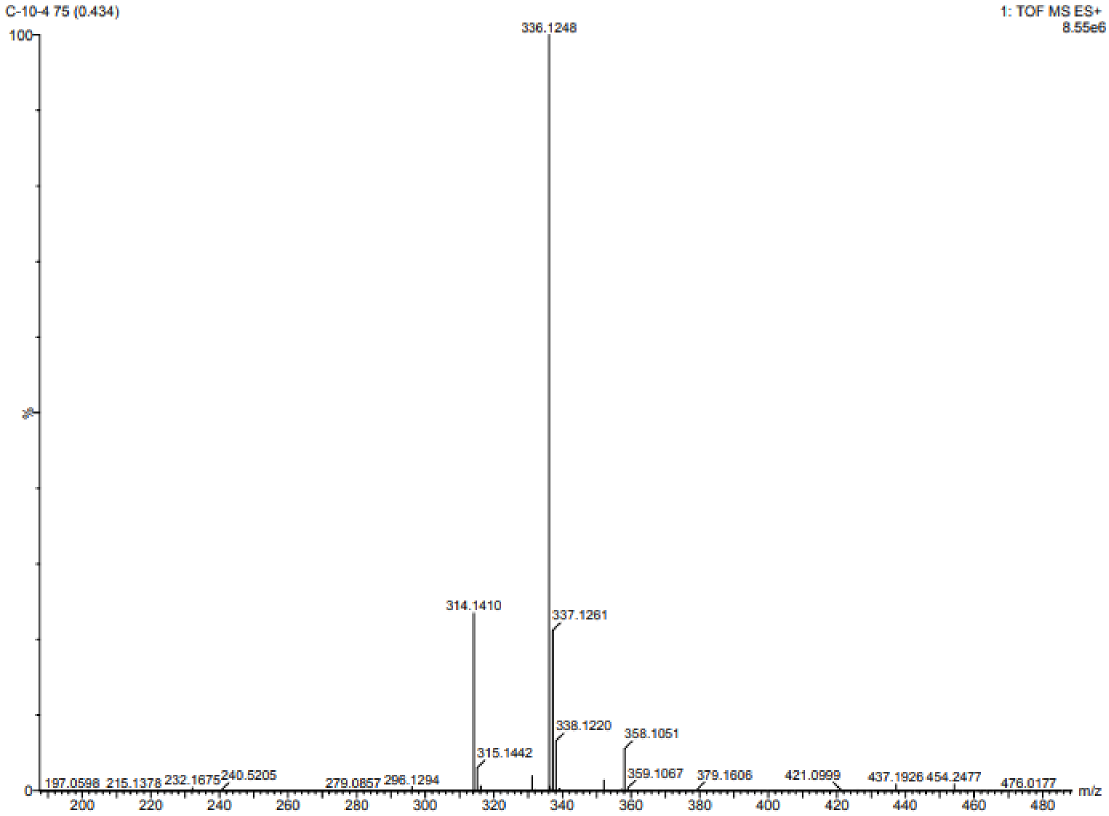

Figure S127. MS of compound *P-35*

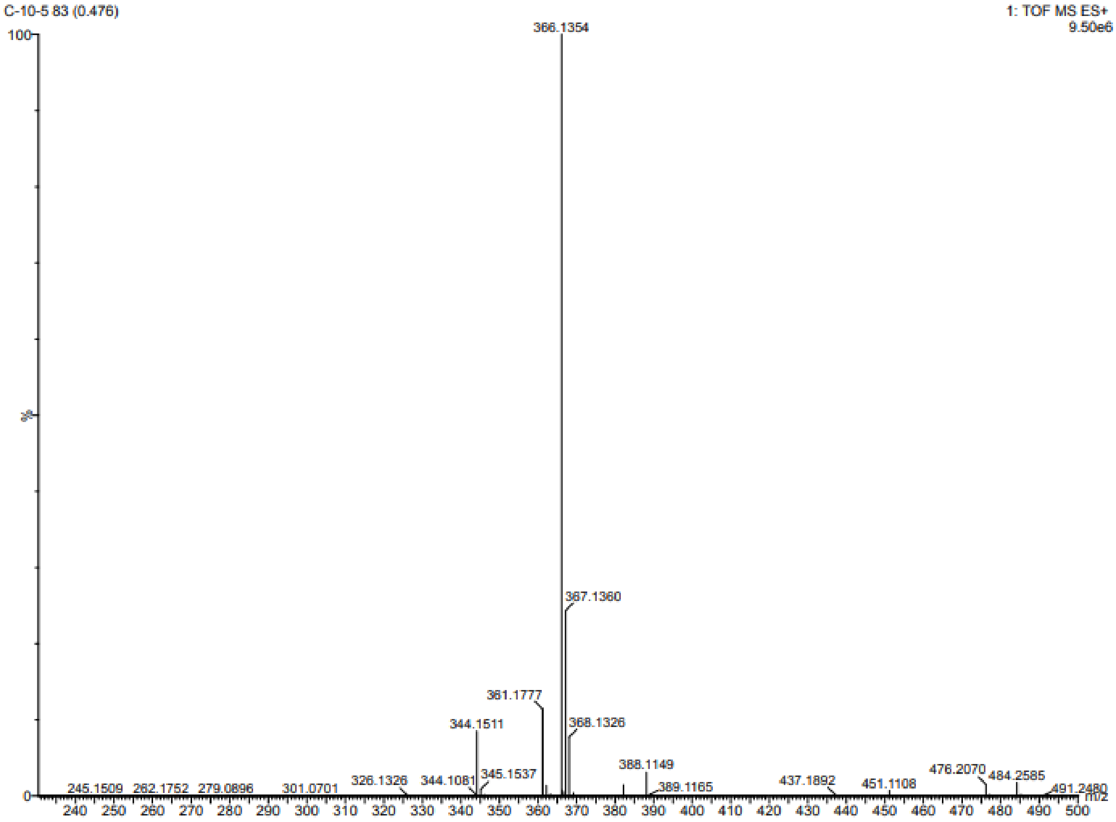

Figure S128. MS of compound *P-36*

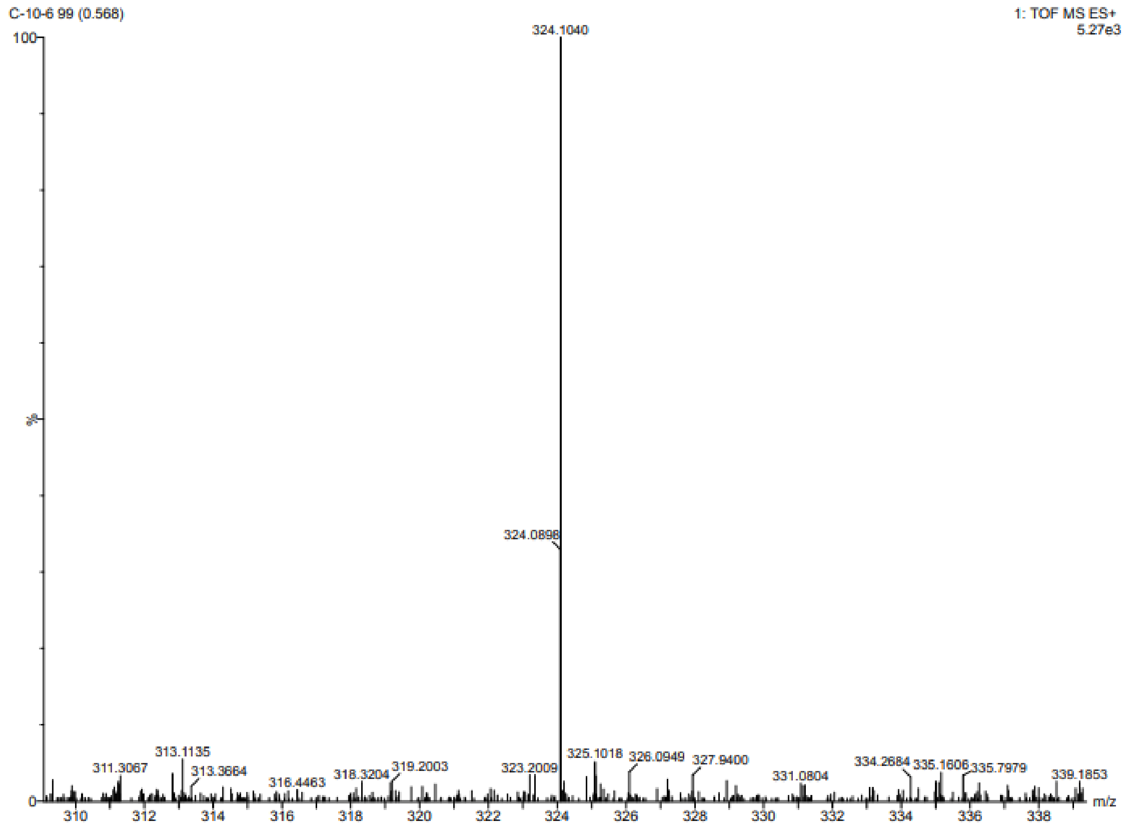

Figure S129. MS of compound *P-37*

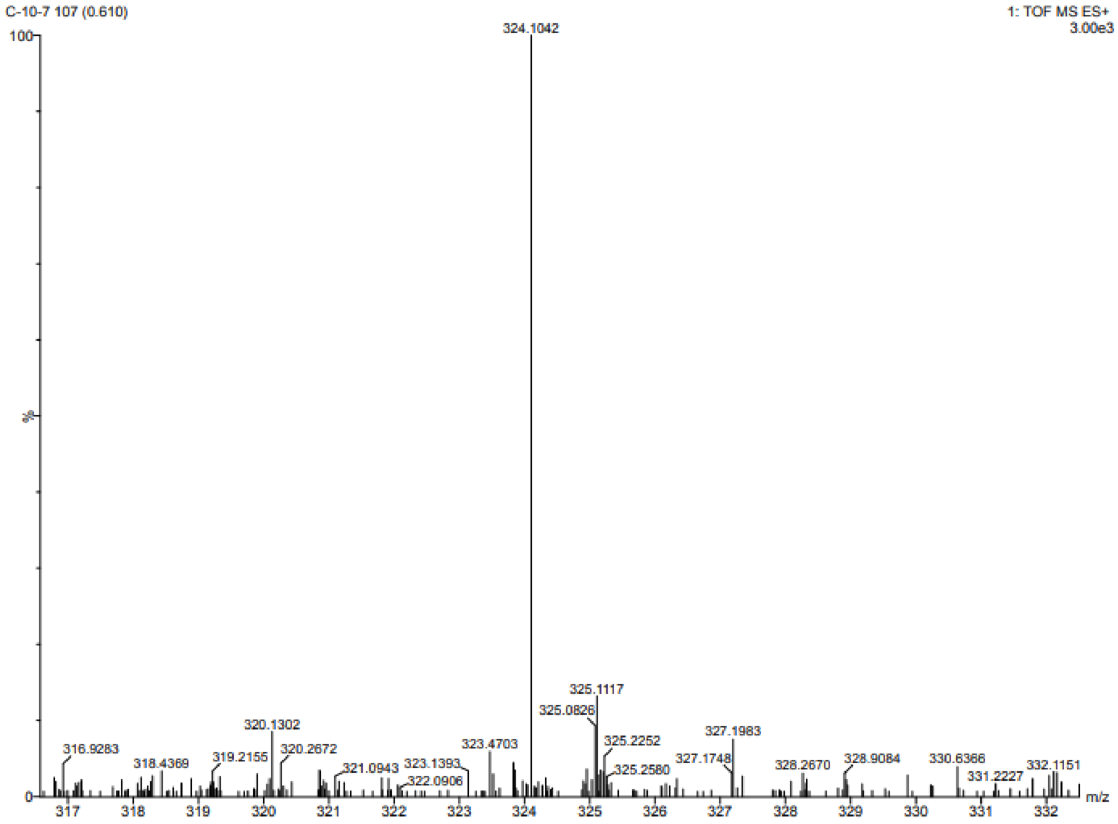

Figure S130. MS of compound *P-38*

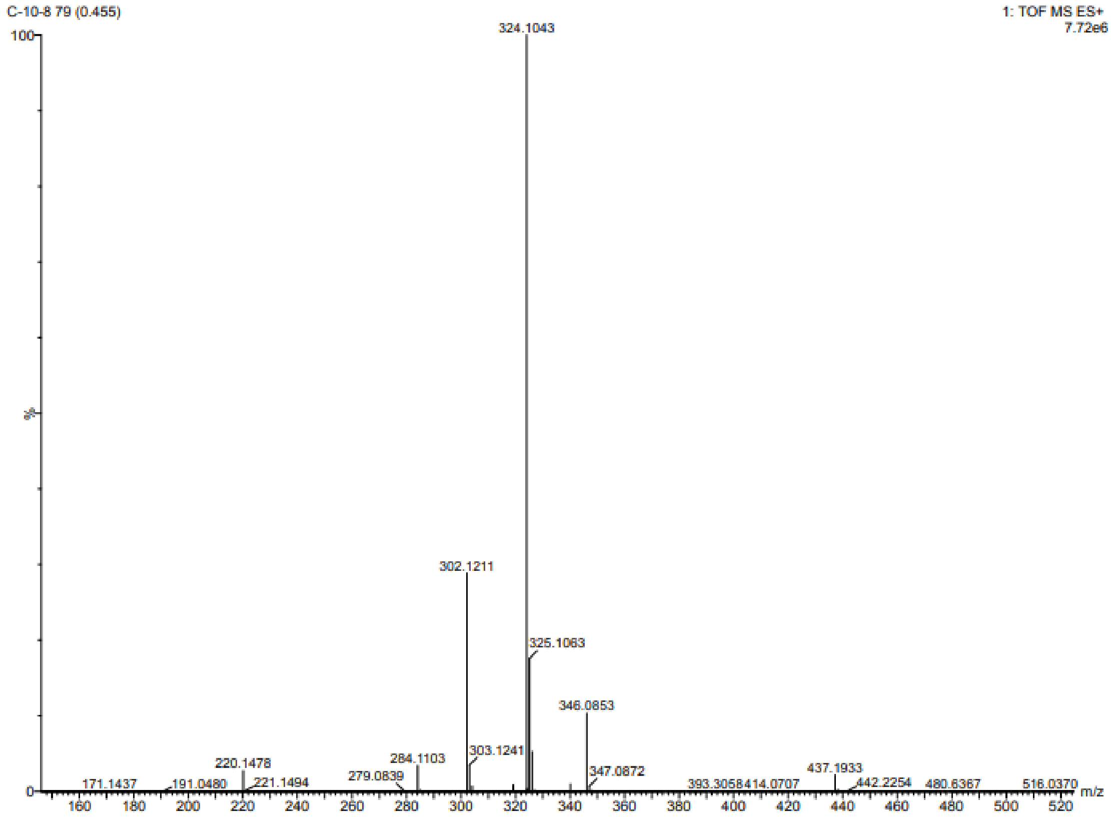

Figure S131. MS of compound *P-39*

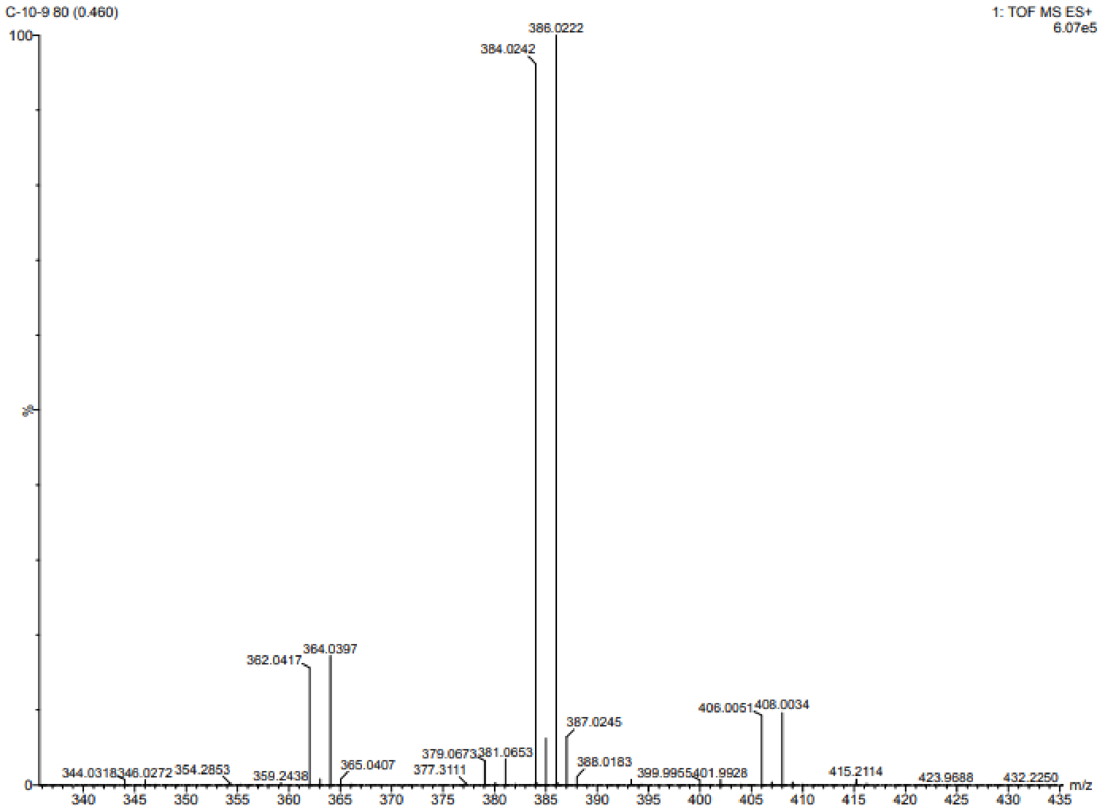

Figure S132. MS of compound *P-40*

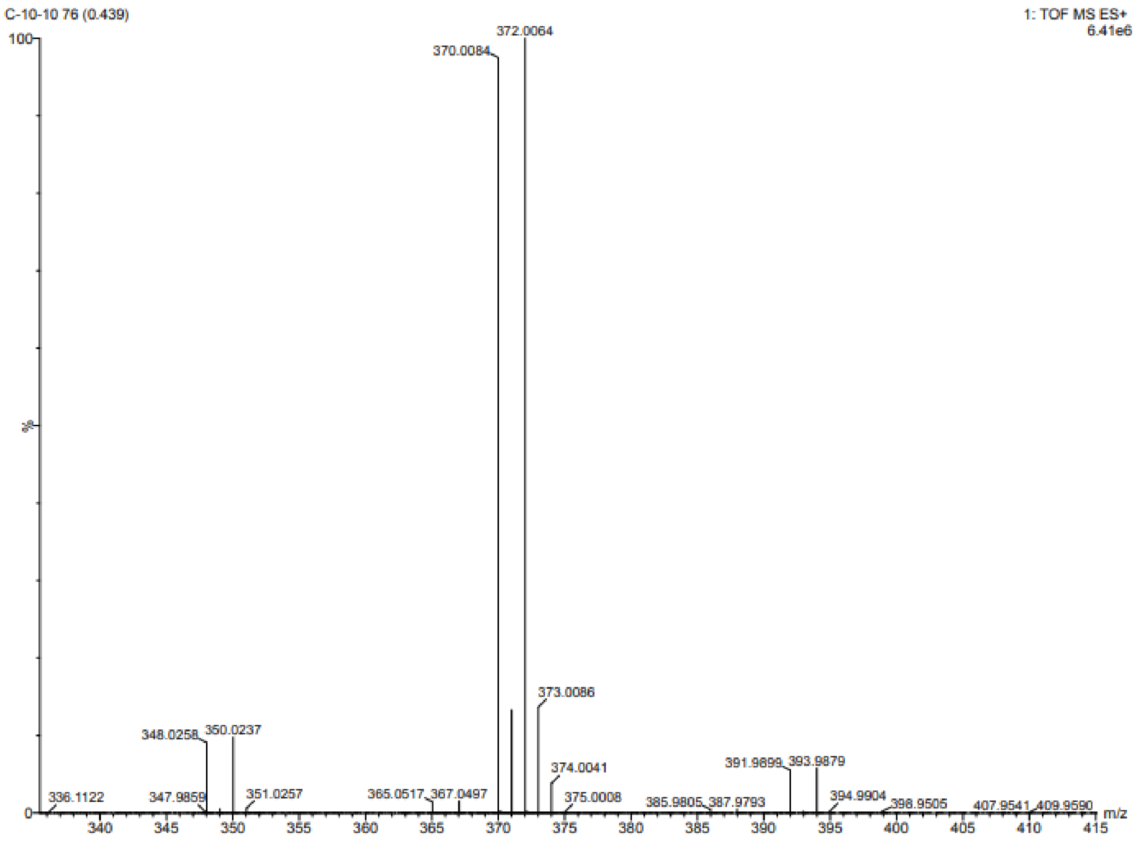

Figure S133. MS of compound *P-41*

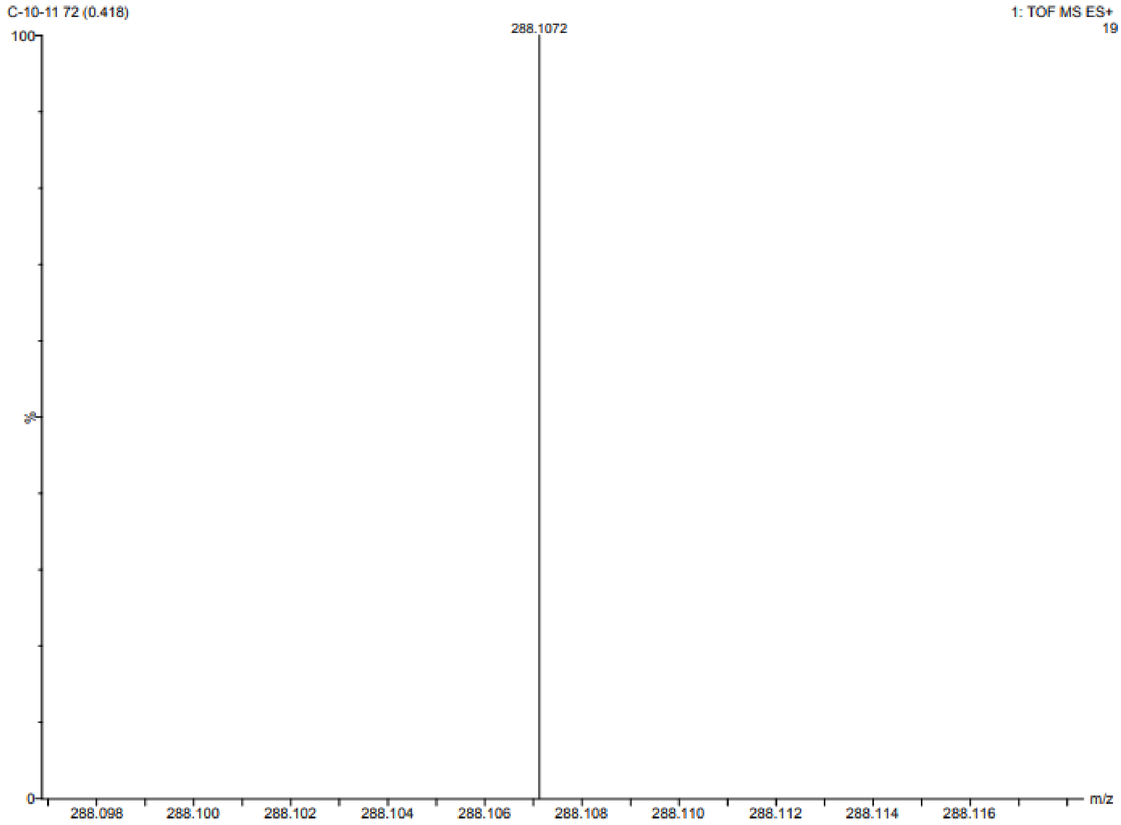

Figure S134. MS of compound *P-42*

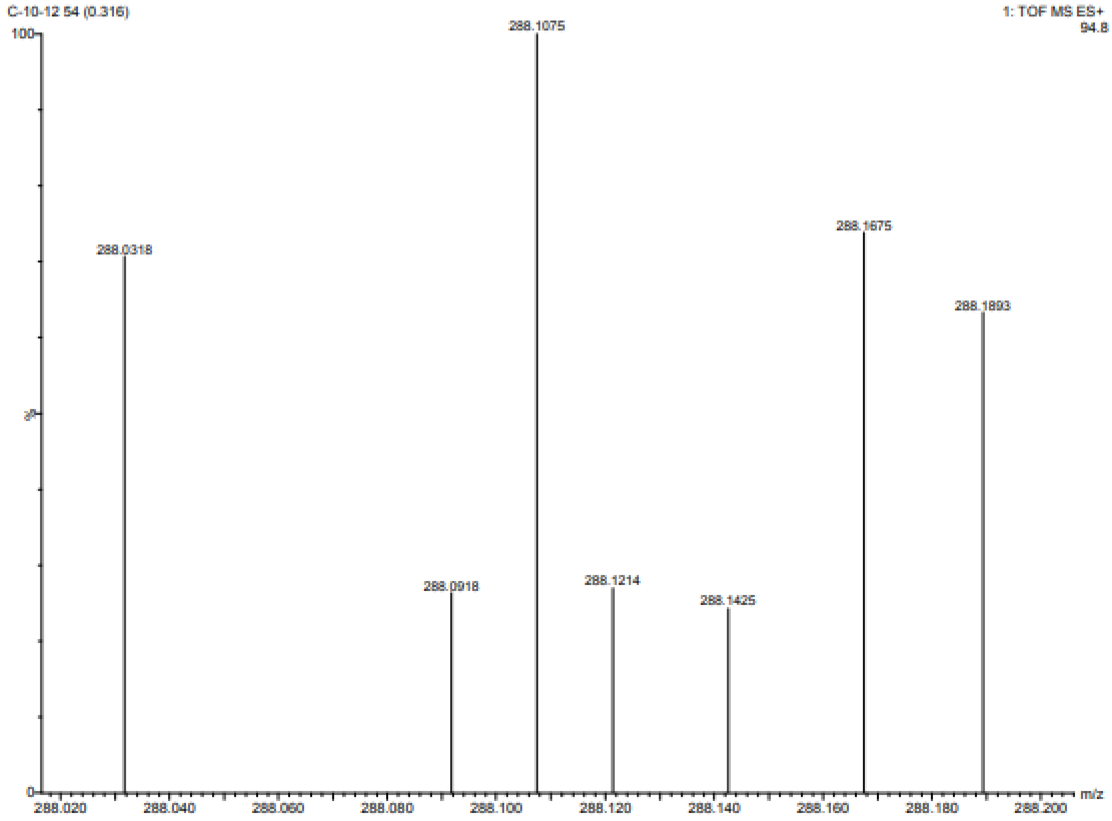

Figure S135. MS of compound *P-43*

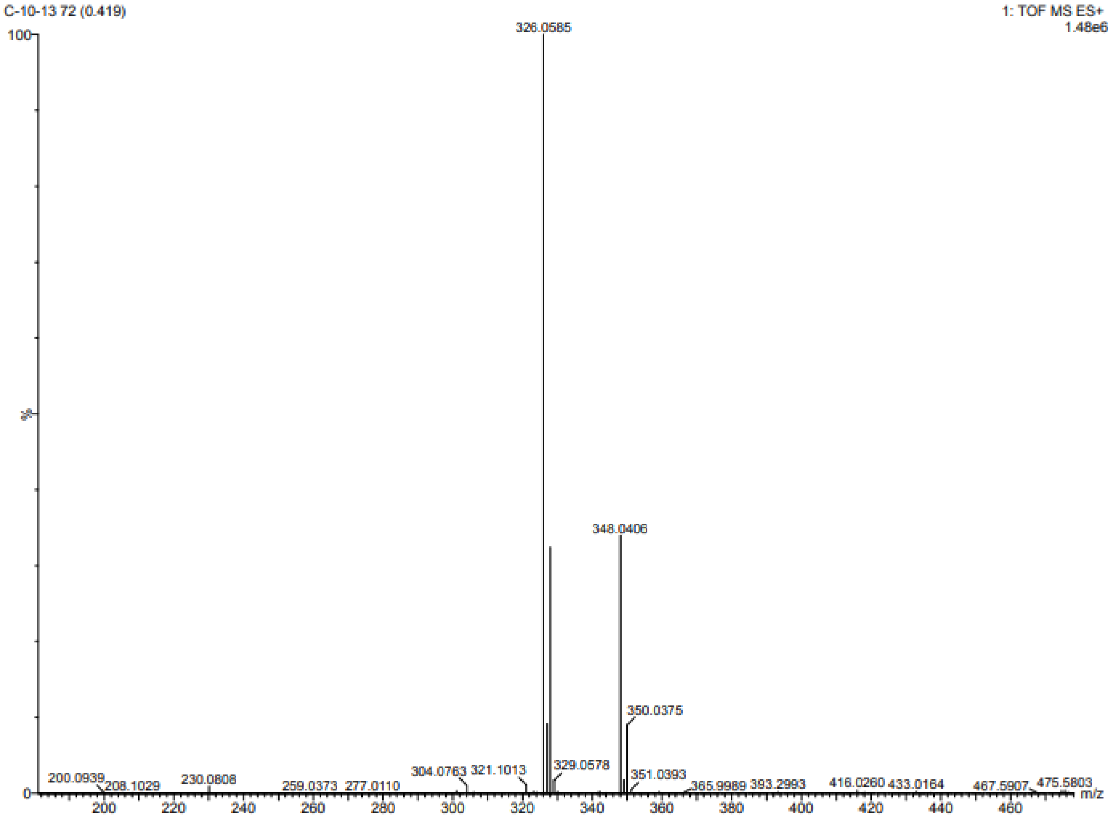

Figure S136. MS of compound *P-44*

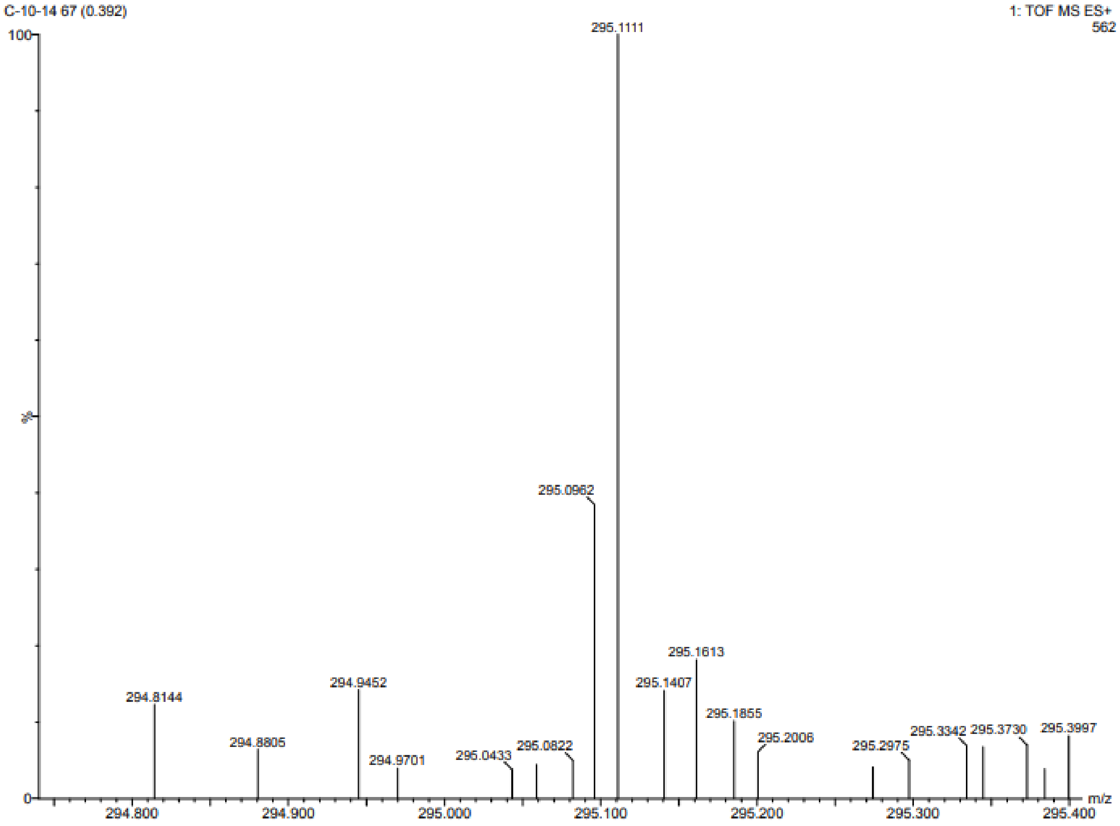

Figure S137. MS of compound *P-45*

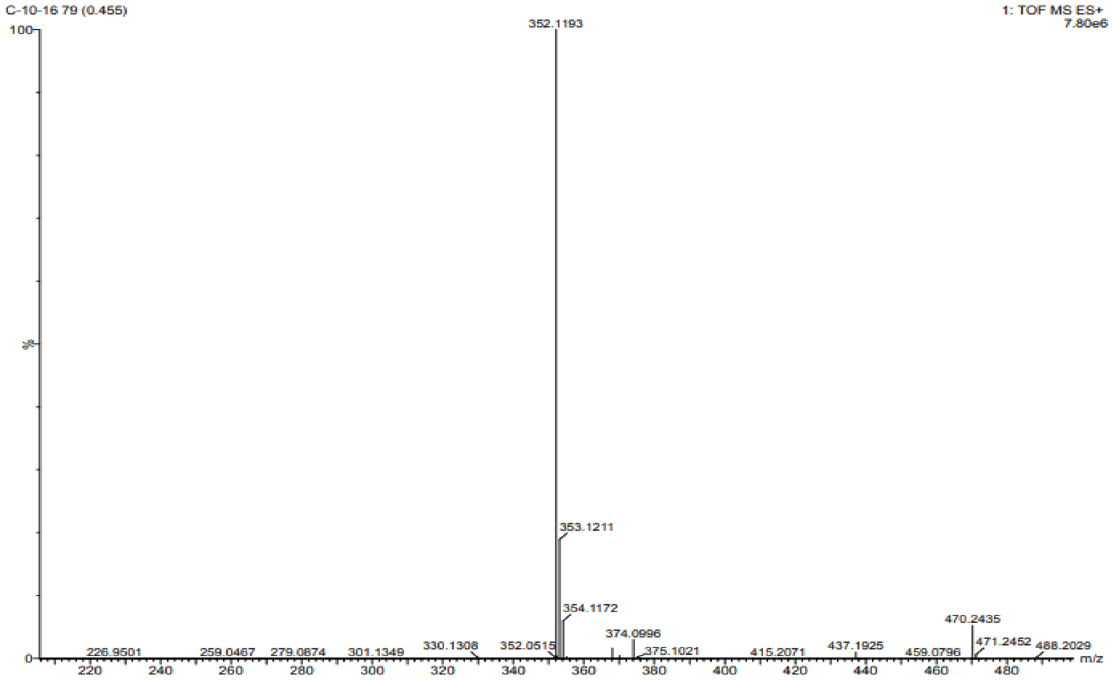

Figure S138. MS of compound *P-46*

3. HPLC spectra of P-18, P-23, P-29, P-30, P-31

The purity of compounds P-18, P-23, P-29, P-30, P-31. were determined by HPLC spectra which were performed on the LC-20A HPLC (inertsil OSD-SP(5um, 2.1*150mm) as stationary phase, 55% methanol as eluent, 225 nm wavelength, 1mL/min flow rate). According to the HPLC spectra, the purity of compounds P-18, P-23, P-29, P-30, P-31 were 97.1%, 98.9%, 87.7%, 95.0%, 99.7%.

P-18

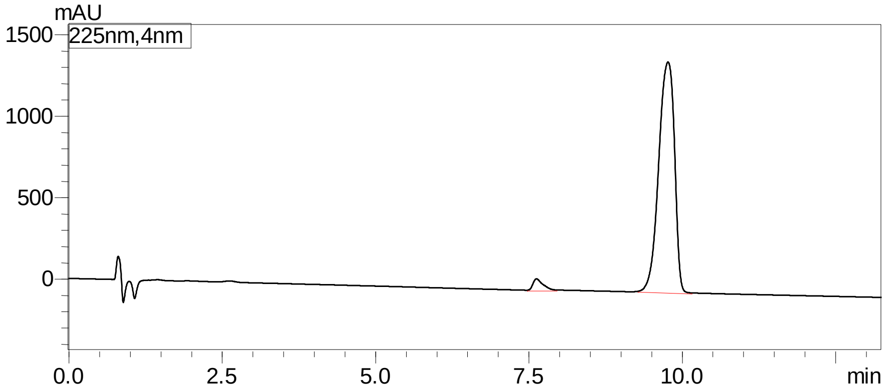

Time (min)	Height (mAU)	Area (mAU*s)	Area (%)
**7.634**	**68822**	**755678**	**2.9**
**9.779**	**1413389**	**25376271**	**97.1**

P-23

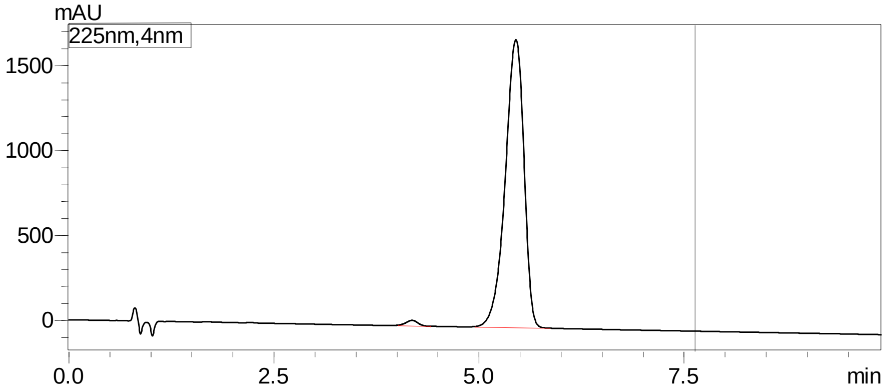

Time (min)	Height (mAU)	Area (mAU*s)	Area (%)
**4.191**	**31269**	**301648**	**1.1**
**5.457**	**1694758**	**26888503**	**98.9**

P-29

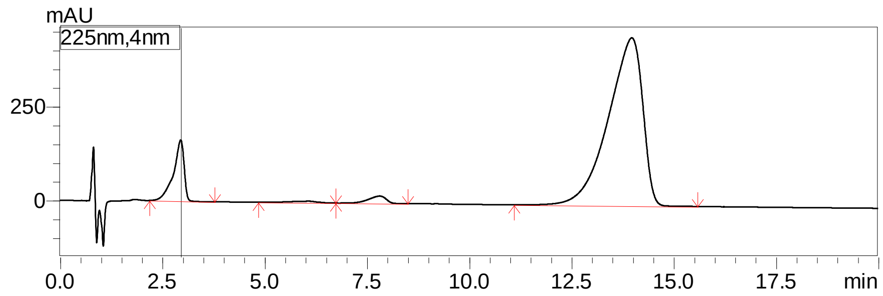

Time (min)	Height (mAU)	Area (mAU*s)	Area (%)
2.952	162759	2922998	9.8
6.067	4170	179920	0.6
7.815	19542	579057	1.9
13.978	447617	26231225	87.7

P-30

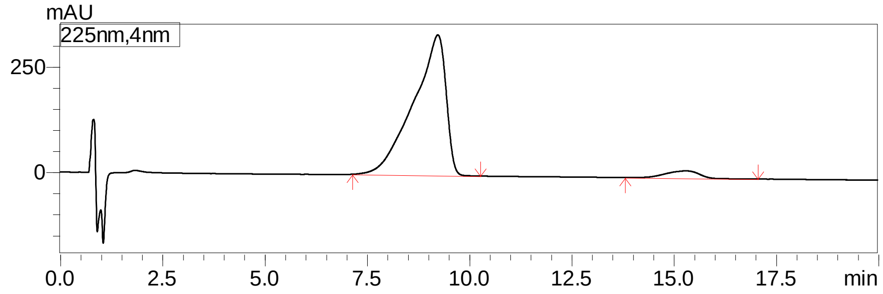

Time (min)	Height (mAU)	Area (mAU*s)	Area (%)
9.238	333029	18354971	95.0
15.287	17329	969544	5.0

P-31

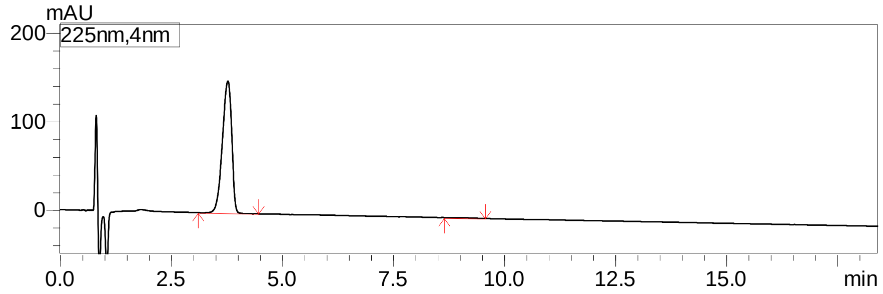

Time (min)	Height (mAU)	Area (mAU*s)	Area (%)
3.782	149264	2184616	99.7
8.65	-9	6812	0.3

4. X-ray single crystal diffraction of P-27

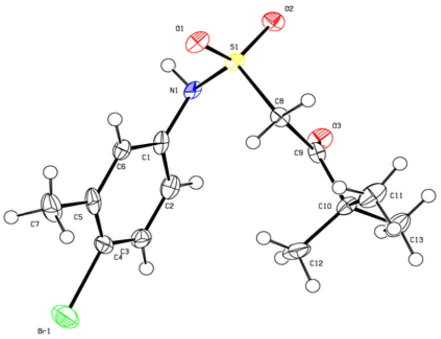

Table S1. Fractional Atomic Coordinates（×10^4）and Equivalent Isotropic Displacement Parameters（Å2×10^3）for *P-27*.

Atom	*x*	*y*	*z*	U（eq）
Br1	1155.4（4）	2754.2（3）	7413.4（5）	43.9（2）
S1	2523.4（8）	7888.6（7）	6054.0（9）	15.2（2）
O1	1920（2）	8278.3（19）	6960（2）	21.8（6）
O2	2831（2）	8605.2（19）	5116（2）	21.0（6）
O3	4235（2）	6650（2）	5302（2）	23.7（6）
N1	1809（2）	6980（2）	4985（3）	16.5（7）
C1	1624（3）	5994（3）	5566（3）	17.7（8）
C2	1924（3）	5098（3）	5002（4）	23.1（9）
C3	1771（3）	4142（3）	5553（4）	23.8（9）
C4	1336（3）	4101（3）	6684（4）	22.0（9）
C5	1019（3）	4974（3）	7276（4）	24.5（9）
C6	1160（3）	5924（3）	6683（3）	18.4（8）
C7	527（4）	4930（3）	8502（4）	34.5（11）
C8	3731（3）	7295（3）	7302（3）	15.8（8）
C9	4431（3）	6653（3）	6619（3）	15.9（8）
C10	5350（3）	5996（3）	7660（4）	18.4（8）
C11	5927（3）	6581（3）	9111（4）	30.2（10）
C12	4775（4）	5013（3）	7954（4）	30.8（10）
C13	6197（3）	5708（3）	6930（4）	29.0（10）

Table S2. Anisotropic Displacement Parameters（Å2×10^3）for *P-27*.

Atom	U_{11}	U_{22}	U_{33}	U_{23}	U_{13}	U_{12}
Br1	37.3（3）	31.9（3）	54.0（3）	15.2（2）	3.5（2）	-8.4（2）
O1	22.0（15）	30.4（15）	12.1（12）	-1.5（11）	4.4（11）	6.8（13）

Atom	U_{11}	U_{22}	U_{33}	U_{23}	U_{13}	U_{12}
O2	24.1 (16)	22.0 (14)	14.3 (12)	2.7 (10)	2.8 (11)	-1.0 (12)
O3	28.6 (16)	32.8 (16)	10.0 (12)	2.5 (11)	7.0 (11)	5.7 (13)
N1	9.3 (16)	21.6 (17)	11.5 (14)	0.7 (12)	-6.1 (12)	1.3 (14)
C1	9 (2)	32 (2)	7.4 (16)	-2.7 (15)	-4.4 (14)	-4.9 (17)
C2	16 (2)	34 (2)	15.9 (18)	-4.7 (16)	1.7 (15)	-1.0 (19)
C3	17 (2)	20 (2)	30 (2)	-2.8 (16)	1.4 (17)	-0.1 (17)
C4	15 (2)	20 (2)	21.9 (19)	4.1 (16)	-5.7 (16)	-5.6 (17)
C5	14 (2)	39 (3)	15.8 (18)	-1.1 (17)	-0.8 (15)	-12.2 (19)
C6	14 (2)	24 (2)	15.0 (17)	-2.8 (16)	2.2 (15)	0.3 (17)
C7	29 (3)	47 (3)	29 (2)	5.4 (19)	12.3 (19)	-12 (2)
C8	17 (2)	19 (2)	6.4 (16)	-4.2 (13)	-2.1 (14)	-4.7 (16)
C9	17 (2)	22 (2)	8.5 (17)	-2.3 (14)	4.2 (14)	-6.0 (17)
C10	16 (2)	21 (2)	16.2 (17)	-2.5 (15)	2.2 (15)	4.6 (17)
C11	26 (2)	40 (3)	16.9 (19)	-5.6 (18)	-4.1 (16)	9 (2)
C12	33 (3)	26 (2)	33 (2)	11.4 (18)	10.6 (19)	15 (2)
C13	25 (2)	43 (3)	20.3 (19)	5.4 (18)	9.1 (17)	12 (2)

Table S3. Bond Lengths for *P-27*.

Atom	Atom	Length/Å	Atom	Atom	Length/Å
Br1	C4	1.911 (4)	C3	C4	1.378 (5)
S1	O1	1.432 (2)	C4	C5	1.378 (5)
S1	O2	1.433 (2)	C5	C6	1.385 (5)
S1	N1	1.624 (3)	C5	C7	1.512 (5)
S1	C8	1.781 (3)	C8	C9	1.515 (5)
O3	C9	1.213 (4)	C9	C10	1.521 (5)
N1	C1	1.436 (4)	C10	C11	1.545 (5)
C1	C2	1.381 (5)	C10	C12	1.533 (5)
C1	C6	1.392 (5)	C10	C13	1.517 (5)
C2	C3	1.379 (5)			

Table S4. Bond Angles for *P-27*.

Atom	Atom	Atom	Angle/°	Atom	Atom	Atom	Angle/°
O1	S1	O2	118.33（15）	C4	C5	C6	116.7（3）
O1	S1	N1	109.84（16）	C4	C5	C7	123.2（4）
O1	S1	C8	104.68（15）	C6	C5	C7	120.0（4）
O2	S1	N1	105.84（14）	C5	C6	C1	121.6（3）

Atom	Atom	Atom	Angle/°	Atom	Atom	Atom	Angle/°
O2	S1	C8	110.39（16）	C9	C8	S1	116.0（2）
N1	S1	C8	107.38（16）	O3	C9	C8	120.8（3）
C1	N1	S1	121.0（2）	O3	C9	C10	122.5（3）
C2	C1	N1	118.6（3）	C10	C9	C8	116.7（3）
C2	C1	C6	119.6（3）	C9	C10	C11	111.2（3）
C6	C1	N1	121.8（3）	C9	C10	C12	105.6（3）
C3	C2	C1	119.9（3）	C9	C10	C13	109.7（3）
C4	C3	C2	119.0（4）	C12	C10	C11	110.3（3）
C3	C4	Br1	117.1（3）	C13	C10	C11	110.0（3）
C3	C4	C5	123.2（3）	C13	C10	C12	110.0（3）
C5	C4	Br1	119.8（3）				

Table S5. Torsion Angles for *P-27*.

A	B	C	D	Angle/°	A	B	C	D	Angle/°
Br1	C4	C5	C6	-179.2（3）	N1	C1	C6	C5	-177.7（3）
Br1	C4	C5	C7	0.2（5）	C1	C2	C3	C4	-1.2（5）
S1	N1	C1	C2	-125.2（3）	C2	C1	C6	C5	1.8（5）
S1	N1	C1	C6	54.3（4）	C2	C3	C4	Br1	-179.4（3）
S1	C8	C9	O3	7.9（5）	C2	C3	C4	C5	1.6（6）
S1	C8	C9	C10	-170.1（3）	C3	C4	C5	C6	-0.3（5）
O1	S1	N1	C1	-68.5（3）	C3	C4	C5	C7	179.1（4）
O1	S1	C8	C9	170.0（3）	C4	C5	C6	C1	-1.5（5）
O2	S1	N1	C1	162.7（3）	C6	C1	C2	C3	-0.5（5）
O2	S1	C8	C9	-61.6（3）	C7	C5	C6	C1	179.1（3）
O3	C9	C10	C11	143.2（4）	C8	S1	N1	C1	44.8（3）
O3	C9	C10	C12	-97.2（4）	C8	C9	C10	C11	-38.8（4）
O3	C9	C10	C13	21.3（5）	C8	C9	C10	C12	80.8（4）
N1	S1	C8	C9	53.3（3）	C8	C9	C10	C13	-160.7（3）
N1	C1	C2	C3	179.1（3）					

Table S6. Hydrogen Atom Coordinates（Å×10^4）and Isotropic Displacement Parameters（Å2×10^3）for *P-27*.

Atom	*x*	*y*	*z*	U（eq）
H1	1190.32	7226.1	4410.03	20
H2	2229.62	5139.54	4251.65	28
H3	1957.54	3533.15	5167.9	29

**H6**	**939.37**	**6528.8**	**7038.47**	**22**
**H7A**	**1052.14**	**4614.82**	**9351.65**	**52**
**H7B**	**-144.11**	**4524.69**	**8194.12**	**52**
**H7C**	**360.76**	**5622.58**	**8739.57**	**52**
**H8A**	**4197**	**7837.24**	**7892.5**	**19**
**H8B**	**3497.83**	**6849.55**	**7958.35**	**19**
**H11A**	**6196.96**	**7239.72**	**8900.2**	**45**
**H11B**	**6539.78**	**6174.01**	**9714.21**	**45**
**H11C**	**5403.28**	**6693.11**	**9618.72**	**45**
**H12A**	**4215.47**	**5204.16**	**8373.93**	**46**
**H12B**	**5315.47**	**4570.77**	**8624.07**	**46**
**H12C**	**4433.37**	**4649.1**	**7048.65**	**46**
**H13A**	**5833.66**	**5339.84**	**6034.8**	**43**
**H13B**	**6761.27**	**5273.72**	**7576.63**	**43**
**H13C**	**6533.56**	**6329.72**	**6715.82**	**43**
